# Correction: Protocol of the Healthy Brain Study: An accessible resource for understanding the human brain and how it dynamically and individually operates in its bio-social context

**DOI:** 10.1371/journal.pone.0267071

**Published:** 2022-04-11

**Authors:** Healthy Brain Study consortium, Esther Aarts, Agnes Akkerman, Mareike Altgassen, Ronald Bartels, Debby Beckers, Kirsten Bevelander, Erik Bijleveld, Esmeralda Blaney Davidson, Annemarie Boleij, Janita Bralten, Toon Cillessen, Jurgen Claassen, Roshan Cools, Ineke Cornelissen, Martin Dresler, Thijs Eijsvogels, Myrthe Faber, Guillén Fernández, Bernd Figner, Matthias Fritsche, Sascha Füllbrunn, Surya Gayet, Marleen M. H. J. van Gelder, Marcel van Gerven, Sabine Geurts, Corina U. Greven, Martine Groefsema, Koen Haak, Peter Hagoort, Yvonne Hartman, Beatrice van der Heijden, Erno Hermans, Vivian Heuvelmans, Florian Hintz, Janet den Hollander, Anneloes M. Hulsman, Sebastian Idesis, Martin Jaeger, Esther Janse, Joost Janzing, Roy P. C. Kessels, Johan C. Karremans, Willemien de Kleijn, Marieke Klein, Floris Klumpers, Nils Kohn, Hubert Korzilius, Bas Krahmer, Floris de Lange, Judith van Leeuwen, Huaiyu Liu, Maartje Luijten, Peggy Manders, Katerina Manevska, José P. Marques, Jon Matthews, James M. McQueen, Pieter Medendorp, René Melis, Antje Meyer, Joukje Oosterman, Lucy Overbeek, Marius Peelen, Jean Popma, Geert Postma, Karin Roelofs, Yvonne G. T. van Rossenberg, Gabi Schaap, Paul Scheepers, Luc Selen, Marianne Starren, Dorine W. Swinkels, Indira Tendolkar, Dick Thijssen, Hans Timmerman, Rayyan Tutunji, Anil Tuladhar, Harm Veling, Maaike Verhagen, Jasper Verkroost, Jacqueline Vink, Vivian Vriezekolk, Janna Vrijsen, Jana Vyrastekova, Selina van der Wal, Roel Willems, Arthur Willemsen

## Notice of republication

This article was republished on April 1, 2022, to update the author byline and list all members of the group consortium. Please download this article again to view the correct version. The originally published, uncorrected article and the republished, corrected articles are provided here for reference.

## Supporting information

S1 File Originally published, uncorrected article. (PDF)

S2 File Republished, corrected article. (PDF)
