# Supplementary material for: Protocol of the Healthy Brain Study: An accessible resource for understanding the human brain and how it dynamically and individually operates in its bio-social context
Source: PLoS One. 2021 Dec 29;16(12):e0260952. doi: 10.1371/journal.pone.0260952 (PMC8716054; doi:10.1371/journal.pone.0260952)
Supplement: S1 File — (DOCX) [file pone.0260952.s001.docx]

**S1. File. Detailed descriptions of measures included in Healthy Brain Study**

**Table of contents**

1. **Burst week: real-world assessments 3**
   1. Real-world assessments 3
      1. Physical activity 3
      2. Stress 4
      3. Sleep 4
   2. Ecological momentary assessments (EMA) 4

**2. Bio-sampling 7**

2.1 Home sampling 7

2.1.1 Stool 7

2.1.2 Urine 7

2.1.3 Saliva 8

2.1.4 Chemicals 8

2.2 Bio-sampling at the lab visit 9

2.2.1 Blood 9

2.2.2 Hair 9

- - 1. Saliva 10

**3. Lab visit: neuroimaging 11**

**4. Lab visit: physiological assessments 15**

- 1. Body composition 15
  2. Blood pressure 15
  3. Physical activity 15
  4. Acute challenge 16

1. **Lab visit: cognitive, affective, and behavioral assessments 17**

5.1 Cognitive assessments 17

5.1.1 Foraging task 17

5.1.2 Serial random-dot motion discrimination task 18

5.1.3 Reward-driven reach-adaptation task 19

5.1.4 Paired associate memory task 19

- - 1. Tower of London 21

5.2 Affective assessments 21

5.2.1 Contextual fear generalization task 21

- - 1. Emotion regulation task 23
    2. Self-referent encoding task 24
    3. Stimulus response compatibility task 25

5.3 Behavioral measurements 26

5.3.1 Columbia card task 26

- - 1. Food auction task 27

1. **Lab visit: sensory tests 29**

6.1 Vision 29

6.2 Hearing 29

**7 Post-visit online assessments 30**

7.1 Decision making 30

7.1.1 Trust game 30

7.1.2 Equality equivalence test 31

7.1.3 Higher-order risk preferences 31

7.1.4 Time preferences 32

7.1.5 Public good game 33

7.1.6 Ambiguity 34

7.1.7 Incentives 35

- 1. Narrative reading 36

7.3 Solidarity 38

1. **Individual differences in language skills test battery 41**

8.1 Stairs4Words 43

8.2 Peabody picture vocabulary test 45

8.3 Idiom recognition test 45

8.4 Spelling test 46

8.5 Author recognition test 46

8.6 Prescriptive grammar test 46

8.7 Auditory simple reaction time test 47

8.8 Auditory choice reaction time test 48

8.9 Letter comparison 48

8.10 Visual simple reaction time test 49

8.11 Visual choice reaction time test 49

8.12 Digit span (forward and backward) 50

8.13 Corsi block clicking (forward and backward) 50

8.14 Raven’s advanced progressive matrices test 51

8.15 Picture naming 51

8.16 Rapid automatized naming (RAN) 52

8.17 Verbal fluency 53

8.18 Antonym production 54

8.19 Maximal speech rate 54

8.20 Phrase generation 54

8.21 Sentence generation (structured) 56

8.22 Sentence generation (unstructured) 57

8.23 Spontaneous speech 58

8.24 Non-word monitoring in noise in non-word lists 59

8.25 Rhyme judgment 60

8.26 Auditory lexical decision 61

8.27 Semantic categorization 62

8.28 Word monitoring in noise in sentences 63

8.29 Gender cue activation during sentence comprehension 65

8.30 Verb semantics activation during sentence comprehension 66

8.31 Self-paced reading 68

1. **References 72**

**1 Burst week: real-world assessments**

The burst week consists of real-world assessments (Chapter 1.1) of physical activity, stress, and sleep using validated wearable devices and of ecological momentary assessments (EMA; Chapter 1.2) using a smartphone application. In addition, participants perform home sampling of stool, urine, saliva, and chemicals during the burst week (Chapter 2.1). Fig 1 presents an overview of the burst week.

Fig 1. Overview of burst week.

- 1. **Real-world assessments**
     1. **Physical activity**

Recordings of physical activity enable assessment of real-world (in)activity as well as control for movement when assessing physiological responses to stress. How much a participant moves can affect how the brain works. Sedentary behavior and physical acitivity characteristics (i.e. sedentary, standing and stepping time, and Metabolic Equivalent of Task (MET) values) are measured using the activPAL4 micro (PAL Technologies Ltd., Glasgow, UK) [1, 2]. Participants attach the waterproof device on the midline, one third of the way down on the right thigh using a breathable hypoallergenic dressing. Participants wear the monitor 24-hours/day over an eight-day period. The monitors are initialized and downloaded using the activPAL software 8.11.1.47 (PAL Technologies Ltd., Glasgow, UK). The raw data is analyzed by a modified version of the script of Winkler *et al*. [3]. Sitting time is defined as any waking behavior characterized by an energy expenditure ≤ 1.5 MET, while in a seated, reclined or lying posture. Light intensity physical activity (LIPA) is defined as standing time combined with stepping time with MET-values < 3. Moderate-to-vigorous physical activity (MVPA) is defined as stepping time with MET-values ≥ 3. The accumulation of sedentary time is defined as short (< five consecutive minutes), medium (5-29 consecutive minutes) and prolonged (≥ 30 consecutive minutes) sedentary bouts.

- - 1. **Stress**

Physiological recordings allow for assessment of autonomic nervous system parameters relevant for stress. Participants wear the Empatica E4 wristband (Empatica Inc., Milan, Italy) [4] for seven days, 23 hours a day, to assess heart rate (variability), skin conductance, and skin temperature.

Participants put the watch on their non-dominant hand. The device must be charged for one hour every day. Participants stop wearing on the morning of their lab visit when they get up.

- - 1. **Sleep**

Physiological recordings of sleep are required because sleep is both symptom and causal factor for health conditions, cognitive function, affect/mood, and stress coping [5]. Participants wear the Z-max sleep band (Hypnodyne, Sofia, Bulgaria) for seven nights. The device measures whole-night sleep EEG via two latero-frontal channels and a fronto-central reference.

Participants place the sleep band in the center of the forehead and slightly above the eyebrows. All parts of the hydrogel electrode patch should be flat against the skin. Participants switch on the sleep band, lie down on their back and follow sleep instructions.

**1.2 Ecological momentary assessments (EMA)**

The questionnaire used for EMA covers mood, social company, online social interactions, context, control items, retrospection, anticipation, and substance use. The questionnaire contains the following mood items: ‘I feel happy/ annoyed/ fearful/ satisfied/ insecure/ lonely/ relaxed/ suspicious/ sad/ stressed/ restless/ fine/ focused/ self-confident’. Participants score all of these items on a 7-point Likert scale. Then, it contains social items: ‘my company consists of partner/ children/ family/ friend(s)/ colleague(s)/ other familiar people/ unfamiliar people/ nobody’ and the degree to which the (absence of) company is appreciated. Also, online social interactions are assessed. Items of the context include: ‘spare time active/ spare time passive/ in contact with people/ listening music/ working or studying/ household or caring/ eating/ traveling/ personal care/ sleeping/ nothing/ something else’, the degree to which the activity is being appreciated and where the activity is taking place. Control items are tiredness, hunger, physical health, and pain. Next, the questionnaire contains items about the degree of pleasure of the most significant event (can be either negative or positive) since the previous questionnaire and the degree of eagerness to the most significant event in the coming two hours. Finally, substance use (medication/ coffee/ sugar containing sodas/ alcohol/ cigarettes/ cannabis/ other drug/ nothing) since the previous questionnaire is assessed.

The questionnaire is sent by a smartphone app. Table 1 presents the details of the implementation of EMA in the HBS.

Table 1. Details of EMA in the HBS.

| Number of sampling periods | 3 |
| --- | --- |
| Sampling period | 6 consecutive days |
| Sampling frequency | 10 |
| Time interval between first and last assessment within a day | 12,5 hours |
| Time of the start of the assessments within a day | 7:00am |
| Time of the end of the assessments within a day | 10pm |
| Questionnaire length, number of items | 38 items |
| Approximate mean duration of the questionnaire | 3 minutes |
| Sampling method | Semi-random |
| Time interval between questionnaires* | 20-90 minutes |
| Time to answer questionnaire* | 15 minutes |
| Type of device | Smartphone: Android or IOS |
| Questionnaire sent by | Push notification by sound and/or vibration |
| Scale | 7-point Likert scales and nominal (yes/no) |
| Feedback on compliance* | Daily by push notification at start of the day |
| Value of incentive | 150 euro for completing the full study, but no specific incentive for EMA |

**After the first 98 participants had started, we adapted the time interval between questionnaires (we started with 15-90 minutes) and the time to answer the questionnaires (we started with 10 minutes) and started to give feedback on compliance (we started without feedback).*

1. **Bio-sampling**

Participants perform home sampling of stool, urine, saliva, and chemicals during the burst week (Chapter 2.1). In addition, during the lab visit at the Radboud campus we collect samples of blood, hair, and saliva (Chapter 2.2).

**2.1 Home sampling**

**2.1.1 Stool**

Over 10^13^ bacteria, viruses and fungi colonize our gastrointestinal tract, of which most of them are found in the colon. They help digest our food, and produce all kinds of essential nutrients and chemicals that affect our body and even our brain [6].

Participants collect fresh stool up to 24 hours before the lab visit. After collecting the sample, participants place the container with stool in a plastic bag with a lock strip in the refrigerator and take it to the lab visit in a safety bag with a cooling element. Participants fill out a stool collection questionnaire about medication use and stool frequency and composition (Bristol stool chart) [7].

The Radboud Biobank [8] handles and stores the stool samples in six aliquots in a freezer at -80 degrees Celsius.

**2.1.2 Urine**

Urine contains waste products, which we excrete via the urinary tract. Urine includes water and salts. The composition of urine can provide information about fluid balance and metabolism, which in turn affects our overall health, including our brain.

Participants collect morning urine, which is the first urine passed on the morning of the lab visit. After collecting the sample, participants place the tube in a plastic bag with a lock strip in the refrigerator and take it to the lab visit in a safety bag with a cooling element.

The Radboud Biobank [8] handles and stores the urine samples in six aliquots in a freezer at -80 degrees Celsius.

**2.1.3 Saliva**

The stress hormone cortisol can be measured in saliva [9]. Participants take two samples at home at the time indicated by the research team. The time is similar to the time of the start of the acute challenge during the lab visit (Chapter 4.4). Thereby, these samples serve as reference as cortisol levels vary throughout the day [10].

Participants put on gloves to avoid touching the cotton swab with bare hands. They place the cotton swab in the mouth, move it back and forth for three minutes, chewing is recommended. After collecting the sample, participants keep the saliva samples at room temperature.

We store the saliva samples in a freezer at -20 degrees Celsius until analysis in batches. After thawing, salivettes are centrifuged at 3,000 rpm for five minutes, which results in a clear supernatant of low viscosity. Salivary concentrations are measured using commercially available chemiluminescence immunoassay with high sensitivity (IBL International, Hamburg, Germany).

**2.1.4 Chemicals**

Silicon bands are used as diffusive sampling devices to collect (semi)volatile organic compounds participants come into contact with [11, 12]. Substances from the environment or from products that they use can affect the functioning of the brain.

Participants receive two silicon bands in glass tubes with plastic screw cap. They wear one band on the wrist of their dominant hand for seven days and nights. The second band is placed at a fixed location in their living room for the entire seven-day period. The band hangs on a nail or hook on the wall or on top of a cupboard or bookshelf. Participants are recommended not to hang the band in front of a window or door or in front of a ventilation opening, in a houseplant or above a heat source (e.g., lamp, stove, burning candle). On the morning of the lab visit, participants return the bands in the tubes.

We store the silicone bands in a freezer at -20 degrees Celsius until analysis. After thawing, we extract the substances of interest for chemical analysis in the laboratory.

**2.2 Bio-sampling at the lab visit**

**2.2.1 Blood**

To assess general health, genetic make-up, as well as markers that may have specific relations to the functioning of the brain, we take fasten blood samples: 89.5 ml at the first lab visit and 83.5 ml at the second and third lab visit as collection for DNA is only required once.

The Radboud Biobank [8] handles the blood samples and stores DNA stock, 3 PAX gene tubes, 24 aliquots plasma EDTA, 6 aliquots serum, 12 aliquots plasma heparin in a freezer at -80 degrees Celsius.

**2.2.2 Hair**

To measure a temporal gradient of total cortisol release, a small bundle of hair is cut [13]. Hair grows on average by one cm per month. Cortisol analysis can be done time reliably for up to three months. This requires a three cm section. No hair sample can be taken if the hair is to short or absent. The further away the hair is from the scalp, the more it is exposed to external factors such as washing (shampoos with alcohol), sunlight, damage, etc. which is likely to affect the measured cortisol concentration.

We cut the three-cm-strand of hair in the middle of the back of the head and as close to the scalp as possible.

We store the hair samples at room temperature until analysis.

**2.2.3 Saliva**

Saliva samples for cortisol are collected before, immediately after and twenty minutes after the acute challenge test (Chapter 4.4) to capture the short-term dynamics of the stress response [9].
The collecting, handling, and storing of samples is similar to the home samples (Chapter 2.1.3).

**3. Lab visit: neuroimaging**

State-of-the-art, high-quality magnetic resonance imaging (MRI) data are collected to enable studies into the structural and functional organization of the healthy human brain, its variation across individuals, and establish links with the non-imaging measures acquired in the study. The imaging measures are acquired during 45-minutes MR sessions and have been selected to integrate well with secondary resources, such as the Human Connectome Project [14], and the UK Biobank brain imaging project [15], as well as local efforts such as the Personalized Parkinson Project [16]. As such, the MR sequences have been tailored to match the sequences of these secondary resources as much as possible. Importantly, the selected imaging measures probe brain structure and function as broadly as possible to enable the widest possible range of research avenues and deliver data with the broadest possible predictive power for mental and physical health. To make this work within the time constraints of 45-minute sessions, we limited the number of repeats of some of the structural MRI acquisitions because they are expected to exhibit little dynamic variation within the one year time period of interest.

The neuroimaging data are acquired with two nearly identical 3T MR scanners: a Siemens Prisma and a Siemens Prismafit. Prior to data collection, participants are randomly assigned to one of these scanners and all MR measurements across all MR sessions (i.e. lab visits) are collected using the assigned equipment. All MR sessions include the following scans: an anatomical MRI scan (T1w 3D MPRAGE); a 10-minute resting-state functional MRI (rfMRI) scan; a 4.5-minute movie functional MRI (mfMRI) scan and approximately two minutes of auxiliary scans (e.g., scout, field map, single-band reference EPIs). In addition to these repeated scans, we acquire in each session either a high-resolution T1w 3D MP2RAGE anatomical scan for quantitative T1 and cortical myelin mapping, a high-resolution multi-echo gradient echo T2* weighted sequence for the identification and quantification of iron deposition using Quantitative Susceptibility Mapping (QSM), and a diffusion weighted imaging (DWI) scan to enable structural connectivity characterizations and white matter microstructural tissue modelling. In addition to the MR scans, each participant undergoes a fifteen-minute practice session in the mock scanner prior to the first MR session so as to acquaint themselves with the scanner environment and learn how to minimize head movements.

The T1w MPRAGE scans allow for normalization to a template brain, as well as accurate identification of cortical and sub-cortical grey-matter, which is crucial for many anatomical (e.g. voxel-based morphometry, identification of anatomical pathologies) and functional brain imaging studies (e.g. for accurate surface-based registration procedures or to create a model of the cortical sheet that can be inflated to reveal the spatial layout of functional activation patterns that would be difficult to discern). Repeated scans further enable tracking various morphometric and tissue characterizations over time.

The rfMRI data allows for characterizing functional connectivity in the brain as well as for delineating functional brain regions. It relies on measurements of spontaneous fluctuations in brain activity that occur when participants are at rest, and can be applied with the same relative ease as structural MRI. Being a measure of function, it is expected that the rfMRI data will be more sensitive to subtle inter-individual variations in behavior and lifestyle across time. By combining the rfMRI data with the structural MRI measurements it is further possible to obtain individualized delineations of more than 150 distinct functional brain areas [17]. The rfMRI scan involves a version of the multiband accelerated EPI sequence that has also been used in the Human Connectome Project [14] and the UK Biobank brain imaging study [15] and is considered to be the current state-of-the-art in rfMRI data acquisition. In addition to exceptional data quality, its main benefit concerns the high temporal resolution to adequately sample nuisance signal components like cardiac and respiratory responses, which can thereby be identified and removed from the data. Directly after the rfMRI scan, participants fill out a modified version of the Amsterdam Resting-State Questionnaire (ARSQ [18]) to gauge the participant’s resting-state cognition along dimensions of discontinuity of mind, theory of mind, self, planning, sleepiness, comfort, and somatic awareness.

During the mfMRI scan, participants watch and listen to movie clips, which engages a range of neural systems, from low level sensory areas to those involved in more abstract higher-level functions such as language and emotion processing [19]. Three 1.5-minute movie clips—one about climate change, one about immigration, and one about health care—are shown during each 5-minute session in pseudorandomized order determined prior to the first lab visit. During each lab visit, each movie clip is shown once with one of three possible aurally presented narratives: blame framing, threat framing and neutral framing. The mfMRI scans will use the same multiband accelerated imaging protocols as the rfMRI scans.

The T1w 3D MP2RAGE scan [20] complements the more common MPRAGE sequence described above. While the standard MPRAGE scan offers excellent T1-weighted contrast between the brain’s grey matter, white matter, and cerebral spinal fluid in a time-efficient manner, it does not provide the quantitative information needed for, for instance, myelin mapping. Myelin is an insulating, fatty sheet surrounding nerve fibers that acts to speed up signal processing in the brain and the loss of myelin can cause various problems due to the slowing of nerve impulses and ‘short-circuiting’. By including the T1w MP2RAGE scan, it will be possible to study how regional myelination levels are related to the health of functions those neural pathways subserve. In addition, some of the brain’s functional areas can be better defined using myelin maps, adding to the quality of ensuing functional parcellation and functional connectivity analysis.

QSM refers to post-processed susceptibility-weighted MR images, which can be used to map iron deposition in the brain [21]. Iron is vitally important in many aspects of normal brain functions through its role in the synthesis of adenosine triphosphate (ATP) in mitochondria as well as myelin. Excessive iron accumulation, however, leads to neurodegeneration and cognitive decline. Iron deposition in the brain is therefore expected to be an important marker for healthy brain function which can be probed by the inclusion of QSM as one of the MR measures.

Diffusion MRI (dMRI) enables deriving estimates of local tissue microstructure and tracing long range white matter pathways. As for the rfMRI data, the dMRI are collected with state-of-the-art multiband accelerated imaging to substantially reduce the acquisition time without signal-to-noise ratio and resolution trade-offs or a loss in the number of voxel-wise diffusion data points (more diffusion data points yield increased accuracy in estimating white-matter fiber orientations). As in the UK Biobank, this allows for advanced model fitting of microstructural parameters that would not have been possible under the present time constraints with less advanced previous technology [15]. Examples of dMRI modeling outputs are: FA (fractional anisotropy), MD (mean diffusivity), MO (tensor mode), ICVF (intra-cellular volume fraction), ISOVF (isotropic or free water volume fraction) and OD (orientation dispersion index). These measures can then be quantified for individual white matter tracts, which can also be defined using the dMRI data and by themselves additionally offer structural connectivity information.

**4 Lab visit: physiological assessments**

**4.1 Body composition**

Height, weight, waist and hip circumference are measured using standard procedures. Body fat, fat weight, total body water, skeletal muscle mass, body fat mass index, and fat free mass index is determined using multiple-frequency bioelectrical impedance spectroscopy (Multi-Scan 5000, Bodystat Ltd., UK) [22]. After a three-minute rest, participants are measured in supine position with the limbs slightly abducted. Four skin electrodes are attached to the body: two at the dorsal sight of the right hand at the metacarpals and wrist and two at the right foot at the metatarsals and ankle. Bioelectrical impedance is measured by passing a mild electrical current (800uA) across a range of frequencies (5-500Hz).

**4.2 Blood pressure**

Blood pressure is assessed by an automated blood pressure cuff (Dinamap Carescape V100, GE Healthcare, Finland) at the left upper arm in sitting position [23].

- 1. **Physical activity**

The Åstrand-Rhyming test is used as a submaximal cycling test to estimate physical fitness, expressed as estimated maximal oxygen consumption [24]. In short, participants perform a six-minute cycling test, preceded by a two to three-minute warm-up phase. During warming-up, workload is gradually increased to ensure that heart rate maintained at a stable level between 120 and 150 b/min during the fifth and sixth minute of the cycling test. Based on gender, workload, weight, and heart rate at minute five and six maximal oxygen consumption can be estimated. Heart rate is monitored continuously during testing by Ergoline heart rate monitors (Ergoline, Bitz, Germany).

- 1. **Acute challenge**

To capture short-term physiological dynamics, participants undergo an acute challenge, the cold pressor test, in which they are instructed to submerge their hand to the wrist for up to three minutes into cold water (one to four degrees Celsius).

First, non-invasive ultrasound is used to measure carotid artery reactivity (CAR%). Resting and peak carotid artery diameters during cold pressor test are measured and result in CAR% as relative change from baseline [25]. CAR% has strong similarity with coronary artery reactivity and has prognostic value for future cardiovascular events [26, 27]. Second, to systematically capture how the individual’s stress system responds to (and recovers from) an acute challenge, saliva samples for cortisol are collected before, immediately after and twenty minutes after the test to capture the short-term dynamics of the stress response [9]. To ensure a robust stress-induced increase in salivary cortisol, social evaluation is required [9]. Therefore, the participant is told that facial expressions are analyzed by a video recording. Also, heart rate and subjective stress is assessed. Third, to assess subjective pain levels, participants rate their pain using a visual analogue scale [28, 29] immediately after withdrawal of their hand from the water. To assess objective pain levels, pressure pain thresholds are determined before and after the test. The QST-3 device (JNI Biomedical ApS, Larup, Denmark) is used to measure electrical pain thresholds (EPTs) on the non-dominant lower arm [30]. Measurement location is sixteen centimeters and twelve centimeters from the processus syloideus ulnae. Two measurements are performed and thresholds are assessed and expressed in milli-Ampere. The electrical pain tolerance threshold (EPTT) is determined when the current is as high as the participant can tolerate.

1. **Lab visit: cognitive, affective, and behavioral assessments**

The tasks described in this chapter are performed in a cubicle. All visual stimuli are generated with the Psychophysics Toolbox (Brainard, 1997) for MATLAB (MathWorks, Natick, MA) and are displayed on a 24-inch flat-panel display (Benq XL2420T, resolution: 1,920 x 1,080, refresh rate: 60 Hz). Participants view the stimuli from a distance of 53 cm in a dimly lit room. In case additional equipment is used, it is described below.

**5.1 Cognitive assessments**

**5.1.1 Foraging task**

In one class of decision problems, choice options are presented not simultaneously, but serially. For these decision problems, the decision is between sticking with the current option—or to move on, in search for a better one. To assess how people make such decisions, we use the foraging task that was previously described in Constantino *et al*. [31]. This task is introduced to the participants as a tree-harvesting task. Specifically, on each trial of this task, participants are exposed to an apple tree. On every trial of this task, participants can either harvest the tree, or travel to the next tree. If participants choose to harvest, they incur a short harvest time delay (the harvest time) and they earn a number of apples. With every time participants harvest the same tree, the tree returns exponentially less apples. If participants choose to travel to the next tree, they incur a longer delay (the travel time) and a new, replenished tree appears. Throughout the task, participants experience two different foraging environments. One foraging environment is characterized by a long travel time; the other foraging environment is characterized by a short travel time. Participants visit each foraging environment twice, for six minutes per visit, in an ABAB or BABA order. Each foraging environment has a separate, distinct background color, so that participants can easily distinguish them from one another.

Our task is identical to the one described in Constantino *et al*. [31], except for three changes. First, we use different timing parameters (harvest time is three seconds; travel time is nine seconds or four seconds, depending on the foraging environment). Second, we use instructions that (a) are slightly abridged and (b) are translated into Dutch. Third, at the end of the session, we convert apples to a monetary reward at a rate of 0.0025 euro per apple.

**5.1.2 Serial random-dot motion discrimination task**

We use a random dot motion discrimination task [32, 33] to quantify choice history biases during perceptual decision-making. Random dot kinematograms consist of 770 white dots on a black screen, presented within a circular aperture (12° radius). The dot density is 1.7 dots/deg2. The entire population of dots is split into “signal dots” and “noise dots”. The signal dots move either upward or downward with a velocity of 11.5°/s. The noise dots change position randomly from frame to frame. The percentage of signal dots defines the motion coherence. On each trial, three different sequences of dot motion (at the same coherence and direction) are presented in an interleaved fashion, making the effective speed of signal dots 3.83°/s. Signal dots leaving the aperture are redrawn on the opposite side. Across trials, three different levels of motion coherence (10, 30 and 90%) are presented in pseudo-random order. Participants are instructed to fixate their gaze on a central red fixation target (0.3° radius; combination of bulls eye and cross hair [34]) and judge the net motion direction (up vs. down). Random dot kinematograms are presented for 750 milliseconds (ms), followed by a delay of 250 ms, after which two up- and downward pointing arrows appeared left and right of fixation. These arrows indicate the mapping between perceptual choices (“up” vs. “down” motion direction) and motor responses (left vs. right button press). This choice-response mapping is varied randomly from trial to trial, thereby decoupling perceptual choices from motor responses. Participants have three seconds to give a response, and receive auditory feedback about correct (an 880 Hz tone of 150 ms), incorrect (a 200 Hz tone of 150 ms) and timed-out responses (a 200 Hz tone of 300 ms). The next trial starts after a variable inter-trial interval of 0.75 to 1.75 seconds. Participants perform 48 practice trials, after which they complete two main task blocks of 181 trials each. Participants view the stimuli from a distance of 53 cm in a dimly lit room, resting their head on a table-mounted chin rest.

**5.1.3 Reward-driven reach-adaptation task**

We follow the closed loop reinforcement paradigm described in Therrien *et al*. [35] However, our participants are exposed to 40 baseline trials, 270 adaptation trials (Therrien 320 trials) and 50 retention trials (Therrien 100 trials). Furthermore, reward is indicated as a target exploding into fireworks, instead of turning green.

**5.1.4 Paired associate memory task**

To measure episodic memory, we use a two-alternative forced-choice paired associate memory (PAM) task in which unfamiliar faces had to be associated with verbal labels (Figure 2). Facial stimuli with neutral expressions were taken from the modified Facial Expressions for Brain Activation database (https://faces.mpdl.mpg.de/imeji/). The verbal labels are Dutch-language gender-neutral roles (e.g., a profession or hobby), such as “doctor”, “witness”, “judge”, “student” or “swimmer”. The task starts with an encoding phase, followed by a short-term recognition test immediately following the encoding phase and a long-term recognition test after a twenty to thirty-minute delay (filled with other non-memory tasks).

In the encoding phase, participants are instructed to memorize the combination of face and role (intentional encoding). Sixty faces (thirty men) were subsequently presented (2,000 ms each) in the middle of the computer screen with the verbal label presented in lowercase Arial font below the face. For the short-term recognition phase, fifteen of the previously presented faces are shown again, with two verbal labels (i.e., the target label and a distractor that is presented during encoding with a different face) presented at the left and right bottom of the screen (location of target label is randomly assigned). Participants are instructed to indicate the label that was associated with the face during encoding by pressing the left or right arrow key. For the long-term recognition phase, fifteen other previously presented faces are shown, with again the target label and a distractor label (not used as either target or distractor in the short-term recall test) presented at the left and right bottom of the screen. Again, participants have to select the correct labels by pressing the arrow keys. During the recognition tests faces and labels disappear at button press or after five seconds if no button press occurs. Outcome measures are the number of correct responses (hits) in the short and long-term tests (max=15). For measurement two and three, parallel versions were developed with new faces and labels to avoid material-specific learning effects.


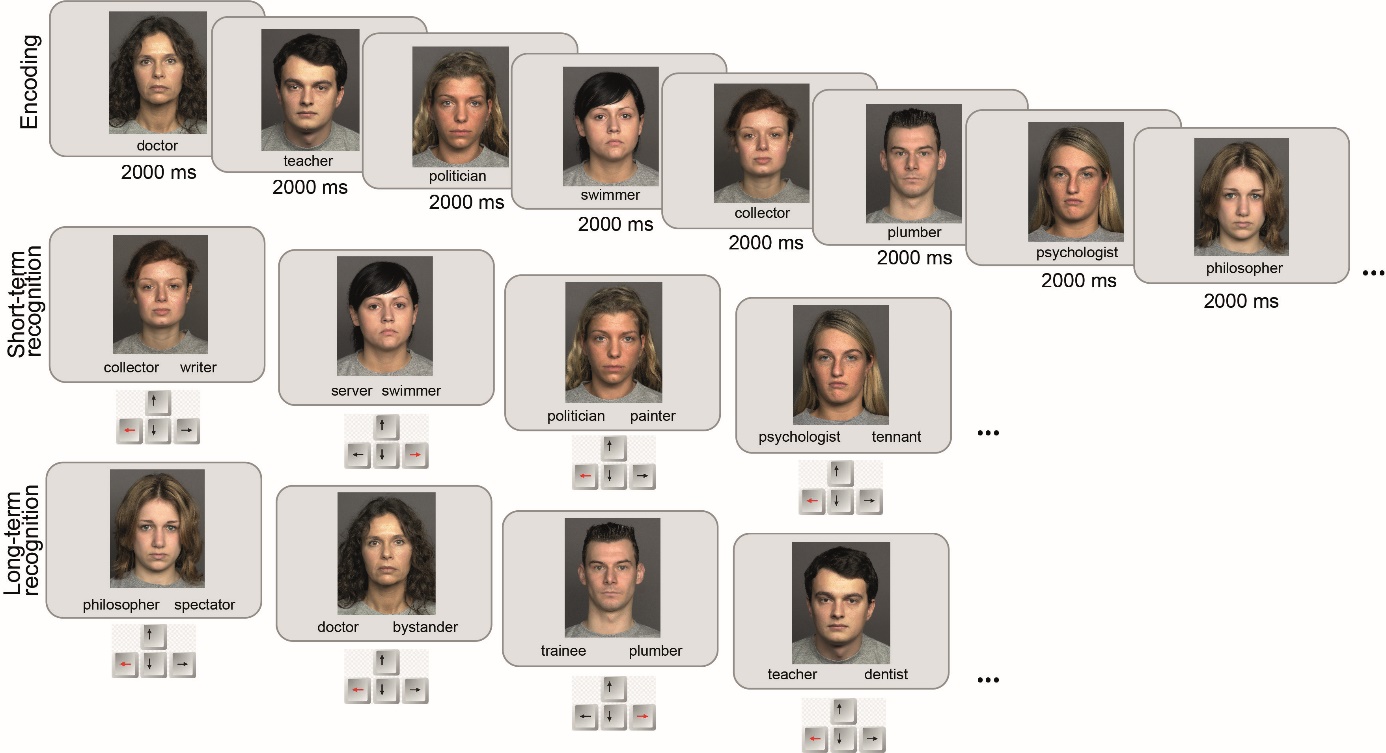


Figure 2. Schematic overview of the paired associate memory task.

**5.1.5 Tower of London**

We use a computerized version of the Tower of London task [36, 37] to assess planning ability. Participants are presented with a starting array of three different colored, same-sized balls and are requested to move the balls one-by-one, with as little moves as possible to a predefined goal array. After being familiarized with the task with an example trial, participants complete three trials that vary in the number of minimum moves (6, 7 and 8). If participants need more moves than a certain maximum (30, 35 and 40), the trial is discontinued, and the next trial presented. Dependent measures are mean number of moves and mean number of time.

**5.2 Affective assessments**

**5.2.1 Contextual fear generalization task**

We use a computerized contextual fear conditioning task to assess fear memory development and generalization. The design of the task capitalized on the idea that contexts serve to disambiguate the meaning of central cues. Given the central role contexts play in the interpretation of a wide variety of stimuli surrounding us, it is believed that alterations in contextual processing may pose an important vulnerability for the development of anxiety [38], contributing to exacerbated fear generalization. The paradigm is based on previous studies using a similar design [39, 40]. For the threat condition, presentation of the conditioned stimulus (CS) in one specific context (background picture) predicts the occurrence of the unconditioned stimulus (US; shock). In another context, the same CS is not followed by the US (safe condition). During the unreinforced generalization phase, these two conditions are alternated with presentation of the CS in a new, and thereby ambiguous, context, enabling us to assess generalization across context (generalization condition). Modulation of defensive responses (startle eye-blink) by threat-imminence is assessed during CS presentation relative to inter-trial intervals (ITI). Standard differential conditioning could be assessed by comparing startle reactions during the CS in threat versus safe context.

In detail, a total of three different background images (i.e., contexts) is used, all depicting offices. Assignment of these images to either the threat, safe, or generalization condition is counterbalanced across participants. In the experiment only one CS is used, a picture of a standing man in a casual-chic office suit. Any given trial during the experiment consists of the same build-up, starting with an ITI that takes a variable 10 ± 1 seconds. Then the context appears together with the CS for five seconds and the startle probe (50 ms – white noise - +/- 105dB) is always presented at 4.5 seconds. When applicable, the shock is presented at CS offset. Upon disappearance of the CS the context is visible for its remaining duration.

The procedure consists of several phases. Before the start of the task, electric shocks are set to an unpleasant but not painful level with a standardized work-up procedure which consists of five electrical shocks of variable intensities that are rated on their unpleasantness (adapted from Klumpers *et al*. [41]). Next, participants are instructed that they will see foreground and background pictures, and that their task is to predict when a shock will occur. The task starts out with a probe habituation phase to the startle probes (noise alone, NA), in order to reduce possible initial reactivity of blink responding. In total four startle probes are presented, with an inter-probe interval of nine, eleven, or thirteen seconds. Then, the context habituation phase commenced, which was designed to familiarize participants with all contexts (presented without the CS) and to exclude the possibility that conditioned responses to the generalization context presented later in the test phase could be explained by mere novelty effects. Also, one NA probe is presented. Afterwards, the actual acquisition phase commences. During this phase, a total of twelve threat trials and twelve safe trials is presented. The CS in the threat context is reinforced with shock ten times, the two unreinforced trials are fixed to the third and the seventh trial to keep learning rates comparable across participants. After sixteen trials (i.e., at two thirds of the phase) another break was implemented, followed by the remainder of acquisition. During the ensuing generalization test, again four threat and four safe trails are presented, intermixed with four generalization trials. None of these trials are reinforced. During the generalization trials, the same CS is presented but against a new background context. As such, the interpretation of the CS-context combination in terms of shock reinforcement is ambiguous. The generalization test phase always starts with the generalization trial, followed by a safe and threat trial, in order to obtain a clean primary response on generalization that will be comparable across participants.

Presentation of all the stimuli in the experiment is semi-randomized. For acquisition, safe and threat trials are shuffled in blocks of two and in later phases the safe, threat and generalization trials are shuffled in blocks of three. Consequently, no more than two of the same trials will follow-up on each other. Startle probes are presented during all CS images. A context probe is presented randomly every two trials of each threat type. ITI probes are presented randomly every four trials of each threat type. In summary, every four trials (or six during the test phases) two safe and two threat trials, one context safe probe and one context threat probe, and one ITI probe is presented.

At the end of the task, we assess subjective aversiveness of the shocks and startle probes and verify contingency awareness by asking participants to identify the combination of CS and context that predicted shock during the first part of the experiment. The latter involves a forced choice procedure showing all three contexts. Finally, avoidance tendencies for each of the contexts is assessed by asking participants to indicate whether they will choose to again see each context/cs combination. To stimulate approach/avoidance conflicts and model mixed outcome prospects under which maladaptive avoidance occurs [42, 43], we ask participants whether they will view each picture if they will receive a small (0.10 euro) or larger (0.50 euro) monetary reward.

- - 1. **Emotion regulation task**

To measure the capacity for self-regulation of emotion toward a more positive state, we use an adapted version of the situation-focused reappraisal task [44]. Participants see either a positive, negative, or a neutral picture, which they either have to reappraise or view. To increase a more positive state, participants are instructed to either focus on the positive aspects of the situation or to imagine a (more) positive outcome. Success of emotion regulation is assessed using modification of the eye-blink startle reflex, and using self-report of subjective emotional experience.

The task starts with psychoeducation on emotion regulation and a practice run of the task. Each trial begins with a one-second presentation of a strategy cue (‘reinterpret positively’ or ‘view’) followed by a six-second presentation of a picture. A 100-dB, 50-ms burst of white noise is used as a startle probe. The probe is administered through headphones and is presented randomly between three and five seconds posttrial onset on each trial. Picture offset is followed by a self-assessment manikin scale of four seconds, on which participants indicate how happy they felt while watching the picture on a scale from zero (very happy) to nine (very unhappy) [45]. Each trial ends with the presentation of a black fixation cross (for one second). The task consists of thirty trials: six ‘reappraise’ and six ‘view’ trials for both negative and positive pictures; six ‘view’ trials for neutral pictures.

For the three repeated measurements, we created a different set of pictures based on the EmoPicS [46] and IAPS [45] picture sets. We excluded erotic pictures and pictures containing nonsocial situations. Pictures with high valence (> 6.5) and high arousal are used as positive pictures, pictures with low valence (< 3.5) and high arousal (> 4) are used as negative pictures. Pictures with moderate valence (4.5 – 5.5) and low arousal (< 2.5) are used as neutral pictures. No pictures are used more than once.

The startle response to the probe is measured via electromyography (EMG) of the orbicularis oculi muscle using Ag/AgCl electrodes.

- - 1. **Self-referent encoding task**

A computerized version of the self-referent encoding task (SRET; [47, 48]) is used at each assessment (i.e. three times) to assess memory bias. The SRET consists of an encoding phase and a recall phase. During the encoding phase, twelve positive and twelve negative self-descriptive adjectives are individually presented on a computer screen. An exclamation mark precedes the presentation of the next word. No more than two words of the same valence are presented sequentially. The participants are instructed to press the ‘j’ key (‘ja’ being Dutch for ‘yes’) on the keyboard if the word described them, and the ‘n’ key if the word is not self-descriptive. Per study visit, a unique set of twelve positive and twelve negative words is selected form the in total 72-item word list, hence each participant sees each word only once. The order of presentation (across and within test sessions) differs between participants. After a brief distraction task (the paired associate memory task; Chapter 5.1.4), a free recall phase follows in which participants are asked to type in (within 3 minutes) all words they remember from the encoding phase within three minutes. Guessing is stimulated.

Spelling errors are permitted. A negative memory bias index is calculated by dividing the number of negative words endorsed as self-descriptive and subsequently correctly recalled by the total number of words endorsed and recalled [49].

The SRET is generally used as a one-time measure of memory bias. Due to our longitudinal design, we adapted the SRET for repeated testing. To have enough words available for three test sessions, word sets from different memory bias studies [50-52] were combined. The other adaptation was the overt instructions with regard to the purpose of the task. During the encoding phase, participants are informed that a recall phase will follow. Most studies implement an incidental learning phase, but directed instructions have been previously used with success [51].

- - 1. **Stimulus-response compatibility task**

The stimulus-response compatibility (SRC) task is used to measure an alcohol approach bias. The design of this task is based on a similar task used in previous studies [53, 54]. The task includes two picture categories, i.e., alcohol pictures and soda pictures, displaying human interaction while drinking either alcoholic or soda beverages respectively. Twenty pictures are included for each category. Pictures from both categories are matched on the number of people present, the gender of the people in the picture, as well as the social setting (e.g., dining table, bar, or couch). People displayed on the pictures have the same age range as our participant sample (30-39 years old). Beverages displayed on the picture include different types of beers and wines as well as different types of soda consumptions.

The task starts with sixteen practice trials. Each trial starts with a fixation-cross displayed for 1,000 ms, followed by a 2,000 ms presentation of the picture presented with a manikin randomly positioned above or below the picture. Responses are given by pressing the “up” and “down” keyboard buttons, moving the manikin in that direction. After incorrect responses, a red cross is presented on the screen for 2,000 ms. During the “approach alcohol” block participants are instructed to approach the pictures that depict alcohol and to avoid the pictures depicting soda. During the “avoid alcohol” block the instructions are reversed. All pictures are presented once within a block, resulting in forty trials per block (eigthy trials total). The order of blocks (“approach alcohol” versus “avoid alcohol”) is counterbalanced. For both alcohol and soda pictures, a separate approach bias score is calculated by subtracting the mean reaction time in the avoid block from that in the approach block resulting in a separate alcohol and soda approach bias scores.

**5.3 Behavioral measurements**

**5.3.1 Columbia card task**

We use the hot and cold versions of the Columbia card task (CCT) as described in Figner and Weber [55]. In the CCT, each game round starts with 32 cards shown from the back. On top of the screen, information is presented about the loss amount, the gain amount, and the number of loss cards in the current game round [56]. In addition, the current round score is presented (each new game round starts with a score of zero). In the hot CCT, when a participant clicks on a card, it is turned over, revealing whether it is a gain card or a loss card. Turning over a gain card adds the stated gain amount to the current total score. Turning over a loss card ends the current game round and subtracts the stated loss amount from the current total score. Participants can turn over additional cards until they decide to stop (because it is getting too risky) or until they turn over a loss card. Across the 24 game rounds, the number of loss cards (1 or 3 out of the 32 cards), the gain amount (10 or 30 cents per gain card), and the loss amount (250 or 750) are varied according to a full factorial design with each of the two times two times two combinations presented three times (once in each block eight trials). The cold CCT is identical to the hot CCT, except that participants do not make decisions in a stepwise manner by clicking on one card after the other, but instead, they indicate how many cards they want to turn over in a game round by clicking one of the buttons labeled 0 to 32 on top of the screen. In the cold CCT, no feedback about the outcomes of their decisions is given until all 24 game rounds have been finished. After the CCT, a series of questionnaire items are presented, including questions about how participants made their decisions, and including the need-for-arousal scale [56]. The main variable of interest in the CCT is the number of cards turned over per game round as an indicator of risk taking, and how the risk-taking levels differ as a function of the gain amount, loss amount, and loss probability (i.e., number of loss cards).

**5.3.2 Food auction task**

**Auction.** Participants are asked to bid on 24 experimental food items (six savory processed snacks; six sweet processed snacks; six fruit snacks; six vegetable snacks) using the Becker-DeGroot-Marschak (BDM) willingness to pay (WTP) procedure [57] as implemented by Veling *et al*. [58]. Four filler items are also included. Participants are given two euro and are informed they will be able to purchase one of the food items they have bid on for actual consumption at the end of the experiment. Participants indicate their bids by using a zero to two euro slider at the bottom of the screen. The entirety of the auction was self-paced, and the next item only appears after a successful bid. At the end of the experiment, the computer program picks one filler item auction trial and generates a random bid. If the computer's bid is less than the bid of the participant, then the participant purchases the snack for the computer's bid.

**Food choice.** In the subsequent choice task (adapted from Schonberg *et al*. [59]), participants are presented with two food items left and right of a central fixation cross. They are asked to choose one of the items (by pressing either the U (left) or I (right) keyboard button) within 2,500 ms of stimulus onset. After choice, a yellow frame appears around the item for 1,000 ms as confirmation. The intertrial interval varied from 1 to 1.5 s in steps of 100 ms. Participants are informed that one of the trials will be selected and they will receive the snack they chose on the selected trial for actual consumption. If participants do not respond within the time window a text would appear on the screen prompting them to choose faster. Those trials will be repeated later.

The task consists of 96 trials with experimental items. Participants receive six choices between two items within one of four different categories (savory; sweet; fruit; vegetable). Choice pairs are constructed based on the ranking in the auction (one vs four; two vs five; three vs six) and presented two times with position counterbalanced (total of 24 choice trials; validation trials). Participants receive a total of 72 choices between a food item from one category and similarly ranked food item (auction) of another category (one vs one; two vs two; three vs three; four vs four; five vs five; six vs six; presented two times with position counterbalanced). Trial presentation is random. Two filler choices are added with the filler food items (a fruit vs a sweet snack; a vegetable vs a savory snack) and one of these is randomly selected and the choice a participant made on that trial is rewarded.

1. **Lab visit: sensory tests**

**6.1 Vision**

Firstly, participants perform the Pelli Robson test. In this test the contrast sensitivity is measured by identifying letters on a card. These are black letters printed on a white background. The letters are printed with decreasing contrast. The score equals the number of correctly read letters. The letter series are grouped in a series of three (triplet), with two series per line. Letters in the same series have the same contrast. The score equals the number of correctly read letters. Secondly, participants perform the Early Treatment Diabetic Retinopathy Study (ETDRS), which is a test to measure visual acuity. Thirdly, participants perform the Ishihara charts test. This test indicates whether someone is color blind and to what extent. The test consists of eleven colored pictures. Each picture consists of a circle of dots. The collection of dots form a number that is visible to people with normal color vision and is invisible or difficult to see for people with a red-green color blindness.

**6.2 Hearing**

Participants’ hearing is measured with a hearing check screener device (Draft, Siemens, Germany)

This device is used to quickly and easily test hearing by playing back acoustic signals with a specific volume and in a specific order.

1. **Post-visit online assessments**

**7.1 Decision-making**

In the online decision-making experiments, the participants make allocation decisions in (risky) economic environments. Some tasks consider hypothetical decisions while in other tasks, the participants’ decisions lead to monetary consequences depending on their performance in the tasks. While some tasks are individual decision-making tasks with monetary consequences for the participant only, other tasks also have monetary consequences for other participants. In such tasks, the program randomly matches several participants in pairs or groups. Participants are not matched twice throughout the task (e.g., “Note that the participant who is matched with you in the following task, will not be matched with you in any other part of the experiment”). Prior to each task, a message is displayed that indicates whether a task has a chance to be paid out or not. Only a small fraction of tasks will be chosen for payment which is unknown to the participants (Chapter 7.1.7).

**7.1.1 Trust game**

The trust game has a long history in experimental economics with over 150 publications. The current implementation is in line with Hergueux and Jacquemet [60] who apply the setting to an online environment. Participants A and B receive a monetary endowment *E.* Participant A transfers an amount *X ≤ E* to participant B. This amount is tripled and added to the endowment of participant B. Participant B is not willing to lose money by sending *Y > 0*. However, the social optimum is *X = E* as *2E* can be achieved in addition. Each participant takes the role of A and B in the first and sixth game in a chain of task. Measurement: The amount sent *(X)* measures ‘trust’ while the amount returned measures ‘trustworthiness’ (usually the return ratio *Y / X* or the return of investment *(Y – X) / X*. For the implementation of participant B’s decision, we apply the strategy elicitation method which requests participant B to state the amount to return for every possible amount transferred from participant A. Incentives: Participant A earns *E – X + Y* and participant B earns *E + 3X – Y* with the endowment *E* being five euro.

**7.1.2 Equality equivalence test**

The Equality-equivalence test (EET) is a method for eliciting social preferences in a two-person context (income self vs income other) introduced by Kerschbamer [61] and implemented in an online environment in Kerschbamer and Müller [62]. The measure provides a distinction between nine archetypes of social preferences (spiteful, kick-down, equality averse, envious, selfish, kiss-up, inequality averse, maximin, altruistic). It gives rise to a simple technique that discriminates between the nine archetypes according to core features of preferences rather than properties of specific modeling variants or functional forms. The basic version of the EET exposes participants to two choice lists, one located in the domain of disadvantageous inequality and the other located in the domain of advantageous inequality. The switching points chosen in the choice list construct an index to measure the pro-sociality of the participant separately for each domain. Both scores have in common that a positive (negative) score stands for benevolence (malevolence) and that a higher score means ‘more benevolent’. Kerschbamer explains the measurement and the theoretical background in detail [61]. The online implementation in the HBS follows Kerschbamer and Müller [62].

**7.1.3 Higher-order risk preference task**

In the literature (among other disciplines) on economic or financial decision-making under risk, the focus has generally been on the impact of first order risk attitudes, i.e. whether people are risk averse, risk-neutral, or risk-seeking. However, many decisions also depend on higher-order risk attitudes. This task measures first-order risk preferences (risk aversion), second-order risk preferences (prudence), and third-order risk preferences (temperance). Prudence implies precautionary saving: greater savings in response to an increase in background risk. Temperance implies less risky investment in the presence of greater background risk. Thus, in the presence of future income risk, participants who are prudent and temperate save more, but invest fewer of these savings in risky assets, than those who are neither prudent nor temperate [63].

Measures of prudence and temperance are model-free, in the sense that they do not require auxiliary assumptions about how probabilities enter decisions, such as the expected utility hypothesis. The implementation of the measurement in an online environment is explained in Noussair *et al*. [63]. Participants make lottery choices between two (compound) lotteries. A risk-averse participant typically picks the safe outcome. To measure prudence, participants choose between lottery L in which a zero-mean risk occurs in the high wealth state and lottery R in which a zero-mean risk occurs in the low wealth state. A prudent participant typically picks lottery L over lottery R, accepting additional risk in the state of high wealth. To measure temperance, participants choose between lottery A in which a zero-mean risk occurs in each of two similar wealth states and lottery B in which two zero-mean risks occur in one of two similar wealth states. A temperate participant typically picks lottery A over lottery B to disaggregate the two risks. The participants make fifteen choices in total to measure the three different orders (five choices per order). Each participant’s measure of risk aversion is the number of safe choices (zero to five), each participant’s measure of prudence is the number of prudent choices (zero to five), and each participant’s measure of temperance is the number of temperate choices (zero to five). In this task, we do not apply monetary incentives since Noussair *et al*. [63] found no difference between real and hypothetical decisions.

**7.1.4 Time preferences**

The implementation to measure time preferences (or patience) follows Falk *et al*. [64]. The participants face a series of five interdependent hypothetical binary choices between immediate and delayed financial rewards (termed ‘staircase’ procedure): “Suppose you were given a choice between receiving a payment today or a payment in one year. We will now provide five situations. The payment today is the same in each of these situations. The payment in one year is different in every situation. For each of these situations, we would like to know whether you want the payment today or in one year. Please consider the following: Would you rather receive 100 euro today or $x$ euro in one year?” Falk *et al*. provide the sequence of questions in their appendix [64]. The participant receives a patient score that can range from 1 (impatient) to 32 (very patient). The former says that the participant would prefer 100 euro today than 215 euro in twelve months, while the latter says that the participant would prefer 103 euro in twelve months than 100 euro today. The HBS makes use of the hypothetical setup as immediate payments are not possible for this online experiment.

**7.1.5 Public good game**

The public good game considers a social dilemma situation to measure altruism and conditional reciprocity in a group. The implementation in HBS is in line with Hergueux and Jacquemet [60] who apply the setting to an online environment. Four participants form a group endowed with ten euro *(E)* per person. In one decision (unconditional), each participant decides on how much of the endowment to contribute to a public good *(C_i_ ≤ E)*. The amount contributed is subsequently multiplied by 1.6 and equally shared among the participants in the group. As the participant can keep the remaining amount, the payment equals 10 - *C_i_ + 1.6 x ∑_j_C_j_* for each participant. This scenario is a social dilemma as each euro contributed yields a return of 0.4 euro while the kept amount yields one euro. Hence, free riding and hoping for others to invest is the selfish solution, even though contributing the entire amount *Ci =* E yields the social optimum. In a second decision (conditional), each participant provides their intended contribution *(Ci(∑_j≠i_C_j_)* for each possible average contribution of the three members in the group (ranging from 0 to 5 euro with 0.50 euro increments). One of the two decisions is randomly drawn to be paid out. The unconditional contribution measures the ‘unconditional altruism’, that is, higher contributions relate to a higher willingness to cooperate. The conditional contribution measures ‘conditional altruism’, which quantifies how the participants react to the decisions of others.

**7.1.6 Ambiguity**

Using a matching probability approach, we measure ambiguity aversion via a-sensitivity (ambiguity –generated likelihood insensitivity). A-insensitivity reinforces risk seeking for long shots; as a result, people overweigh extreme events, both favorable and unfavorable. Such individuals are oversensitive to signals affecting the tails of distributions. Similarly, it undervalues preventive measures that reduce uncertainty without eliminating it, while overvaluing the complete elimination of uncertainty.

The implementation of the measurement in an online environment is explained in Dimmock *et al*. [65]. The participants choose between two urns, urn K (Known) and urn U (Unknown). Each urn contains yellow and purple balls. In urn K, the participants learn the probability distribution (e.g., ten yellow and then purple balls). In urn U, the probability distribution is unknown (U, unknown distribution of yellow and purple balls). One color is the winning color, such that drawing the winning color from the chosen urn earns a fixed monetary amount. In the example, the objective probability of drawing the winning color in both urns is 50%. When participants assign a lower subjective probability to draw a yellow ball from urn U than from urn K, they are ambiguity averse. When they assign a similar subjective probability, they are ambiguity neutral. When they assign a higher subjective probability, they are ambiguity seeking. The measurement aims to elicit the subjective probabilities assigned to the urn with unknown probabilities using a bisection procedure. Over a sequence of choices, the probabilities are automatically adjusted for urn K until the participant indicates to be indifferent between urn K and urn U. For example, when a participant is indifferent between making the draw from urn K with 40% chance for the winning color and urn U still with unknown probability, then the participant assigns a 40% winning chance to urn U as well. The more ambiguity averse a participant is, the lower this ‘matching probability’. In a similar procedure, the urns contain ten balls with up to ten different colors. In two different implementations there exists either one or nine winning colors. As a result, we get three matching probabilities for each participant: m(0.5), m(0.1), and m(0.9) with the probabilities 0.5, 0.1, and 0.9 respectively. More detailed information about the implementation can be found in Dimmock *et al*. [65], in particular in the supplementary material.

These measures assign global ambiguity attitude indexes. A fit $m\left( p \right)=c+sp$ measures each participants’ index of insensitivity ($a=1-s$) and index of ambiguity aversion $(b=1-s-2c)$. The first index measures the elevation of a probability weighting function and the latter the curvature of that weighting function. The local ambiguity aversion measures are just $AA_{p}=p-m(p)$. If $AA_{p}>0$, the participants are ambiguity averse; if $AA_{p}< 0$, the participants are ambiguity seeking (often for nine winning color cases).

Dimmock *et al*. [65] conclude that “measuring ambiguity attitudes for nonacademic subjects, hypothetical choice does not work well, unfortunately.”(p.1369). Hence, the participants earn the outcome of the lottery.

**7.1.7 Incentives**

To determine the payment, we group the participants in clusters of eight participants. Within each cluster, we assign an index number 1-8 for each participant to enable matching between participants within the cluster in unique groups and pairs (public good game: first group participants 1-4, second group 5-8; trust game: pairs 1&5, 2&6, 3&7, 4&8; equality equivalence test: pairs 1&6, 2&7, 3&8, 4&5).

**7.2 Narrative reading**

Participants read three literature stories, written by three different authors (Table 2). The stories are all published stories, written by acclaimed authors. Each story is presented in the language it is originally written in (Dutch). Stories are not shortened or otherwise alternated. The topics of the stories are diverse. Each story involves interactions between two characters, and little change of place, as is typical in short literary narratives.

Table 2: Details of published Dutch stories read by the HBS participants.

|  | **Title** | **Author** | **Year of publication** | **Number of words** |
| --- | --- | --- | --- | --- |
| Story 1 | De invaller (‘The substitute’) | René Appel | 2003 | 745 |
| Story 2 | Motherhood (‘Motherhood’) | Sanneke van Hassel | 2012 | 1467 |
| Story 3 | De tekening (‘The drawing’) | Thomas Rosenboom | 2006 | 1287 |

Participants are instructed to read each story as they normally would. The instructions mention that they do not have to pay attention to anything in particular. It is also mentioned that participants will receive several questions about their understanding and reading experience, after reading of the story.

Each participant reads each narrative (within-subjects design). Each narrative is read on a different assessment.

After reading each story, participants answer two multiple choice questions (four answer alternatives) about content of the stories. The questions are about literal content (no interpretation questions), and are mainly included to check whether participants have actually read the story. Next, participants choose one to three words (out of fourteen alternatives) that they think best described the topic of the story. This question is used to investigate how similar participants are in detecting topics of a literary narrative. The response options are fourteen nouns or adjectives. They are the same for all three narratives.

Next, participants fill out the Story World Absorption questionnaire (SWAS) [66]. The SWAS is a validated and often used questionnaire for testing absorption (sometimes called immersion) into a narrative world. We use the original version of the questionnaire as published by Kuijpers *et al*. [66]. The questionnaire consists of eighteen items that together measure overall absorption and can be both conceptually as well as psychometrically be divided into four subscales: emotional engagement, mental imagery, transportation, attention.

To measure appreciation (‘liking’) of the narratives, participants answer a single question measuring overall appreciation (‘How much did you like this story?’, 7-point Likert scale), and the Multivarariate Appreciation Questionnaire (MAQ). This questionnaire has been used before by the authors [67, 68] and was inspired by the work of Knoop *et al*. [69]. In the MAQ, participants indicate how much they believe each of fourteen adjectives is applicable to the story. Examples are ‘sad’, ‘interesting’, ‘moving’, ‘captivating’, ‘funny’. Participants indicate on a 5-point Likert scale how much they find the adjective to be applicable to the narrative they just read. A psychometric analysis shows this test to have high internal validity and to capture at least three different aspects of aesthetic appreciation of narratives (Mak *et al*., under review).

Next, participants indicate how much effort they exerted during reading. They scored on a 7-point Likert scale how sloppy / effortful they read the story compared to their normal reading. Finally, we ask if participants read the story before, and if they think they know who the writer is. If so, they are asked to fill in the writer’s name.

After reading the third story (at the final session in which narrative measurement are taken), participants fill out a seven items questionnaire about their reading habits. This questionnaire asks about the frequency of reading (both fiction and non-fiction), as well as for genre preferences. The questionnaire was developed and used before by the authors [67, 70].

- 1. **Solidarity**

In a vignette study, participants are presented with vignettes (e.g., a short text) in which hypothetical situations are described. Participants are asked to imagine the hypothetical situation and to respond to it. Vignettes typically include a number of variables that are of interest for the study (i.e., the theme of the issue described in the vignette, and the identity of the person/group in the vignette). These variables were varied randomly among the vignettes, and the vignettes are distributed randomly among the participants. This enables us to obtain insight into participants’ responses in systematically varied (social) situations [71, 72], as well as to assess whether the responses to these situations are dependent on participant characteristics. To fully capitalize on the longitudinal nature of the study, participants are presented with a set of four vignettes each at four different measurement points. This longitudinal vignette design moreover allows to assess changes in the responses to hypothetical situations within persons over time, as well as the effect of external factors on these within-person changes.

In the vignette study we assess people’s willingness to support other people in society for four different themes: work, medical ethics, preventive medicine, and politics. Within each of these themes, three characteristics are varied: 1) the identity of the other person/group in the vignette, which is either specified as an (ethnic or religious) outgroup or not specified; 2) the culpability of this person/group in the vignette, which is either specified as something the person/group could have done to prevent the situation, or no culpability mentioned; and 3) the kind of support the participant is willing to offer, reflecting the most common types of public support typically called for. The first two characteristics are included as vignette variables, while the third characteristic is incorporated in the answer categories, where four options are given with different levels of investment required: no support (no investment), signing a petition (low investment), donating money (low or high investment, depending on economic position), coming along to a meeting (high investment). This leads to a universe of four vignettes per theme, amounting to a total of sixteen vignettes (four themes × two (yes/no culpability × two (in/outgroup))). Table 3 shows the elaborated vignettes for each of the four themes and the four responses participants were asked to choose from after each vignette. The exact formulation of the two vignette variables and the four response options vary slightly per theme, such as to correspond to the nature of the issue presented in the theme. This, however, does not necessarily affect comparability between themes, given that the core meaning of the three vignette characteristics is preserved.

We present each participant with four vignettes per measurement point, making sure that each specific combination of vignette characteristics is presented only once. Since we have four measurement points, this means that each participant responds to all sixteen vignettes. Table 3 shows an example of the distribution of the vignettes over four measuring points for one participant. As the table shows, randomization is restricted in the sense that each measuring point should include all four vignette themes as well as all four options of the vignette characteristics. This was done to facilitate longitudinal analysis, as it allows to isolate the effect of vignette characteristics on one hand from potential effects of external factors that happen in between measurement points on the other.

Table 3. Example distribution of vignettes for a participant

|  | **Measurement point 1** | **Measurement point 2** | **Measurement point 3** | **Measurement point 4** |
| --- | --- | --- | --- | --- |
| **Vignette characteristics** |  |  |  |  |
| Culpability |  |  |  |  |
| *Yes* | W (no outgroup) | ME (no outgroup) | PM (no outgroup) | P (no outgroup) |
| *No* | ME (with outgroup) | PM (with outgroup) | P (with outgroup) | W (with outgroup) |
| Identity |  |  |  |  |
| *Ingroup* | PV (with ‘yes’) | P (with ‘no’) | W (with ‘no’) | ME (with ‘no’) |
| *Outgroup* | P (with ‘no’) | W (with ‘yes’) | ME (with ‘yes’) | PM (with ‘yes’) |

*Themes: W=work; ME=medical ethics, PM=preventive medicine; and P=politics*

**8. Individual differences in language skills test battery**

The Individual Differences in Language Skills (IDLaS) test battery is a set of 31 behavioral experiments recently developed by Hintz, McQueen, and Meyer (in preparation). IDLaS assesses Dutch individuals’ linguistic knowledge, general cognitive skills, and linguistic processing skills.

The assessment of IDLaS is divided into four sessions of about one hour each (Table 4 for an overview). Sessions can be completed between 6 am and 10 pm (CET), within a period of two weeks from the start of the first session. Participants complete maximally one session per day. As is common practice in individual differences research, the order of tests (see Table 4) and the order of trials within each test is the same for each participant to minimize potential influences of the test procedure on participants’ performance.

The tests were implemented in Frinex (FRamework for INteractive EXperiments, a programming environment developed by the technical group at the Max Planck Institute for Psycholinguistics) and are conducted via the internet. Specifically, each test was implemented as a web application and run in Electron, a platform-independent application simulating Google Chrome on the participant’s computer. The Electron application takes up the computer’s full screen space and ensures that each participant runs the tests in the same browser. For tests involving the playback or recording of sound, participants use headphones and an integrated or external microphone, respectively.

The materials, design, and procedure of the 31 individual tests are described below. In general, picture and text stimuli (font Calabri 17, font color RGB 0, 102, 102 (green/blue) are presented against a white background. Moreover, if not stated otherwise, auditory stimuli and speech recordings have a sampling rate of 44100 Hz, 16-bit resolution. We used the Dutch Subtlex [73] and prevalence [74] databases to retrieve the stimulus words’ frequency (operationalized as Zipfian frequency, ZipfF) and prevalence, respectively.

Note that the order of the test descriptions below was not the order in which they are administered (see Table 4 for an overview). We first describe the test session-initial questions about well-being, and then the audio test procedure. Then, we describe all tests belonging to the ‘Linguistic experience’ domain, followed by descriptions of all tests belonging to the ‘General cognitive skills’ domain and, finally, we describe all ‘Linguistic processing skills’ tests.

The raw data, including accuracy scores, reaction times and speech recordings, obtained from the 31 tests will be made available to interested researchers. Note that speech recordings will need to be transcribed and/or annotated before conducting further analyses. Similarly, data from tests that involve reaction times need to be pre-processed (treatment of extreme values and outliers, Table 4 for an overview). For these tests, we describe a performance indicator that can be obtained after transcription/annotation or pre-processing. For accuracy-based tests, we provide both the raw (i.e., item-level) data as well as participants’ final scores.

**Well-being questions, audio tests**

Participants begin the first test session by filling out a questionnaire assessing language background and musical training. After completion of the questionnaire, participants respond to three questions assessing how they slept the night before the test day, how their mood is on the test day, and how they would rate their health, each by selecting the appropriate option from a drop-down menu (options: very good, good, average, bad, very bad). Following the questions about their well-being, in sessions two, three and four, participants complete a test assessing whether their microphone and audio settings are set properly, that is, whether playback and recording of speech work. To that end, participants first name four Dutch words. The recordings of these four words are then played back to them in random order and they indicate via mouse click which of the words they heard. This procedure is repeated until the participant reaches 100% accuracy, and participants are asked to adjust settings if necessary.

**Linguistic experience tests**

**8.1 Stairs4Words**

We use Stairs4Words, a newly developed adaptive test, to assess receptive vocabulary size. On each trial of the test, participants see a word or a non-word foil and indicate whether or not they know the item. They are told that ‘knowing a word’ implies having heard or read the word before, and having at least a general idea of its meaning. Participants are informed that some of the stimuli are made-up non-words and that they thus have to carefully consider whether they know a word or not.

The selection of words and non-words was based on prevalence norms (i.e., norms specifying which proportions of a sample knew the words) provided by Keuleers *et al* [74]. The database by Keuleers *et al*. contains prevalence measures for approximately 54,000 Dutch words. These measures approximate to what extent a given word is known to the whole population. The words were sorted by prevalence and then divided into 53 bands of 1,000 words and one band of 582 words. We selected forty words from each band, amounting to an item bank of 2,160 words covering the entire prevalence range (from words known by > 99% of the sample to words known by < 1% of the sample) in 54 bands. Words were selected to have similar prevalence values across males and females, different age groups, and Belgian and Dutch speakers. Plural forms, past tense forms of verbs, first person singular forms of verbs and English loanwords were not selected. We devised two parallel versions of the test that were identical in structure and task but used different words in each band. That is, the forty words selected from each band were evenly distributed across both versions of the test. The resulting lists were sorted by band and the words within a band were sorted by prevalence, starting with the most prevalent word. The order on the two lists was fixed such that when sampling words from a given band for presentation in the test, words are always sampled in the same order for each participant.

The test begins with the presentation of an easy word, sampled from band 20 (representing words known by > 95% of the norming sample), shown in the center of the screen. Participants respond by mouse-clicking on an ‘I know the word’ (right-hand side) or ‘I don’t know the word’ (left-hand side) button, respectively, positioned under the target word. In case of a positive response (‘I know the word’), the next word item is harder than the previous one (it came from the next higher prevalence band). In case of a negative response (I don’t know the word), participants are presented with another word from the same difficulty band. Non-words are interspersed with the existing words at a ratio of 6:2 (two non-words on six existing words). As for the words, non-words are sampled in the same order from the item bank. Two non-word trials are never presented in succession. Non-words require a negative response (I don’t know the word); a correct rejection also moves the participant to a more difficult band.

This process continues until participants make two successive ‘mistakes’: either (a) indicating twice in a row that they do not know an existing word or (b) indicating that they do not know an existing word and indicating they knew a non-existing word. This ‘fast-track’ part of the test is followed by a ‘fine-tuning’ part using a staircase procedure, as is often used in psychometric testing [75]. The crucial difference to the fast-track, not noticeable to the participants, is that in order to move to a more difficult band of items, they need to respond correctly to four items (three words and a foil) in a row. A single error moves them to a lower band.

The test is untimed and ends after an ‘incorrect’ response (no to an existing word or yes to a non-existing word) occurring after two consecutive oscillations between two adjacent bands. The test also ends when all test words from a given band have been used up such that no test word was available for presentation. In the latter case, the number of the band most frequently visited during the fine-tuning part is the end score. In case the test is terminated after two consecutive oscillations between adjacent bands, the lower number of the two bands is the end score. Participants carry out both test versions and receive two scores. Participant’s performance indicator is the average of both scores.

**8.2 Peabody picture vocabulary test**

We use a digitized version of the Dutch Peabody picture vocabulary test (PPVT [76, 77]) as a second test for assessing receptive vocabulary size. On each trial, participants preview four numbered line drawings on their screen and then hear the probe word. They have to indicate which of the four pictures best corresponded to the meaning of the spoken word by clicking on it. Participants can listen to the probe word as often as they want, but have to listen to it at least once before a response is recorded. The test has seventeen blocks, each consisting of twelve items of roughly the same difficulty. The test starts at block thirteen. Based on their performance (four or fewer errors in block thirteen), participants’ next block is either more difficult (block fourteen) or easier (block twelve) than the entry block. The test terminates when more than eight errors are made within a block that was not the starting block or when a participant reaches the last item of the test. The participant’s performance indicator is the difference between the item number of the last test word and the number of errors participants made.

**8.3 Idiom recognition test**

Participants’ knowledge of Dutch idiomatic expressions is tested using an idiom recognition test. On each trial, the participants are presented with a Dutch idiom, such as ‘tussen de regels door lezen’ (to read between the lines) and a set of four candidate meanings. Participants are presented with an idiom at the top of the screen and four meaning candidates are shown underneath in four quadrants of the screen. The position of the target meaning varies across trials. Both the idiom and the four candidate meanings can be listened to (by mouse-clicking on a loudspeaker icon) to account for the possibility that some idioms are predominantly encountered in the spoken modality and to reduce the influence of reading skill on recognition performance. The performance indicator is the proportion of correctly recognized idioms.

**8.4 Spelling test**

We use a recently designed test to assess language users’ spelling skills. The test consists of a list of sixty Dutch words whose spelling adult language users often find difficult. These concern for example the correct use of diaeresis (i.e., ‘bacteriën’, bacteria), the use of double consonants in plural forms (‘slimmeriken’, wise guys), and use of ei/ij (diphthong [ɛi], i.e. ‘allerlei’, all kinds). Participants are presented with a list of sixty words, in pseudo-random order, divided into three columns of twenty words each. Half of the test words is spelled incorrectly. The ratio of correctly and incorrectly spelled words is not known to the participants. Participants are instructed to use their mouse to click the boxes next to words they think are spelled incorrectly. The participant’s performance indicator is the proportion correctly categorized misspelled words minus the proportion incorrectly selected words that are spelled correctly.

**8.5 Author recognition test**

We included a digital version of the Dutch author recognition test [78] to measure literary exposure. Participants are presented with a written list of 132 names, divided into three columns of 44 words each. They have to indicate which of the listed persons are authors (e.g., Roald Dahl, Nicci French). Ninety of the listed persons are authors and 42 are non-author foils. Authors and non-authors are listed in pseudo-random order and the ratio of authors/non-authors is not known to participants. The performance indicator is the proportion of correctly identified authors minus the proportion non-authors wrongly selected.

**8.6 Prescriptive grammar test**

To assess participants’ knowledge of Dutch prescriptive grammar, a recently developed grammaticality judgement test [79] is used. Participants hear spoken sentences and indicate for each of them whether they think it is a correct Dutch sentence. The sentences features five grammatical categories (eight trials per category, 50% correct), which adult native speakers of Dutch often find difficult to use correctly: personal pronouns (‘ze’, they vs. ‘hun’, their; ‘ik’, I vs. ‘mij’, me), comparatives (‘als’, as vs. ‘dan’, than), relative pronouns (‘die’, this vs. ‘dat’, that) and participle formation of complex verbs (e.g., ‘stofzuigen’, to vacuum). Stimuli were recorded in a soundproof booth. Average sentence duration is 4,344 ms (*SD* = 653, range = 3056 - 5901).

Each trial starts with the presentation of a fixation cross, which coincides with the playback of the spoken sentence. The fixation cross remains in view for the duration of the sentence. Each sentence is presented only once. Participants can respond during or after the presentation of the sentence by mouse-clicking on the appropriate button on the screen (labeled ‘correct’, right-hand position and ‘incorrect’, left-hand position). The mouse-click terminates the trial. The inter-trial interval is 500 ms. The performance indicator is the proportion of correct responses

**General cognitive skills tests: Non-verbal processing speed**

*Auditory reaction time (A-RT) tests.* Two tests tapping response speed to auditory stimuli are used [80]. In both cases, the task is to respond as fast as possible to the onset of an auditory stimulus.

**8.7 Auditory simple reaction time test**

In the simple A-RT test, participants see a fixation cross in the center of the screen. After an interval varying between one and three seconds, a sine tone (550 Hz, 400 ms long) is played. Participants are instructed to press the space bar as soon as they hear the tone, which terminates the trial. After one second, the next trial begins. The simple A-RT test consists of twenty test trials, preceded by eight practice trials. Participants’ mean RT may serve as the performance indicator.

**8.8 Auditory choice reaction time test**

In the choice A-RT test, the task is to respond as quickly as possible to each of two auditory stimuli, presented in pseudo-random order, by pressing the associated button. Participants first see a fixation cross in the center of the screen. After an interval varying between one and three seconds, a low or high sine tone (300 and 800 Hz, respectively, both 400 ms long) is played. Participants press the M-key on their keyboard when they hear the high tone, and the Z-key when they hear the low tone. The inter-trial interval is one second. The choice A-RT test consists of forty experimental trials, which are preceded by sixteen practice trials. Average RT, calculated based on correct responses, may serve as the performance indicator.

**8.9 Letter comparison**

The letter comparison test was adapted from a paper-and-pencil task [81, 82]. We use the computerized version by Huettig and Janse [83]. The participants’ task is to decide whether or not two strings of letters are identical. The first test block featurs pairs of three-letter strings (e.g. TZF) and the second block pairs of six-letter strings (e.g. RNHKTG). Letters are presented in a large monospaced font (Courier New, font size 70). The space between the letter strings in a pair is 300 pixels. Participants are asked to indicate as quickly and accurately as possible whether the two letter strings are the same or different by pressing the Z-key (‘different’) or the M-key (‘same’). To start, there are six three-letter practice trials. Each test block consists of twelve trials containing identical strings and twelve trials with different strings. Each trial starts with the presentation of a fixation cross, which stays on the screen for 600 ms. Subsequently, the two letter strings are presented until a response is made. The next trial begins after one second. Response speed, determined by averaging over participants’ correct responses, may serve as performance indicator.

**Visual reaction time tests (V-RT)**

These tests are the visual counterparts of the A-RT tests. Participants are asked to respond as quickly as possible to the onset of a visual stimulus by pressing the correct button on their keyboard. Both V-RT tests are based on tests designed by Deary *et al.* [84].

**8.10 Visual simple reaction time test**

On each trial of the simple V-RT test, participants first see a fixation cross in the center of the screen. After an interval varying between one and three seconds, it is replaced by a line drawing of a triangle (200 x 200 pixels, black contours). Participants are instructed to press the spacebar as soon as the triangle appears. The response terminates the trial. After an inter-trial interval of one second, the next trial begins. The test consists of twenty experimental trials, preceded by eight practice trials. Participants’ average RT may serve as performance indicator.

**8.11 Visual choice reaction time test**

On each trial of the choice V-RT test, participants first see a fixation cross in the center of the screen. After an interval varying between one and three seconds, it is replaced with a line drawing of either a star or a circle (black contours, 200 x 200 pixels). Participants are instructed to press the Z-key as fast as possible upon appearance of a star, and the M-key upon appearance of a circle. The star and circle appears equally often throughout the experiment and in pseudo-random order. The test consists of forty experimental trials, preceded by sixteen practice trials. Participants’ average RT on correct trials may serve as the performance indicator.

**General cognitive skills tests: Working memory**

**8.12 Digit span (forward and backward)**

We use a computerized version of the digit span test [85] to assess auditory working memory. At the beginning of each trial, a fixation cross appears in the center of the screen. After 2,000 ms, the playback of a sequence of spoken digits is initiated while the fixation cross remains in view. Digits are presented at approximately 500 ms intervals. Following auditory playback, a response field appears at the bottom of the screen and participants are requested to type in the digits in the order they are encountered (forward version) or in the reversed order (backward version). The first two trials of each version features a two-digit sequence; these are considered practice trials. When at least one of two consecutive trials of the same length is recalled correctly, the sequence is extended by one digit. The test ends when two consecutive trials for a sequence length are responded to incorrectly or when participants reaches the end of the test (nine digits in the forward version, eight digits in the backward version). Separate performance indicators are obtained for forward and backward versions, operationalized as the sum of correct responses per version [86].

**8.13 Corsi block clicking (forward and backward)**

This test is included to assess visual-spatial short-term memory capacity [87, 88] and forms the counter-part to the auditory digit span test. Participants are presented with nine squares, which are randomly distributed across the screen. Different squares lit up successively at a rate of one square per second. At the end of a sequence, a green frame appears around the display, prompting participants for a response. The participants are instructed to repeat the sequence by clicking on the respective squares, either by forward repetition or backward reproduction. When clicking on the squares, they briefly lit up in black for 200 ms and then turn blank again. After having reproduced the sequence in forward or backward fashion, participants click on a button at the bottom of the screen to proceed to the next trial. They are familiarized with the test by completing two practice trials of two-square sequences. The first experimental trial features a sequence length of three squares. The sequence length is extended by one square when at least one of two consecutive trials is recalled correctly. The test ends when two consecutive trials for a given sequence length are responded to incorrectly or when participants reaches the end of the test (sequence of nine blocks in both versions). The performance indicator is the sum of correct responses on experimental trials in forward and backward versions, respectively.

**General cognitive skills test: Non-verbal intelligence**

**8.14 Raven’s advanced progressive matrices test**

To assess non-verbal intelligence, a computerized version of Ravens’ advanced progressive matrices [89] is used. On each trial, participants indicate which of eight possible shapes completed a matrix of geometric patterns. They select the shape by clicking on it. Participants can skip items by clicking on a button labeled ‘Skip’; these items are shown again at the end of the test. When they do not know the answer to a skipped item, participants can click on an ‘I don't know’ button. There are 36 test items, increasing in difficulty, preceded by six untimed practice items. Participants have twenty minutes to complete the experimental items. Throughout the test, a clock in the right top corner of the screen shows the time remaining. The performance indicator is the proportion of correctly answered experimental items.

**Linguistic processing skills tests: Word production**

**8.15 Picture naming**

To test participants’ word production skills, we include a picture-naming test. In this test, participants are shown photographs of common objects and are asked to name these as quickly as possible [80]. The test materials consists of forty photographs, taken from de Groot *et al*. [90] or retrieved online via a search engine. The object names vary substantially in lexical frequency (*M* ZipfF = 3.85, *SD* = 0.86, range = 2.04-5.39). Prevalence norms indicate that the object names are likely to be known by all participants (*M* prevalence 99.6%, *SD* = 0.4, range 97.7-100). The average number of phonological neighbors (sum of additions, substitutions, deletions of segments) of the object names is 4.03 (*SD* = 4.10, range = 0-18; as retrieved from CLEARPOND [91]). Four additional photographs are used as practice trials. All pictures were scaled to 300 x 300 pixels.

The test begins with the presentation of the four practice items. On each trial, participants first see a fixation cross in the center of the screen, which is shown for 800 ms. Then, the target picture is shown for three seconds. After an inter-trial interval of one second, the next trial begins. Participants’ utterances are recorded. Participants’ average onset latency for correctly named experimental trials may serve as performance indicator.

**8.16 Rapid automatized naming (RAN)**

This test was included to assess speed of word form access during word production. Participants are first familiarized with a set of five line drawings and their names. During this familiarization phase, participants see the line drawings and hear a recording of a native Dutch speaker name each of them. Subsequently, they see the line drawings randomly arranged in an array consisting of five rows of six objects; each object is thus repeated six times throughout the array. At the beginning of the trial, a fixation cross is shown in the left upper corner of the screen, at the position of the first object of the array. The fixation cross disappears after two seconds and the object array is shown. Participants name all objects row-by-row, from left to right. They are instructed to name them as quickly as possible, while making as few mistakes as possible. Upon completion, they click on a button at the bottom of the screen to advance to the next run.

We use a version of the RAN developed for Dutch at the Max Planck Institute for Psycholinguistics. The test features four sets of five line drawings whose names orthogonally varied in word frequency, and neighborhood density, thereby comprising an easy set (high frequency: *M* ZipfF = 4.94, high density: *M* = 25, set 1), a hard set (low frequency: *M* ZipfF = 3.5, low density: *M* = 8, set 4) and two intermediate sets (high frequency: *M* ZipfF = 4.95, low density: *M* = 10, set 2; low frequency: *M* ZipfF = 3.55, high density: *M* = 23, set 3). Each set is named twice, featuring different configurations of the line drawings.

The performance indicator may be operationalized as the ratio between correctly produced object names and the speech duration within a run, yielding eight scores (one for each run), which can be combined or analyzed separately. Runs with substantial amounts of errors (e.g., one of the objects consistently named incorrectly, not completing the run, task misunderstandings) should be excluded.

**8.17 Verbal fluency**

We included a digitized version of the verbal fluency test used in an earlier study with Dutch participants [92]. In the first part of this test, participants are presented with two semantic categories (‘animals’ and ‘food & drinks’), one at a time. They are told they will have one minute to name as many words belonging to the categories as they can. The second part of the test was similar, however, participants are now presented with a letter (‘M’ and ‘S’) and have to produce as many unique words as possible beginning with that letter. Each trial starts with a timer counting down from three to zero indicating the start of the recording. Then, the category or letter is presented. A timer is shown on the screen counting down from sixty to zero. After one minute, the next trial starts automatically. Participants click on a button on the screen to bring up the next category/letter. The average number of unique words produces in one minute for categories and letters, respectively, may serve as performance indicators (i.e., two performance indicators per participant; see Shao *et al.* [92] for scoring details).

**8.18 Antonym production**

As an additional test of lexical access ability, focusing on the activation of semantic representations, an open-ended, untimed antonym production test was included. This test is an adapted version of the test recently developed by Mainz *et al*. [93]. Participants are provided with a word cue and are instructed to produce its antonym (e.g., cue: hot, antonym: cold). The test consists of 28 trials (3 practice and 25 experimental trials). Before each trial, participants see a fixation cross for 500 ms, after which the cue word wis presented (in written form *and* once in spoken form). Participants provide a spoken response and their answer is recorded. They click on a button on the screen to advance to the next trial. The cue words vary in word frequency (*M* ZipfF = 3.74, *SD* = 0.75, range = 1.70 - 5.00) and prevalence (*M* = 0.98, *SD* = 0.04, range = 0.85 - 1.00) and thus in how easily an antonym can be retrieved. Accuracy, operationalized as the proportion of correct antonyms produced, may serve as the performance indicator.

**8.19 Maximal speech rate**

To assess their maximal speech rate, participants are asked to recite the months of the year as quickly as possible with good pronunciation. Participants perform two runs. The average speech duration of both runs may serve as the dependent variable. In case only one of the two runs was correct, the speech duration of the correct run should be analyzed further.

**Linguistic processing skills tests: Sentence production**

**8.20 Phrase generation**

In this recently developed phrase generation test, participants produce object descriptions of varying complexity. All displays refer to a small set of objects, which minimize between-item variability due to differences in ease of lexical access.

We selected sixteen photographs of common every-day objects from the De Groot *et al. [90]* database to be used in the test. Their names are high in word frequency (*M* ZipfF = 4.57, SD = 0.44, range = 3.85 - 5.23) and prevalence (M = 1.00, SD = 0.00, range = 0.99 – 1.00). All object names are monosyllabic. Half of the words have common gender and half are neuter. The objects are repeated in different combinations across four blocks of the test. Additionally, we select two color adjectives (‘blauw’, blue, ‘geel’, yellow) and two numerals (‘twee’, two, ‘drie’, three) for the generation of complex noun phrases. Adjectives and numerals are chosen to be easy, as well as perceptually and phonologically distinct.

Before the first block, participants are familiarized with the sixteen objects and their names. Four of the objects are used as practice items, the other twelve objects are used as experimental items.

Each of the four blocks consists of twelve trials. At the beginning of each trial, participants see a fixation cross in the center of the screen for 500 ms, which is subsequently replaced with the to-be-named object(s). The start of the recording of participants’ speech coincides with the presentation of the visual stimulus. Participants are instructed to name the object(s) presented on the screen as quickly as possible and then press the spacebar. On pushing the spacebar, the screen goes blank while the recording continues for one second. This interval is included to account for cases where participants press the spacebar before they finish speaking.

In the first block, participants name single objects (e.g. ‘hond’, dog). In the second block, participants are presented with two objects that are placed next to each other, and are asked to name both objects using the conjunction ‘en’ (and, e.g., ‘aap en neus’, ‘monkey and nose’). In the third block, the visual stimulus is either a colored in object, requiring the use of a color adjective, (e.g., ‘blauwe hond’, blue dog) or consists of multiple objects of the same kind, requiring participants to produce plural forms (e.g., ‘twee honden’, two dogs). Finally, in the fourth block, participants have to name colored-in plural objects (e.g., ‘twee blauwe honden’, two blue dogs). Speech onsets for correctly produced utterances my serve as performance indicators.

**8.21 Sentence generation (structured)**

This test was included to assess individual differences in the time course of sentence formulation [94]. Participants describe photographs generating transitive and intransitive sentences. The task consists of eighty items: forty experimental (transitive sentences; twenty active, twenty passive) and forty filler items (intransitive sentences), presented in pseudorandom order. Experimental items are interspersed with filler items to reduce the likelihood of self-priming of a given syntactic structure [95]. The pictures for the task were taken from Menenti *et al*. [94] and consisted of eighty photographs in which one or two actors carry out transitive or intransitive actions. Transitive events feature one male and one female character, eliciting active sentences such as ‘The man kisses the woman’ (Dutch: ‘De man kust de vrouw’) or passive sentences such as ‘The woman is being kissed by the man’ (Dutch: ‘De vrouw wordt door de man gekust’). Intransitive actions feature either a male or a female character (e.g., ‘The woman eats’, Dutch: ‘De vrouw eet’). Using Adobe Photoshop©, the actors (agent and patient) carrying out a transitive event were colored in in yellow and blue, respectively; the actor (agent) carrying out an intransitive event was colored in in yellow. The actors’ color-coding in the transitive event pictures as well as their position in the photographs (left or right) are counterbalanced.

Each trial starts with the playback of an active or passive transitive or intransitive prime sentence spoken by a male native speaker of Dutch (mean length = 2113, SD = 261, range = 1670-2669). The prime sentence has the same structure as the target sentence of that trial, but uses a different verb. Moreover, on half of the trials, agent and patient sex is different in prime and target sentences to ensure that participants look at the photograph to derive agent and patient roles. Next, a screen appears for 500 ms featuring either a transitive or an intransitive verb. Verbs have a mean Zipfian word frequency of 4.01 (SD = 1.15, range = 1.85-5.78) and a mean word prevalence of 0.99 (SD = 0.22, range = 0.89-1.00). The recording of participants’ speech starts with the presentation of the photograph. Participants are instructed to describe the photograph using the verb they just saw and to press the spacebar when done speaking. They are instructed to mention the yellow character in the photograph first. Depending on the color of the agent and patient in a given event, either an active or a passive sentence has to be produced. After pressing the space bar, the screen goes blank, while the recording continues for 500 ms.

Participants carry out three practice trials before the experimental trials. Speech onset latencies for correctly produced sentences and speech duration may serve as performance indicators.

**8.22 Sentence generation (unstructured)**

A second sentence production test was included. However, in contrast to test 21, here, participants generate spontaneous, unstructured sentences. On each trial, participants are presented with a picture of an event and are asked to describe the event in a single sentence, naming all characters taking part in the event. The test consists of 120 trials, 60 of which are target items and 60 are filler items. The latter are randomly interspersed with the target items to avoid self-priming of a given syntactic structure [95]. Target items depict transitive events whereas filler items depict intransitive events. Both target and filler items feature events with human and non-human agents/patients.

The picture selection was based on the results of an earlier study by Konopka *et al.* [96]. In that study, 38 native Dutch speakers produced transitive and intransitive sentences cued by cartoon-like pictures. We re-analyzed the data by Konopka *et al.* [96] and obtained the proportion of active and passive responses for each item in that study (240 items in total). For the present study, we selected thirty pictures that have elicited active sentences and thirty pictures that have elicited passive sentences (proportion of passive responses varies from item to item and was on average 45%, SD = 24, range 6 - 95).

The trial structure is as follows: Participants are presented with the to-be-described picture (500 x 500 pixels) in the center of the screen. Coinciding with the picture presentation, a recording of participants’ speech is started. They describe the picture and advance to the next trial by mouse-clicking on a button underneath the picture or they are timed out after ten seconds. On clicking the button, the screen goes blank while the recording continues for one second. No more than two pictures assumed to either elicit active or passive responses, respectively, are placed in sequence. Left/right placement of agent and patient is counterbalanced across all trials. Experimental trials are preceded by three practice trials (one intransitive sentence and two transitive sentences). After each practice trial, an example answer is provided. The proportion of produced active and passive sentences, onset latency for grammatically correct sentences, speech duration or the proportion of disfluencies may serve as performance indicators.

**8.23 Spontaneous speech**

In this test, participants are asked to talk freely about three topics: (1) their activities during the last weekend, or any other weekend of their choice, (2) a book or a movie they have recently enjoyed and (3) their dream holidays. These topics were chosen as they are expected to elicit natural speech in the past, present, future and conditional tenses. Such tasks have been used widely in the neuropsychological and aging literature and allow for the analysis of various properties of spontaneous speech including speech rate, fluency (frequency and duration of silent and filled pauses), type-token ratio of words, average word frequency, utterance length, syntactic and conceptual richness. Moreover, in a recent pilot study, we showed that measures extracted from spontaneous speech correlated positively with participants’ receptive vocabulary size [97].

Participants are presented with the questions (one at a time) and are given time to conceptualize what they want to say. When ready, they click on a button to start the recording. After one minute, the recording stops automatically, and participants are presented with the next question.

**Linguistic processing skills tests: Word comprehension**

**8.24 Non-word monitoring in noise in non-word lists**

This task assesses participants’ ability to extract phonological information from noisy input and map it onto stored mental representations. The task involves monitoring spoken lists of non-words for the occurrence of targets that matches an auditory cue. The cue, a monosyllabic non-word, is provided at the beginning of each trial and varies from trial to trial. While the cue is presented in the clear, the subsequent lists of three to six non-words are presented in stationary speech-shaped background noise. On the first trial, the signal-to-noise ratio (SNR) is set to -10 dB (i.e. more portions of noise than speech) and decreases from trial to trial in steps of two until -24 dB. Each SNR is presented six times (i.e., six rounds of going from SNR -10 dB to SNR -24 dB).

The task consists of 48 trials (see below for examples): sixteen are target-absent trials, where the cue non-word does not appear in the list; sixteen are target-present trials; and sixteen are ‘target-present plus foil trials’. On these trials, the target is preceded by a non-word foil that overlap with the target in phonological onset (on average two phonemes). We included these trials to ensure that participants listen carefully until the end of each non-word. We use different recordings of the same non-word for cue and target presentations to avoid response strategies based on low-level perceptual matching. Non-word cues are on average 768 ms long (*SD* = 130, range = 460 - 1139); targets are on average 802 ms long (*SD* = 134, range = 576 - 1049). The target word position within the list varies from trial to trial; it never occurs in list-initial or list-final position.

*Examples of trials*

1. Target present: Cue non-word: **broon**. List: nijmsaard – wulen – pluif – **broon –** swi
2. Target + foil: Cue non-word: **broog**. List: dauk – broopkimp – **broog** – knekgel
3. Target absent: Cue non-word: **praan**. List: veg – gebog – siekoed – fonguin

Non-word cues and distractors were created on the basis of existing Dutch words using the non-word generator Wuggy [98]. The generated non-words match the number of letters and the number of phonemes in the original word. Audio recordings of the stimuli were made in a soundproof booth. Speech-shaped noise was added to each individual file using Praat software [99]. To that end, the original recordings were down-sampled to sixteen kHz to match the sampling frequency of the noise. Two-hundred ms of ramping noise precede the speech onsets, providing a head start for the listeners to get used to the noise. Noise was added over the entire file. Peak intensity in all (clear and noise-added) files was set to 65 dB.

The trial structure is as follows: Participants press the spacebar on the screen to start the trial; a fixation cross is displayed in the center of the screen and after 200 ms the spoken cue is presented. After an interval of one second, the list is presented, with 500 ms of silence between non-words. The fixation cross remains visible throughout the playback. Participants are instructed to press the spacebar as soon as they recognize the cue within the list. Pressing the button terminates the trial. Button presses are counted as correct responses when they occur within the playback period of the target word or non-word, or 500 ms after its offset. Before the first experimental trial, participants complete six practice trials. The performance indicator is the proportion of correct responses to target-present trials minus the proportion of false alarms on target-absent trials.

**8.25 Rhyme judgment**

Similar to the non-word monitoring task, the rhyme judgment test assesses phonological mapping abilities. On each trial, participants are presented with two monosyllabic non-words and are asked to judge as fast as possible whether they rhyme. Rhyme overlap is defined as an overlap in the vowel and the following consonant(s). The test consists of 40 experimental trials: 24 of which are ‘rhyming trials’ (e.g., ‘noost’-‘woost’) and require a yes-response. Eight are ‘non-rhyming trials’ (e.g., ‘beus’-‘fuug’) and require a no-response. Another eight trials are ‘non-rhyming foils’, which features non-word pairs sharing the vowel but not the following consonants (e.g., ‘bruip’, ‘fluik’). These trials were included to ensure that participants listen carefully until the end of the second non-word [100]; they also require a no-response. Rhyming and non-rhyming trials are presented in a pseudo-random order. Prior to the experimental trials, participants complete four practice trials (two rhyming, one non-rhyming and one non-rhyming foil, in random order). The non-words used in the test are based on existing Dutch words and were generated using Wuggy [98]. Recordings of the non-words were made in a sound-shielded booth. Length of first and second non-words in the three trial types are as follows: Rhyming items: first word average = 670 ms, *SD* = 86, range = 470-810; second word average = 621 ms, *SD* = 74, range = 521-770; Non-rhyming items: first word average = 653 ms, *SD* = 58, range = 547-720; second word average = 562, *SD* = 64, range = 461-638; Non-rhyming foils: first word average = 588 ms, *SD* = 88, range = 443-701; second word average = 550 ms, *SD* = 62, range = 449-649.

The trial structure is as follows: A fixation cross appeares in the center of the screen for 500 ms. Then, the first non-word is played back. Five-hundred ms after the offset of the first non-word, the second non-word is played back. Participants are instructed to indicate as fast as possible whether the non-words rhyme by pressing a key on the keyboard (M-key for ‘rhyme’, Z-key for ‘no rhyme’). Their response and its latency, measured from the onset of the second non-word, are recorded. The button press terminates the trial. The inter-trial interval is 2,000 ms. Participants’ average RT, based on correct responses to rhyming trials, may serve as performance indicator.

**8.26 Auditory lexical decision**

This test was included to measure lexical access speed. Participants are instructed to listen to the recording of a word or non-word and judge whether it exists in the Dutch language [80]. To that end, sixty Dutch words were selected from the Subtlex database. The words vary substantially in word frequency (*M* ZipfF = 3.65, *SD* = 0.85, range = 2.04-5.66), but are highly prevalent and thus well known to all participants (*M* prevalence = 99.6, *SD* = 0.5, range = 97.3-100). The average number of phonological neighbours (as retrieved from Marian *et al*. [91] defined as deletions, additions, and substitutions) was 2.8 (*SD* = 3, range = 0-12). For each word, a matched non-word was created using Wuggy ([98]; applying the same constraints as for the non-word monitoring and the rhyme judgement task). One additional word and two non-words are used as practice trials. Recordings were made in a sound-attenuated booth. The average stimulus length is 746 ms (*SD* = 93, range = 568 – 967) for words and 808 ms (*SD* = 119, range = 540 – 1164) for non-words.

The test begins with the presentation of the practice items, followed by the experimental items, both presented in a pseudo-random order. At the beginning of each trial, a fixation cross is shown in the center of the screen for 300 ms. Then the stimulus is presented. Participants are instructed to listen carefully to each stimulus and decide as quickly as possibly whether or not it is an existing Dutch word. They press the M-key on the keyboard to give a ‘word’ response and the Z-key to give a ‘not a word’ response. The response terminates the trial. After one second, the next trial begins. The response latency is the time interval between the spoken word onset and the button press. The average response latency on correct responses to words may serve as performance indicator.

**8.27 Semantic categorization**

We included a semantic categorization test to tap semantic access during spoken word recognition. There are two test blocks. At the beginning of a block participants are presented with a semantic category, ‘professions’ for block one and ‘means of transportation’ for block two. On each of the following trials, they hear a word and have to judge whether or not it is a member of that category. We selected professions and means of transportation categories as they feature enough easy-to-recognize members. Each test block consists of 32 trials, 20 category members and 12 distractors. Each part is preceded by four practice trials (two category members, two distractors). Targets and distractors were matched on word frequency (professions: *M* ZipfF = 3.56, *SD* = .37, range = 2.81-4.07; means of transportation *M* ZipfF = 3.27, *SD* = .51, range = 2.15-4.12). All words used in the test are highly prevalent (known to 99-100% of all people). Recordings were made in a sound-shielded booth. The recordings of words have the following durations: Professions: *M* = 850 ms (*SD* = 137, range = 579 – 1104), Distractors: *M* = 775 ms (*SD* = 125, range = 602 – 1001); Means of transportation: *M*: 745 ms (*SD* = 167, range = 510 – 1055), distractors: *M* = 733 ms (*SD* = 193, range = 448 – 1079).

The trial structure is as follows: Participants are first presented with a fixation cross for 500 ms after which they hear the spoken word. They are instructed to indicate as fast as possible whether the word belonges to the category provided beforehand by pushing the associated key on the keyboard (M-key for ‘yes, this word belongs to the category’ and Z-key for ‘no, this word does not belong to the category’). The inter-trial interval is 2,000 ms. Response latency is measured from the onset of the spoken word. Participants’ average RT, based on correct responses to semantic category members, may serve as the performance indicator.

**Linguistic processing skills tests: Sentence comprehension**

**8.28 Word monitoring in noise in sentences**

This sentence comprehension test is similar to the word-level counterpart (20. Non-word monitoring in noise in non-word lists). However, rather than monitoring for non-words in lists, participants monitor sentences for words that are predictable or non-predictable from the sentence context.

The test consists of three practice and sixty experimental trials. On each trial, participants hear a word cue presented in the clear, followed by a sentence presented in stationary speech-shaped background noise. As in the non-word monitoring task, the SNR decreases from trial to trial in steps of two, starting at an SNR of -12 dB until -20 dB. Each SNR is repeated twelve times (i.e., twelve rounds of going from SNR -12 to -20). Forty of the sixty trials are target-present items, where the cue is part of the sentence. These trials require a button press. The remaining twenty trials are target-absent items and do not require a button press.

The sentences were selected from the materials previously used by Piai *et al.* [101]. One to seven words were added to the ends of these sentences such that the target word is never the final word in the sentence. In half of the target-present items, the cue word can be predicted based on the sentence context (mean cloze probability = 1, SD = 0.02, range 0.93-1.00 [101]). Similarly, half of the target-absent trials are predictive of a particular word, which is, however, not the cue word. Target-present and target-absent trials occur equally often in each SNR. Predictable and non-predictable trials are presented in a pseudo-random order. Recordings were made from a native Dutch speaker in a sound-shielded booth. To avoid voice familiarity effects when listening in noise, we recorded a different speaker than the one who had produced the stimuli for test 20. Non-word monitoring in noise in non-word lists. Cue words are on average 676 ms long (SD = 96, range = 399 - 850); sentences are on average 4460 ms long (SD = 595, range = 3201 - 5586). On target-present trials, cue words occur after on average 2538 ms (SD = 358, range = 1854 - 3346) and are on average 353 ms (SD = 6, range = 234 - 466) long in the running speech. Speech-shaped noise was added to recordings using Praat (Boersma, 2001). The noise was added over the entire recording.

The trial structure is as follows: Participants press the spacebar to start the trial. A fixation cross appears on the screen and the cue word is played back (in the clear). After a 500 ms interval, the sentence is played back (in speech-shaped background noise). Participants are instructed to press the spacebar when detecting the cue word in the sentence. Upon pressing spacebar, the fixation cross disappears, but the sentence presentation continues until the end of the sentence. Button presses are coded as correct when they occur within the period spanning 300 ms prior to target word onset in the unfolding sentence, during its unfolding, or 500 ms thereafter. The difference between the proportion of correct responses to target-present trials and the proportion of incorrect responses on target-absent trials (false alarms) is calculated for the predictable and the non-predictable condition separately. The performance indicator is operationalized as the ‘predictability benefit’ (i.e., the difference between the correct-incorrect differences in the predictable vs. the non-predictable conditions).

**8.29 Gender cue activation during sentence comprehension**

This test assesses whether and to what degree listeners exploit grammatical gender cues for predicting upcoming target words in an unfolding sentence. The first part of the test is a gender judgment task, where participants are presented with 84 common objects [102], one at a time and are instructed to indicate the grammatical gender of the object names (42 ‘de’, common-gender nouns, 42 ‘het’, neuter-gender nouns) by pressing the associated key on the keyboard (Z-key for ‘de’, M-key for ‘het’). This part is included to tap participants’ accuracy in judging a word’s grammatical gender and to increase their sensitivity toward this type of word knowledge. The mean Zipfian word frequency of the object names is 4.05 (*SD* = 0.66, range = 2.36 – 5.66). Prevalence of the object names is high (*M* = 1.00, *SD* = 0.00 range = 0.98 – 1.00). Objects with neuter and common gender (‘het’ and ‘de’ items) are presented in pseudo-random order. The participant’s button press terminates the trial.

The second part of the test was inspired by a study by Huettig and Janse [83]. The same objects as in the first part are used. The second part consists of two practice trials and forty experimental trials. Each trial features two objects which differ in grammatical gender (i.e., one het-word, neuter gender and one de-word, common gender). Participants are first presented with a fixation cross for 800 ms in the center of the screen, followed by the presentation of the two objects. Then, they hear the recording of a question asking on which side of the screen (left or right) one of the two objects is located (e.g., ‘Waar is het weergegeven paard?’, Where is the displayed horse?). Crucially, on half of the trials, the definite article is used (de/het, the); on the other half, the indefinite article is used (‘een’, a; e.g. ‘Waar is een weergegeven boek?’, Where is a displayed book?). Trials featuring the definite article constitutes the predictable condition as participants can anticipate, based on the definite article’s grammatical gender, which object will be referred to. Trials featuring the indefinite article constitute the non-predictable condition, as based on the indefinite article, it cannot be anticipated which object is referred to (the indefinite article in Dutch is not marked for gender). The presentation of the spoken sentence is timed such that on each trial participants have three seconds to preview the two objects before the onset of the gender cue in the spoken sentences (i.e., sufficient time to retrieve this type of word information from the displayed objects [83]). The definite articles (predictable condition) are on average 295 ms long (*SD* = 46, range = 227 – 355); the indefinite articles (non-predictable condition) are 315 ms long (*SD* = 18, range = 286 – 341). The target words in the predictable condition are on average 583 ms long (*SD* = 133, range 383-971); in the non-predictable condition they are on average 583 ms long (*SD* = 119, range = 370 – 788). The predictive window (i.e., the interval between determiner onset and target word onset on the 20 predictable trials) is on average 2221 ms long (*SD* = 98, range = 2032 – 2455).

Participants are instructed to press the appropriate button as soon as they know which object is referred to (Z-key for left-hand object, M-key for right-hand object). The button press terminates the trial. The inter-trial interval is 200 ms. The target object appears equally often on the left and right side of the screen. Predictable and non-predictable trials are presented in a pseudo-random order. Response latencies are calculated as the difference between the onset of the target word in the spoken sentences and participants’ button press. A negative value thus reflects a button press before target word onset. The average RT, based on correct responses in the predictable condition, may serve as performance indicator.

**8.30 Verb semantics activation during sentence comprehension**

Using a similar paradigm as for test 29. Gender cue activation during sentence comprehension, this test assesses participants’ ability to use verb-specific selectional restrictions during unfolding spoken sentences [103, 104]. On each trial, participants first see a fixation cross in the center of the screen for 800 ms, which is replaced with pictures of two common objects (e.g., an apple and a table; *M* Zipfian object-name frequency = 4.13, *SD* = 0.64, range = 2.70 – 5.52; *M* object-name prevalence = 1.00, *SD* = 0.01, range = 0.97 – 1.00 [102]). Next, they hear a spoken sentence describing a transitive action carried out by a fictional character (e.g. ‘De man schilt op dit moment een appel.’, The man is peeling at this moment an apple). In the predictable condition, participants can predict, based on the verb semantics, which of the objects is referred to before target word onset as only one fulfills its selectional restrictions (e.g., an apple can be peeled, a table cannot). In the non-predictable condition, this is not the case.

The verbs in the predictable condition are on average 598 ms long (*SD* = 132, range = 406 – 875); verbs in the non-predictable condition are on average 641 ms long (*SD* = 153, range = 403 – 1037). Target words in the predictable condition are on average 471 ms long (*SD* = 96, range = 363 – 731) and 525 ms long (*SD* = 145, range = 313 – 931) in the non-predictable condition. The predictive window (i.e. the interval between verb onset and target word onset on the predictable experimental trials) is on average 2598 ms long (*SD* = 218, range = 2131 – 2922).

The participants’ task is to press the appropriate button (Z-key for left-hand object on screen, M-key for right-hand object on screen) as soon as they know which object is referred to. Their button press terminates the trial. The inter-trial interval is 200 ms. The test consists of forty experimental trials, twenty of which are predictable and twenty are non-predictable. Experimental trials are preceded by two (one predictable, one non-predictable) practice trials. The onset of the spoken sentence is timed such that participants have three seconds to preview the two objects prior to the onset of the verb in the spoken sentence. Response latencies are calculated as the difference between the onset of the target word in the spoken sentence and the participant’s button press. A negative value thus reflects a button press before target word onset. The average RT, based on correct responses in the predictable condition, may serve as the performance indicator.

**8.31 Self-paced reading**

We included a self-paced reading test to assess individual differences in comprehending sentences varying in syntactic complexity. Participants read Dutch sentences, presented to them in a word-by-word fashion. The test comprises forty experimental items and three practice items. The sentences are a subset of the materials used by Schoffelen *et al*. [105]. Thirty of the forty experimental items contain a relative clause. The sentences are between nine and fifteen words long and featured substantial variation in syntactic complexity, as assessed using left-branching and right-branching complexity metrics. Specifically, we determined for each word in a sentence the number of simultaneously open left- and right-branching dependencies at that particular point in the sentence and summed these values to obtain one left-branching and one right-branching complexity value per sentence. The average left-branching complexity in the forty sentences is 20 (*SD* = 7, range = 8 – 39); the average right-branching complexity is 13 (*SD* = 4, range = 7 – 21).

The participants are instructed to read the sentences silently. They are asked to read at a quick pace while still being able to understand the contents of the sentence. The trial structure is as follows: A fixation cross appears in the center of the screen. After 1,000 ms, the fixation cross is replaced with the first word of the sentence. Participants read the word and press the spacebar to advance to the next word, which also appears in the center of the screen and replaces the first word. The process continues until the end of the sentence. Participants press the Enter key to start the next trial. The start of each sentence is indicated with a capital letter. The sentences do not contain punctuation or a full stop at the end.

To ensure that participants read the sentences carefully, 20% of the sentences are followed by a yes/no comprehension question. Questions either focus on the words occurring in the sentence (e.g., “Was the word “dentist” mentioned in the sentence?”), or on the semantic contents of the sentence (e.g., “Did the table collapse?”). Half of the questions require a yes-response.

Reading times for each word in a sentence are recorded and summed to obtain one RT per sentence. Accuracy to the comprehension questions may be used to filter participants who did not carry out the test seriously. Total reading time per sentence, corrected for sentence length and the words’ frequency, may serve as performance indicator.

Table 4: Overview of tests blocked by session. The order of tests within a session reflects the order in which the tests were administered. The test numbers refer to the order of test descriptions in text. Performance indicators marked with a * require transcription and/or annotation of speech recordings or pre-processing of reaction times before further analysis.

| **Session** | **Test** | **Performance indicator** | **Duration** |
| --- | --- | --- | --- |
| 1 | 0. Questionnaire | / | 3 |
|  | 14. Raven’s advanced progressive matrices | Accuracy | 25 |
|  | 5. Author recognition test | Proportion of correct responses to authors – proportion of incorrect responses to foils | 5 |
|  | 13. Corsi block clicking (forward & backward) | Sum of correct trials (separate for forward and backward runs) | 7 |
|  | 1. Stairs4Words | Prevalence band | 7 |
|  | 12. Digit span (forward & backward) | Sum of correct trials (separate for forward and backward runs) | 7 |
|  | 4. Spelling test | Proportion of correct responses to correctly-spelled words – proportion of incorrect responses to incorrectly-speed words | 5 |
| 2 | 15. Picture naming | Mean onset latency* | 7 |
|  | 24. Non-word monitoring in noise in non-word lists | Proportion of correct responses to target-present trials – proportion of false alarms on target-absent trials | 10 |
|  | 26. Auditory lexical decision | Mean RT words* | 7 |
|  | 30. Verb semantics activation during sentence comprehension | Mean RT predictable condition* | 7 |
|  | 25. Rhyme judgment | Mean RT rhyming trials* | 5 |
|  | 9. Letter comparison | Mean RT* | 5 |
|  | 10. Visual simple reaction time test | Mean RT* | 3 |
|  | 11. Visual choice reaction time test | Mean RT* | 4 |
|  | 27. Semantic categorization | Mean RT category members* | 5 |
| 3 | 20. Phrase generation | Mean onset latency* | 10 |
|  | 21. Sentence generation (structured) | Mean onset latency* | 12 |
|  | 28. Word monitoring in noise in sentences | Difference between false-alarm corrected accuracy scores in predictable and non-predictable conditions | 10 |
|  | 31. Self-paced reading | Summed reading time per sentence* | 5 |
|  | 29. Gender cue activation during sentence comprehension | Mean RT predictable condition* | 10 |
|  | 7. Auditory simple reaction time test | Mean RT* | 3 |
|  | 8. Auditory choice reaction time test | Mean RT* | 4 |
| 4 | 22. Sentence generation (unstructured) | Proportion active/passive structures; mean onset latency* | 10 |
|  | 17. Verbal fluency | Average number of produced words (separate for semantic categories and letters)* | 5 |
|  | 16. Rapid automatized naming | Number of produced words per second* | 7 |
|  | 18. Antonym production | Accuracy* | 5 |
|  | 19. Maximal speech rate | Average speech duration* | 2 |
|  | 23. Spontaneous speech | /* | 3 |
|  | 6. Prescriptive grammar test | Accuracy | 10 |
|  | 2. Peabody picture vocabulary test | Raw score (last item – no. errors) | 10 |
|  | 3. Idiom recognition | Accuracy | 3 |

**9 References**

1.Kozey-Keadle S, Libertine A, Lyden K, Staudenmayer J, Freedson PS. Validation of wearable monitors for assessing sedentary behavior. Med Sci Sports Exerc. 2011;43(8):1561-7.

2.Ryan CG, Grant PM, Tigbe WW, Granat MH. The validity and reliability of a novel activity monitor as a measure of walking. British journal of sports medicine. 2006;40(9):779-84.

3.Winkler EA, Gardiner PA, Clark BK, Matthews CE, Owen N, Healy GN. Identifying sedentary time using automated estimates of accelerometer wear time. British journal of sports medicine. 2012;46(6):436-42.

4.Garbarino M, Lai M, Bender D, Picard RW, Tognetti S. Empatica E3 — A wearable wireless multi-sensor device for real-time computerized biofeedback and data acquisition. 2014 4th International Conference on Wireless Mobile Communication and Healthcare - Transforming Healthcare Through Innovations in Mobile and Wireless Technologies (MOBIHEALTH). 2014:39-42.

5.Dresler M, Spoormaker VI, Beitinger P, Czisch M, Kimura M, Steiger A, et al. Neuroscience-driven discovery and development of sleep therapeutics. Pharmacol Ther. 2014;141(3):300-34.

6.Dinan TG, Cryan JF. The microbiome-gut-brain axis in health and disease. Gastroenterol Clin North Am. 2017;46(1):77-89.

7.Lewis SJ, Heaton KW. Stool form scale as a useful guide to intestinal transit time. Scand J Gastroenterol. 1997;32(9):920-4.

8.Manders P, Lutomski J, Smit C, Swinkels D, Zielhuis G. The Radboud Biobank: A central facility for disease-based biobanks to optimise use and distribution of biomaterial for scientific research in the Radboud University Medical Center, Nijmegen. Open Journal of Bioresources. 2018;5.

9.Schwabe L, Haddad L, Schachinger H. HPA axis activation by a socially evaluated cold-pressor test. Psychoneuroendocrinology. 2008;33(6):890-5.

10.Miller R, Stalder T, Jarczok M, Almeida DM, Badrick E, Bartels M, et al. The CIRCORT database: Reference ranges and seasonal changes in diurnal salivary cortisol derived from a meta-dataset comprised of 15 field studies. Psychoneuroendocrinology. 2016;73:16-23.

11.Aerts R, Joly L, Szternfeld P, Tsilikas K, De Cremer K, Castelain P, et al. Silicone wristband passive samplers yield highly individualized pesticide residue exposure profiles. Environmental Science & Technology. 2018;52(1):298-307.

12.O’Connell SG, Kincl LD, Anderson KA. Silicone wristbands as personal passive samplers. Environmental Science & Technology. 2014;48(6):3327-35.

13.Wright KD, Hickman R, Laudenslager ML. Hair cortisol analysis: A promising biomarker of HPA activation in older adults. Gerontologist. 2015;55 Suppl 1:S140-5.

14.Van Essen DC, Ugurbil K, Auerbach E, Barch D, Behrens TE, Bucholz R, et al. The Human Connectome Project: a data acquisition perspective. Neuroimage. 2012;62(4):2222-31.

15.Miller KL, Alfaro-Almagro F, Bangerter NK, Thomas DL, Yacoub E, Xu J, et al. Multimodal population brain imaging in the UK Biobank prospective epidemiological study. Nat Neurosci. 2016;19(11):1523-36.

16.Bloem BR, Marks WJ, Jr., Silva de Lima AL, Kuijf ML, van Laar T, Jacobs BPF, et al. The Personalized Parkinson Project: examining disease progression through broad biomarkers in early Parkinson's disease. BMC Neurol. 2019;19(1):160.

17.Glasser MF, Sotiropoulos SN, Wilson JA, Coalson TS, Fischl B, Andersson JL, et al. The minimal preprocessing pipelines for the Human Connectome Project. Neuroimage. 2013;80:105-24.

18.Diaz BA, Van Der Sluis S, Benjamins JS, Stoffers D, Hardstone R, Mansvelder HD, et al. The ARSQ 2.0 reveals age and personality effects on mind-wandering experiences. Front Psychol. 2014;5:271.

19.Dubois J, Adolphs R. Building a science of individual differences from fMRI. Trends Cogn Sci. 2016;20(6):425-43.

20.Marques JP, Kober T, Krueger G, van der Zwaag W, Van de Moortele PF, Gruetter R. MP2RAGE, a self bias-field corrected sequence for improved segmentation and T1-mapping at high field. Neuroimage. 2010;49(2):1271-81.

21.Langkammer C, Schweser F, Krebs N, Deistung A, Goessler W, Scheurer E, et al. Quantitative susceptibility mapping (QSM) as a means to measure brain iron? A post mortem validation study. Neuroimage. 2012;62(3):1593-9.

22.Shanholtzer BA, Patterson SM. Use of bioelectrical impedance in hydration status assessment: reliability of a new tool in psychophysiology research. Int J Psychophysiol. 2003;49(3):217-26.

23.Lanier GM, Orlanes K, Hayashi Y, Murphy J, Flannery M, Te-Frey R, et al. Validity and reliability of a novel slow cuff-deflation system for noninvasive blood pressure monitoring in patients with continuous-flow left ventricular assist device. Circ Heart Fail. 2013;6(5):1005-12.

24.Åstrand PO, Ryhming I. A nomogram for calculation of aerobic capacity (physical fitness) from pulse rate during sub-maximal work. J Appl Physiol. 1954;7(2):218-21.

25.van Mil AC, Hartman Y, van Oorschot F, Heemels A, Bax N, Dawson EA, et al. Correlation of carotid artery reactivity with cardiovascular risk factors and coronary artery vasodilator responses in asymptomatic, healthy volunteers. J Hypertens. 2017;35(5):1026-34.

26.van Mil A, Pouwels S, Wilbrink J, Warlé MC, Thijssen DHJ. Carotid artery reactivity predicts events in peripheral arterial disease patients. Ann Surg. 2019;269(4):767-73.

27.van Mil A, Tymko MM, Kerstens TP, Stembridge M, Green DJ, Ainslie PN, et al. Similarity between carotid and coronary artery responses to sympathetic stimulation and the role of α(1)-receptors in humans. J Appl Physiol (1985). 2018;125(2):409-18.

28.Aitken RC. Measurement of feelings using visual analogue scales. Proc R Soc Med. 1969;62(10):989-93.

29.Collins SL, Moore RA, McQuay HJ. The visual analogue pain intensity scale: what is moderate pain in millimetres? Pain. 1997;72(1-2):95-7.

30.Timmerman H, Wilder-Smith OH, Steegers MA, Vissers KC, Wolff AP. The added value of bedside examination and screening QST to improve neuropathic pain identification in patients with chronic pain. J Pain Res. 2018;11:1307-18.

31.Constantino SM, Dalrymple J, Gilbert RW, Varanese S, Di Rocco A, Daw ND. A neural mechanism for the opportunity cost of time. bioRxiv. 2017:173443.

32.Tsetsos K, Pfeffer T, Jentgens P, Donner TH. Action planning and the timescale of evidence accumulation. PLoS One. 2015;10(6):e0129473.

33.Braun A, Urai AE, Donner TH. Adaptive history biases result from confidence-weighted accumulation of past choices. J Neurosci. 2018.

34.Thaler L, Schütz AC, Goodale MA, Gegenfurtner KR. What is the best fixation target? The effect of target shape on stability of fixational eye movements. Vision Res. 2013;76:31-42.

35.Therrien AS, Wolpert DM, Bastian AJ. Effective reinforcement learning following cerebellar damage requires a balance between exploration and motor noise. Brain. 2016;139(Pt 1):101-14.

36.Phillips LH, Wynn V, Gilhooly KJ, Della Sala S, Logie RH. The role of memory in the Tower of London task. Memory. 1999;7(2):209-31.

37.Shallice T. Specific impairments of planning. Philos Trans R Soc Lond B Biol Sci. 1982;298(1089):199-209.

38.Maren S, Phan KL, Liberzon I. The contextual brain: implications for fear conditioning, extinction and psychopathology. Nat Rev Neurosci. 2013;14(6):417-28.

39.Mühlberger A, Andreatta M, Ewald H, Glotzbach-Schoon E, Tröger C, Baumann C, et al. The BDNF Val66Met polymorphism modulates the generalization of cued fear responses to a novel context. Neuropsychopharmacology. 2014;39(5):1187-95.

40.Van Ast V, Vervliet B, Kindt M. Contextual control over expression of fear is affected by cortisol. 2012;6(67).

41.Klumpers F, Raemaekers MA, Ruigrok AN, Hermans EJ, Kenemans JL, Baas JM. Prefrontal mechanisms of fear reduction after threat offset. Biol Psychiatry. 2010;68(11):1031-8.

42.Krypotos A-M, Vervliet B, Engelhard I. The validity of human avoidance paradigms. Behaviour Research and Therapy. 2018;111.

43.Pittig A, Treanor M, LeBeau RT, Craske MG. The role of associative fear and avoidance learning in anxiety disorders: Gaps and directions for future research. Neurosci Biobehav Rev. 2018;88:117-40.

44.Kanske P, Heissler J, Schönfelder S, Bongers A, Wessa M. How to regulate emotion? Neural networks for reappraisal and distraction. Cereb Cortex. 2011;21(6):1379-88.

45.Lang PJ, Bradley MM, Cuthbert BN. International affective picture system (IAPS) : affective ratings of pictures and instruction manual. Gainesville, Fla.: NIMH, Center for the Study of Emotion & Attention; 2005.

46.Wessa M, Kanske P, Neumeister P, Bode K, Heissler J, Schönfelder S. EmoPics: subjektive und psychophysiologische Evaluationen neuen Bildmaterials für die klinisch-bio-psychologische Forschung. Z Klin Psychol Psychother. 2010;1:S11-S77.

47.Derry PA, Kuiper NA. Schematic processing and self-reference in clinical depression. J Abnorm Psychol. 1981;90(4):286-97.

48.Hammen C, Zupan BA. Self-schemas, depression, and the processing of personal information in children. J Exp Child Psychol. 1984;37(3):598-608.

49.Gotlib IH, Kasch KL, Traill S, Joormann J, Arnow BA, Johnson SL. Coherence and specificity of information-processing biases in depression and social phobia. J Abnorm Psychol. 2004;113(3):386-98.

50.Vrijsen JN, Dainer-Best J, Witcraft SM, Papini S, Hertel P, Beevers CG, et al. Effect of cognitive bias modification-memory on depressive symptoms and autobiographical memory bias: two independent studies in high-ruminating and dysphoric samples. Cogn Emot. 2019;33(2):288-304.

51.Vrijsen JN, Tendolkar I, Onnink M, Hoogman M, Schene AH, Fernández G, et al. ADHD symptoms in healthy adults are associated with stressful life events and negative memory bias. Atten Defic Hyperact Disord. 2018;10(2):151-60.

52.Vrijsen JN, Vogel S, Arias-Vásquez A, Franke B, Fernández G, Becker ES, et al. Depressed patients in remission show an interaction between variance in the mineralocorticoid receptor NR3C2 gene and childhood trauma on negative memory bias. Psychiatr Genet. 2015;25(3):99-105.

53.Groefsema M, Engels R, Kuntsche E, Smit K, Luijten M. Cognitive biases for social alcohol-related pictures and alcohol use in specific social settings: an event-level study. Alcohol Clin Exp Res. 2016;40(9):2001-10.

54.Groefsema MM, Mies GW, Cousijn J, Engels R, Sescousse G, Luijten M. Brain responses and approach bias to social alcohol cues and their association with drinking in a social setting in young adult males. Eur J Neurosci. 2020;51(6):1491-503.

55.Figner B, Weber E. Who takes risks when and why?: Determinants of risk taking. Current Directions in Psychological Science. 2011;20:211-6.

56.Figner B, Mackinlay RJ, Wilkening F, Weber EU. Affective and deliberative processes in risky choice: age differences in risk taking in the Columbia Card Task. J Exp Psychol Learn Mem Cogn. 2009;35(3):709-30.

57.Becker GM, DeGroot MH, Marschak J. Measuring utility by a single-response sequential method. Behav Sci. 1964;9(3):226-32.

58.Veling H, Chen Z, Tombrock MC, Verpaalen IAM, Schmitz LI, Dijksterhuis A, et al. Training impulsive choices for healthy and sustainable food. J Exp Psychol Appl. 2017;23(2):204-15.

59.Schonberg T, Bakkour A, Hover AM, Mumford JA, Nagar L, Perez J, et al. Changing value through cued approach: an automatic mechanism of behavior change. Nat Neurosci. 2014;17(4):625-30.

60.Hergueux J, Jacquemet N. Social preferences in the online laboratory: a randomized experiment. Experimental Economics. 2015;18(2):251-83.

61.Kerschbamer R. The geometry of distributional preferences and a non-parametric identification approach: The Equality Equivalence Test. European Economic Review. 2015;76:85-103.

62.Kerschbamer R, Müller D. Social preferences and political attitudes: An online experiment on a large heterogeneous sample. Journal of Public Economics. 2020;182:104076.

63.Noussair CN, Trautmann ST, van de Kuilen G. Higher order risk attitudes, demographics, and financial decisions. The Review of Economic Studies. 2014;81(1):325-55.

64.Falk A, Becker A, Dohmen T, Enke B, Huffman D, Sunde U. Global evidence on economic preferences. The Quarterly Journal of Economics. 2018;133(4):1645-92.

65.Dimmock SG, Kouwenberg R, Wakker PP. Ambiguity attitudes in a large representative sample. Management Science. 2016;62(5):1363-80.

66.Kuijpers M, Hakemulder F, Tan E, Doicaru M. Exploring absorbing reading experiences: Developing and validating a self-report scale to measure story world absorption. Scientific Study of Literature. 2014;4.

67.Mak M, Willems R. Mental simulation during literary reading: Individual differences revealed with eye-tracking. Language, Cognition and Neuroscience. 2018;34:1-25.

68.Faber M, Mak M, Willems R. Word skipping as an indicator of individual reading style during literary reading. Journal of Eye Movement Research. 2020;13.

69.Knoop C, Wagner V, Jacobsen T, Menninghaus W. Mapping the aesthetic space of literature "from below". Poetics. 2016;56.

70.Hartung F, Hagoort P, Willems RM. Readers select a comprehension mode independent of pronoun: Evidence from fMRI during narrative comprehension. Brain Lang. 2017;170:29-38.

71.Jasso G, Opp KD. Probing the character of norms: A factorial survey analysis of the norms of political action. American Sociological Review. 1997;62:947.

72.Rooks G, Raub W, Selten R, Tazelaar F. How inter-firm co-operation depends on social embeddedness: A vignette study. Acta Sociologica. 2000;43(2):123-37.

73.Keuleers E, Brysbaert M, New B. SUBTLEX-NL: A new measure for Dutch word frequency based on film subtitles. Behavior Research Methods. 2010;42(3):643-50.

74.Keuleers E, Stevens M, Mandera P, Brysbaert M. Word knowledge in the crowd: Measuring vocabulary size and word prevalence in a massive online experiment. Quarterly journal of experimental psychology (2006). 2015;68:1-62.

75.Leek MR. Adaptive procedures in psychophysical research. Perception & Psychophysics. 2001;63(8):1279-92.

76.Dunn LM, Dunn D. Peabody Picture Vocabulary Test (3rd Edition). Circle Pines: American Guidance Service. 1997.

77.Schlichting L. Peabody Picture Vocabulary Test Dutch-III-NL. Amsterdam, NL: Harcourt Assessment BV. 2005.

78.Brysbaert M, Sui L, Dirix N, Hintz F. Dutch Author Recognition Test. J Cogn. 2020;3(1):6-.

79.Hubers F, Snijders T, Hoop H. How the brain processes violations of the grammatical norm: An fMRI study. Brain and Language. 2016;163:22-31.

80.Hintz F, Jongman SR, Dijkhuis M, van ’t Hoff V, McQueen JM, Meyer AS. Shared lexical access processes in speaking and listening? An individual differences study. Journal of Experimental Psychology: Learning, Memory, and Cognition. 2020;46(6):1048–63.

81.Salthouse TA. The processing-speed theory of adult age differences in cognition. Psychol Rev. 1996;103(3):403-28.

82.Earles JL, Salthouse TA. Interrelations of age, health, and speed. J Gerontol B Psychol Sci Soc Sci. 1995;50(1):P33-p41.

83.Huettig F, Janse E. Individual differences in working memory and processing speed predict anticipatory spoken language processing in the visual world. Language, Cognition and Neuroscience. 2016;31(1):80-93.

84.Deary IJ, Liewald D, Nissan J. A free, easy-to-use, computer-based simple and four-choice reaction time programme: the Deary-Liewald reaction time task. Behav Res Methods. 2011;43(1):258-68.

85.Wechsler D. WAIS-III (3rd edition). Amsterdam: Harcourt Test Publishers. 2004.

86.Kessels RP, van den Berg E, Ruis C, Brands AM. The backward span of the Corsi Block-Tapping Task and its association with the WAIS-III Digit Span. Assessment. 2008;15(4):426-34.

87.Chu M, Meyer A, Foulkes L, Kita S. Individual differences in frequency and saliency of speech-accompanying gestures: the role of cognitive abilities and empathy. J Exp Psychol Gen. 2014;143(2):694-709.

88.Berch DB, Krikorian R, Huha EM. The Corsi block-tapping task: methodological and theoretical considerations. Brain Cogn. 1998;38(3):317-38.

89.Raven J, Raven JC, Court JH. Raven manual section 4: advanced progressive matrices. Oxford, UK: Oxford Psychologists Press. 1998.

90.de Groot F, Koelewijn T, Huettig F, Olivers C. A stimulus set of words and pictures matched for visual and semantic similarity. Journal of Cognitive Psychology. 2015;28:1-15.

91.Marian V, Bartolotti J, Chabal S, Shook A. CLEARPOND: cross-linguistic easy-access resource for phonological and orthographic neighborhood densities. PLoS One. 2012;7(8):e43230.

92.Shao Z, Janse E, Visser K, Meyer AS. What do verbal fluency tasks measure? Predictors of verbal fluency performance in older adults. Frontiers in psychology. 2014;5:772-.

93.Mainz N, Shao Z, Brysbaert M, Meyer AS. Vocabulary knowledge predicts lexical processing: Evidence from a group of participants with diverse educational backgrounds. Front Psychol. 2017;8:1164.

94.Menenti L, Petersson KM, Hagoort P. From reference to sense: how the brain encodes meaning for speaking. Front Psychol. 2011;2:384.

95.Jacobs CL, Cho S-J, Watson DG. Self-priming in production: Evidence for a hybrid model of syntactic priming. Cognitive Science. 2019;43(7):e12749.

96.Konopka AE, Meyer A, Forest TA. Planning to speak in L1 and L2. Cognitive Psychology. 2018;102:72-104.

97.Jongman S, Khoe Y, Hintz F. Vocabulary size influences spontaneous speech in native language users: validating the use of automatic speech recognition in individual differences research. Language and Speech. 2020:002383092091107.

98.Keuleers E, Brysbaert M. Wuggy: a multilingual pseudoword generator. Behav Res Methods. 2010;42(3):627-33.

99.Boersma PPG. Praat, a system for doing phonetics by computer (Version 5.1.19) [Computer program]. 2001.

100.McQueen JM. Rhyme decisions to spoken words and nonwords. Mem Cognit. 1993;21(2):210-22.

101.Piai V, Roelofs A, Rommers J, Maris E. Beta oscillations reflect memory and motor aspects of spoken word production. Hum Brain Mapp. 2015;36(7):2767-80.

102.Snodgrass JG, Vanderwart M. A standardized set of 260 pictures: norms for name agreement, image agreement, familiarity, and visual complexity. J Exp Psychol Hum Learn. 1980;6(2):174-215.

103.Altmann GTM, Kamide Y. Incremental interpretation at verbs: restricting the domain of subsequent reference. Cognition. 1999;73(3):247-64.

104.Hintz F, Meyer AS, Huettig F. Predictors of verb-mediated anticipatory eye movements in the visual world. J Exp Psychol Learn Mem Cogn. 2017;43(9):1352-74.

105.Schoffelen J-M, Oostenveld R, Lam NHL, Uddén J, Hultén A, Hagoort P. A 204-subject multimodal neuroimaging dataset to study language processing. Scientific Data. 2019;6(1):17.
