# Supplementary material for: Protocol of the Healthy Brain Study: An accessible resource for understanding the human brain and how it dynamically and individually operates in its bio-social context
Source: PLoS One. 2021 Dec 29;16(12):e0260952. doi: 10.1371/journal.pone.0260952 (PMC8716054; doi:10.1371/journal.pone.0260952)
Supplement: Attachment — Submitted filename: HBS Consortium_Rebuttal letter_PLOS ONE.docx [file pone.0260952.s003.docx]

Dear Dr. Amir-Homayoun Javadi,

Thank you for judging our manuscript entitled ‘Protocol of the Healthy Brain Study: an accessible resource for understanding the human brain and how it dynamically and individually operates in its bio-social context’ favorably. Herewith we would like to respond to the reviewer.

When we refer to line numbers in the following paragraphs, we refer to the line numbers in the revised manuscript with track changes.

Journal requirements

*1) Please ensure that your manuscript meets PLOS ONE's style requirements, including those for file naming.*

We have applied the PLOS ONE style templates.

*2) If there are ethical or legal restrictions on sharing a de-identified data set, please explain them in detail (e.g., data contain potentially sensitive information, data are owned by a third-party organization, etc.) and who has imposed them (e.g., an ethics committee). Please also provide contact information for a data access committee, ethics committee, or other institutional body to which data requests may be sent.*

Sharing the full Healthy Brain Study (HBS) resource does not comply with the EU General Data Protection Regulation, because the HBS resource contains a substantial amount of sensitive personal data.

Applications for data access can be sent to hbs-data@radboudumc.nl. Then, the HBS scientific board reviews the application. Our governance policy ensures that all use of data is within the limits of the original purpose as described in the information brochure for participants and that only those data are shared that are relevant for answering the proposed research question.

*3) Please amend your authorship list in your manuscript file to include author Lucy Overbeek.*

We would like to propose that the Healthy Brain Study consortium is the only author and omit the names of the project leader Guillén Fernández and project manager Lucy Overbeek. In our view, mentioning the consortium as the only author would perfectly reflect the nature of the team-science based cross-faculty initiative from the Radboud campus in Nijmegen. We consider our roles as supportive and required for managing the project rather than as principal investigators. We understand that a corresponding author is required. Guillén Fernández fulfills the role of the corresponding author on behalf of the consortium. As stated, all members of the consortium contributed to the design of the study, edited the manuscript, and approved the final version.

Response to reviewer

** I agree that a reductionist approach is common, but several longitudinal studies surely include a broad range of variables and a holistic approach; this is not unique for HBS.*

We fully agree with the reviewer that several longitudinal studies include a broad range of variables and a holistic approach. In lines 35-38 we mention that “we think that current brain research suffers from at least five key limitations and we set up the Healthy Brain Study (HBS) to tackle these five limitations together”. Thus, we consider the uniqueness of the HBS that it combines the following five strengths 1) a broad range of variables, 2) repeated assessments across one year, 3) single subject inferences, 4) real-world assessments, and 5) a population-based sample.

We propose stressing that tackling these five limitations together is the uniqueness of the HBS by adapting the sentence starting in line 94 into “In conclusion, the unique feature of the HBS is that it combines the five above-mentioned strength resulting in in-depth phenotyping of a large range of cognitive, affective, behavioral, and social dimensions with a biological sampling of brain and body-related processes.”.

However, there are other studies (e.g., Betula project) that have another focus on a longitudinal design with a very long-term perspective of more than 25 years (https://www.umu.se/en/research/projects/betula---aging-memory-and-dementia/).

** Life events are highlighted. Such events have been explored in several other past studies, and a question is whether the authors in the quite short period of 1 year expect a sufficient number of significant events to substantiate analyses? Here, the timing with the pandemic is very interesting and it is a strength of the study to capture this major event.*

When designing the study, we hoped for some major national events around for example governmental elections or sports. A pandemic was of course not expected, but is indeed very interesting and offers additional opportunities.

We agree with the reviewer that one year is a rather a short period for capturing life events, but we chose the age range of 30-39 because it is a socially challenging age range that is characterized by a relatively high number of rather impactful life events (e.g., family planning, career-related changes, buying a house etc.). To assess life events, participants give responses to twenty types of life events (Brugha & Cragg, 1990). In addition, to assess the subjective impact of life events, we ask participants to rate the subjective impact the event had on their lives on a scale from 0 (no impact at all) to 10 (major impact, fundamental change). Preliminary data showed that 130 out of 298 (44%) of the HBS participants that completed the life event questionnaire at T1 experienced one or more life events in the year before participating and 78 out of 175 (45%) and 46 out of 100 (46%) of HBS participants that completed the life event questionnaire at T2 respectively T3 experienced one or more life events. As this is a protocol paper, we do not want to report any preliminary data and propose not to include the above-mentioned data on life events in the manuscript.

Reference:

Brugha, T. S. & Cragg, D. The list of threatening experiences: the reliability and validity of a brief life events questionnaire. *Acta Psychiatr Scand* **82**, 77-81 (1990).

** The protocol has an elegant assessment of factors such as physical activity, stress, and sleep that go beyond past studies. However, to be fair, also past studies meant to target real world events (even if with scales and questionnaires).*

We agree with the reviewer that many past and ongoing studies assess real-world events (even if with scales and questionnaires). We consider our approach innovative, because for two reasons.

First, we believe that assessing real-world events with wearable devices is more objective than scales and questionnaires. When we designed the study in 2017, hardly any longitudinal study included wearable devices. As the field of wearable technology has developed rapidly, in the meantime, several longitudinal studies have added wearable devices to their data collection methods. For example, subsets of UK Biobank participants and Rotterdam Study participants wore an accelerometer (Doherty *et al.*, 2017; Koolhaas *et al.*, 2017). Furthermore, in line with our response to the first comment of the reviewer, we would like to stress that including real-world assessments is one of the five strengths of the HBS, so it is not unique by itself. In particular, the HBS includes repeated assessments with wearable devices in three different seasons over one year starting at varying time points within a year. When we designed the study, to our knowledge, this was unique for HBS. In the meantime, a subset of UK Biobank participants is performing seasonal repeats with a wearable device (<https://biobank.ctsu.ox.ac.uk/crystal/label.cgi?id=1008>).

Second, the HBS combines physiological recordings with wearable devices with ecological momentary assessments using a smartphone application. We consider the additional collection of momentary assessments of mood and behavior and context information innovative.

We added this explanation to the discussion section when reflecting on the HBS study design (line 393).

References:

Doherty A. *et al.* Large Scale Population Assessment of Physical Activity Using Wrist Worn Accelerometers: The UK Biobank Study. *PLoS One* **12(2),** e0169649 (2017).

Koolhaas C.M. *et al.* Objective Measures of Activity in the Elderly: Distribution and Associations With Demographic and Health Factors. *Journal of the American Medical Directors Association* **18 (10)**, 838-847 (2017).

** I applaud the stress on population-based cohorts and the risk with selection bias in convenience samples. Here, it could also be mentioned that studies like the Swedish Betula project (e.g., Nyberg, et al, 2020 Ageing Res Rev) have used such an approach. In HBS, some comment is warranted on the exclusion criteria in relation to representativeness (such as taking antidepressants or having a disease that hinders physical exercise). Relatedly, the strategies to recruit participants could influence the final representativeness of the sample. For example, the involvement of employers can bias the sample to people who have a work. Have the authors any plans of assessing how representative for the general population their final sample actually will be?*

We defined a population-based sample as 1,000 HBS participants (500 men and 500 women) from the Nijmegen region (≤ 15 km) of whom 220 have a low, 340 a middle, and 430 a high level of education. We perform continuous monitoring of sex and level of education for enrollment and drop-out rates. For example, when we observed that our first wave of participants consisted of mainly higher educated people, we adapted the campaign by displaying pictures and stories of real, middle educated participants in promotion materials and focused on employers with low and middle educated staff. Concerning recruiting unemployed people, we reach them by their municipalities, our social media campaign and local influencers.

** Opting out from a study and later dropping out from a study are important variables. So far, after a quite short period of time, as many as 13% withdrew (typically after the 1st assessment). I lacked information on whether the researchers will replace those who drop out at such an early stage, and also how they plan to deal with drop-out analyses.*

We aim to acquire full longitudinal datasets of 1,000 participants. We expect a withdrawal rate of 15%, and will therefore recruit 1,150 individuals to participate in the study. This information was lacking in the manuscript and has now been added to the recruitment paragraph in lines 138-139: “We aim to acquire full longitudinal datasets of 1,000 participants. We expect a withdrawal rate of 15%, and will therefore recruit 1,150 individuals to participate in the study.”.

How to deal with drop-out analyses is up to the researchers who will use the data. Partial datasets of withdrawn participants will be included in the data release, along with documentation of what parts of the data are available for each subject and information about the reasoning for withdrawal.

Other changes

- Line 242, Table 1a: fertility treatment and breastfeeding are not part of the questionnaire.
- Line 295: “At the end of December 2020, the HBS included 298 participants. Forty participants (13%) withdrew from the study so far, due to personal circumstances (e.g., pregnancy) or too much burden. Most participants withdrew after the first assessment. To date, participants performed 376 lab visits: 257 participants carried out the first assessment, 83 participants the second assessment, and 36 participants the third assessment completing the entire study protocol.” has been adapted into “At the end of June 2021, the HBS included 418 participants. Seventeen-one participants (17%) withdrew from the study so far, mostly because they experienced too much burden (n=41; 58%), got pregnant (n=11; 15%), or had been given a diagnosis or medication treatment (n=8; 11%). Most participants withdrew after the first assessment. To date, participants performed 755 lab visits: 380 participants carried out the first assessment, 237 participants the second assessment, and 138 participants the third assessment completing the entire study protocol.”
- Table 1a is renamed to Table 1, Table 1b to Table 7, Table 7 to Table 8.
- Reference list: references 27, 164, 165, 166 have been added.
- Link for data access is adapted to <https://www.healthybrainstudy.nl/en/data-and-methods/access> in line 27 and 431.

We hope we have satisfactorily responded to the comments and are looking forward to your decision about publishing our manuscript in PLOS ONE.

On behalf of the Healthy Brain Study consortium,

Guillén Fernández	Lucy Overbeek

Guillen.fernandez@donders.ru.nl	Lucy.overbeek@radboudumc.nl

Donders Institute for Brain, Cognition and Behavior	Radboud University Medical Center,

Radboud University Medical Center,	Nijmegen, The Netherlands

Nijmegen, The Netherlands
