# Supplementary material for: Correction: Protocol of the Healthy Brain Study: An accessible resource for understanding the human brain and how it dynamically and individually operates in its bio-social context
Source: PLoS One. 2022 Apr 11;17(4):e0267071. doi: 10.1371/journal.pone.0267071 (PMC9000123; doi:10.1371/journal.pone.0267071)
Supplement: S2 File — (PDF) [file pone.0267071.s002.pdf]

PLOS ONE

STUDY PROTOCOL

Protocol of the Healthy Brain Study: An accessible resource for understanding the human brain and how it dynamically and individually operates in its bio-social context

Healthy Brain Study consortium[1,2,3], Esther Aarts[4], Agnes Akkerman[5], Mareike Altgassen[6], Ronald Bartels[2], Debby Beckers[7], Kirsten Bevelander[2], Erik Bijleveld[7], Esmeralda Blaney Davidson[2], Annemarie Boleij[2], Janita Bralten[2], Toon Cillessen[7], Jurgen Claassen[2], Roshan Cools[8], Ineke Cornelissen[2], Martin Dresler[8], Thijs Eijsvogels[2], Myrthe Faber[8], Guillén Fernández[8]*, Bernd Figner[4,7], Matthias Fritsche[4], Sascha Füllbrunn[5], Surya Gayet[4], Marleen M. H. J. van Gelder[2], Marcel van Gerven[4], Sabine Geurts[7], Corina U. Greven[8], Martine Groefsema[7], Koen Haak[8], Peter Hagoort[3,4], Yvonne Hartman[2], Beatrice van der Heijden[5], Erno Hermans[8], Vivian Heuvelmans[2], Florian Hintz[3], Janet den Hollander[2], Anneloes M. Hulsman[4,7], Sebastian Idesis[9], Martin Jaeger[2], Esther Janse[10], Joost Janzing[2], Roy P. C. Kessels[4,8], Johan C. Karremans[7], Willemien de Kleijn[11], Marieke Klein[2], Floris Klumpers[4,7], Nils Kohn[8], Hubert Korzilius[5], Bas Krahmer[2], Floris de Lange[4], Judith van Leeuwen[8], Huaiyu Liu[7], Maartje Luijten[7], Peggy Manders[2], Katerina Manevska[5], José P. Marques[4], Jon Matthews[2], James M. McQueen[4], Pieter Medendorp[4], René Melis[2], Antje Meyer[3], Joukje Oosterman[4], Lucy Overbeek[2], Marius Peelen[4], Jean Popma[12], Geert Postma[13], Karin Roelofs[4,7], Yvonne G. T. van Rossenberg[5], Gabi Schaap[7], Paul Scheepers[2], Luc Selen[4], Marianne Starren[10], Dorine W. Swinkels[2], Indira Tendolkar[8], Dick Thijssen[2], Hans Timmerman[14], Rayyan Tutunji[8], Anil Tuladhar[8], Harm Veling[7], Maaike Verhagen[7], Jasper Verkroost[2], Jacqueline Vink[7], Vivian Vriezekolk[2], Janna Vrijsen[8], Jana Vyrastekova[5], Selina van der Wal[2], Roel Willems[4,10], Arthur Willemsen[2]

1 Radboud University, Nijmegen, The Netherlands, **2** Radboud University Medical Center, Nijmegen, The Netherlands, **3** Max Planck Institute for Psycholinguistics, Nijmegen, The Netherlands, **4** Donders Institute for Brain, Cognition and Behavior, Radboud University, Nijmegen, The Netherlands, **5** Institute for Management Research, Radboud University, Nijmegen, The Netherlands, **6** Johannes Gutenberg-University Mainz, Germany, **7** Behavioural Science Institute, Radboud University, Nijmegen, The Netherlands, **8** Donders Institute for Brain, Cognition and Behavior, Radboud University Medical Center, Nijmegen, The Netherlands, **9** Center for Brain and Cognition, University Pompeu Fabra, Barcelona, Spain, **10** Centre for Language Studies, Radboud University, Nijmegen, The Netherlands, **11** School of Psychology and Artificial Intelligence, Radboud University, Nijmegen, The Netherlands, **12** Interdisciplinary Hub for Security, Privacy and Data Governance, Radboud University, Nijmegen, The Netherlands, **13** Institute for Molecules and Materials, Radboud University, Nijmegen, The Netherlands, **14** University Medical Center Groningen, Groningen, The Netherlands

* Guillen.fernandez@donders.ru.nl

OPEN ACCESS

Citation: Healthy Brain Study consortium, Aarts E, Akkerman A, Altgassen M, Bartels R, Beckers D, et al. (2021) Protocol of the Healthy Brain Study: An accessible resource for understanding the human brain and how it dynamically and individually operates in its bio-social context. PLoS ONE 16(12): e0260952. https://doi.org/10.1371/journal.pone.0260952

Editor: Fulvio D'Acquisto, University of Roehampton - Whitelands College, UNITED KINGDOM

Received: May 12, 2021

Accepted: November 20, 2021

Published: December 29, 2021

Peer Review History: PLOS recognizes the benefits of transparency in the peer review process; therefore, we enable the publication of all of the content of peer review and author responses alongside final, published articles. The editorial history of this article is available here: https://doi.org/10.1371/journal.pone.0260952

Copyright: © 2021 Aarts et al. This is an open access article distributed under the terms of the Creative Commons Attribution License, which permits unrestricted use, distribution, and reproduction in any medium, provided the original author and source are credited.

Abstract

The endeavor to understand the human brain has seen more progress in the last few decades than in the previous two millennia. Still, our understanding of how the human brain relates to behavior in the real world and how this link is modulated by biological, social, and environmental factors is limited. To address this, we designed the Healthy Brain Study (HBS), an interdisciplinary, longitudinal, cohort study based on multidimensional, dynamic assessments in both the laboratory and the real world. Here, we describe the rationale and

Funding: The HBS is funded by the Reinier Post Foundation and Radboud University, Nijmegen, the Netherlands. The funders had and will not have a role in study design, data collection and analysis, decision to publish, or preparation of the manuscript.

Competing interests: The authors have declared that no competing interests exist.

design of the currently ongoing HBS. The HBS is examining a population-based sample of 1,000 healthy participants (age 30–39) who are thoroughly studied across an entire year. Data are collected through cognitive, affective, behavioral, and physiological testing, neuro-imaging, bio-sampling, questionnaires, ecological momentary assessment, and real-world assessments using wearable devices. These data will become an accessible resource for the scientific community enabling the next step in understanding the human brain and how it dynamically and individually operates in its bio-social context. An access procedure to the collected data and bio-samples is in place and published on https://www.healthybrainstudy.nl/en/data-and-methods/access.

Trail registration: https://www.trialregister.nl/trial/7955.

Introduction

The human brain is seen as civilization's most precious resource [1], both creating and interacting with our increasingly complex environment, it enables us to be conscious and social human beings. Brain functioning also plays a pivotal role in major societal challenges such as health, demographic change, and well-being. Due to developments in different scientific fields, the endeavor to understand the human brain has seen more progress in the last few decades than in the two millennia before. However, we think that current brain research suffers from at least five key limitations and we set up the Healthy Brain Study (HBS) to tackle these five limitations together and, thereby, to facilitate our understanding of how the human brain relates to behavior in the real world and how this link is modulated by biological, social, and environmental factors. In the following paragraphs, we explain the five main design choices of the HBS.

Firstly, a reductionist approach–in which researchers try to understand reality by focusing on a limited number of variables–has been understandably popular as it is vital to obtain detailed mechanistic insights. However, complex dynamical systems, like the human brain, cannot be properly understood by focusing on just one aspect at a time [2–4]. Human brain functioning includes enabling consciousness and cognition, generating emotions, and producing adaptive behavior, and it performs all of these functions while embedded in its biological and social (bio-social) environment [5]. To enable researchers to understand the complexity of human brain functioning in its bio-social context, the HBS provides a broad range of variables within a holistic approach.

Secondly, the brain's operations cannot be fully understood by single assessments obtained at a specific point in time, but require repeated measurements or continuous monitoring. Single-session assessments may be sufficient to uncover stable traits or processes. However, they do not capture changes in brain functioning that constitute a core feature of our plastic and adaptive brain [6, 7]. Similarly, the body and the social environment are subject to change. For example, seasonality is observed in affect [8, 9], behavior [9, 10], and biological [11–14] and social [9] factors. Most of the studies mentioned were cross-sectional and explicitly stress the need for longitudinal studies that assess within-subject variation. Therefore, in the HBS, participants perform repeated assessments in three different seasons over one year starting at varying time points within a year. Thereby, we aim to reliably and validly capture changes in human brain operations that may be related not only to seasonality, but also to relevant life events and incidental or dynamic changes in biological factors (e.g., inflammation markers), social factors (e.g., household composition, work relations, friendships, politics, media exposure, lockdown), and environmental factors (e.g., daylight hours, exposure to chemicals).

Thirdly, group averages are critical in revealing general principles, but they gloss over differences that make us individual human beings. The human brain is arguably the most individual organ we have and is shaped by our experiences throughout life. Therefore, a large and rich sample is required before single subject inferences can be made about underlying principles of diversity in cognition, affect, and behavior [15, 16]. Given this, the HBS aims to include a broad range of repeated assessments of 1,000 participants.

Fourthly, laboratory assessments enable well-controlled analyses, but they may show low ecological validity in generalizing cognition, affect, and behavior to real-world settings. To understand cognition, affect, and behavior more comprehensively, there is a need for assessments both in the laboratory as well as in the real world [17, 18]. In the HBS, we perform a real-world assessment of physical activity, stress, and sleep with validated wearable devices. Furthermore, we apply ecological momentary assessments using a smartphone application. Taken together, these assessments enable us to understand cognition, affect, and behavior in the context where they naturally occur.

Finally, a healthy volunteer selection bias is a frequent problem in both cohort studies and neuroscience studies. For example, UK Biobank participants were more likely to be female, have a healthy lifestyle, and live in less socioeconomically deprived areas compared to the general population [19]. Also, students, the usual participants in cognitive neuroscience studies, function well, are often relatively healthy and have a high socioeconomic status [20]. Also, most population-based cohorts and large-scale studies include either developing populations [21–23] or advanced aging populations [24–27]. Therefore, the HBS includes a broad population-based sample of individuals who are 30–39 years old that reflects the general population in terms of gender and educational attainment. The age range was chosen to represent adults beyond the age of developmental brain changes and before the onset of brain changes due to advanced aging or neurodegenerative disease. The lower limit of 30 years excludes any neurodevelopment effect as the brain has matured by this point [28]. Also, 30–39 is a socially challenging age range because it is generally characterized by a relatively high number of rather impactful life events (e.g., family planning, career-related changes, buying a house).

In conclusion, the unique feature of the HBS is that it combines the five above-mentioned strengths resulting in in-depth phenotyping of a large range of cognitive, affective, behavioral, and social dimensions with a biological sampling of brain and body-related processes. This enables the extraction of a detailed bio-social fingerprint for the participants in the cohort. Such a detailed fingerprint is currently not available. The availability of HBS will contribute to a better understanding of risks and potentials in behavior in the real world at the individual level. This paper describes the rationale and design of the currently ongoing HBS, which originated from an interdisciplinary, team science [29] based cross-faculty initiative from the Radboud campus in Nijmegen, the Netherlands, including Radboud University, Radboud University Medical Center, and the Max Planck Institute for Psycholinguistics.

Methods/Design

Study design and setting

The HBS is a longitudinal cohort study in both laboratory and real-world settings. All laboratory assessments take place at a single-center on Radboud campus, Nijmegen, the Netherlands.

Participants

The HBS aims to include 1,000 participants (500 men and 500 women) from the Nijmegen region ($\leq$ 15 km) of whom 220 have a low, 340 a middle, and 430 a high level of education. Nijmegen is a medium-sized city in the east of the Netherlands with 176,731 citizens on the 1st

of January 2019 of whom 74% are native Dutch, which is comparable to the overall proportion of native Dutch citizens of the Netherlands (76%) [30]. In contrast, large cities (> 500,000 citizens) in the west of the Netherlands like Amsterdam, Rotterdam, and The Hague have respectively 46%, 48%, 45% native Dutch citizens [30]. Regarding educational attainment, 22% of Nijmegen citizens are primary and secondary educated (low level), 34% are primary, secondary, and vocationally educated (middle level), and 43% of the population have also a university degree (high level). Nijmegen has less citizens with low and middle level of education and more citizens with high level of education compared to the overall proportions of Dutch citizens (28%, 41%, 30% of citizens have respectively low, middle, and high level of education) [30]. In comparison, some large cities in the Netherlands have a higher proportion of citizens with a high level of education (e.g., Amsterdam 48%, Utrecht 52%), while other large cities have a higher proportion of citizens with a low level of education (e.g., Rotterdam 32%, The Hague 31%) [30].

Inclusion criteria are age 30–39 years, living in the Nijmegen region (≤ 15km; because of feasibility), willingness, and ability to follow the study protocol. Exclusion criteria are: not speaking, reading, and/or understanding the Dutch language (minimum B1 level), a prior history of significant psychiatric or neurological illness (self-report), a current disease that affects the brain, a current medication that is therapeutically targeted at the brain (e.g., antidepressants, methylphenidate), pregnancy, contra-indication for MRI (metal or devices in the upper body (cardiac pacemaker, cochlear implant, aneurism clip), previous brain surgery, moderate to severe claustrophobia), contra-indication for the submaximal Åstrand cycle test (current use of beta-blockers, a current disease that hinders physical exercise), contra-indication for the cold pressor test (Raynaud's phenomenon, chronic pain syndrome in shoulder or arm, open wounds on arm or hand, scleroderma, arteriovenous fistula or shunt, presence of (unstable) angina pectoris).

Recruitment

We aim to acquire full longitudinal datasets of 1,000 participants. We expect a withdrawal rate of 15%, and will therefore recruit 1,150 individuals to participate in the study. We apply different strategies to recruit participants. Firstly, municipalities, general practitioners, and employers of different sectors based in the Nijmegen region send the HBS invitation and research flyer to their citizens, clients, and employees, respectively. Employers are asked to sponsor the study by (partly) exempting their employees from three working days which allows them to participate in three lab visits. Employees remain entirely free to decide whether or not they want to participate. Also, campaigns to increase awareness of the HBS have been launched.

Potential participants fill out contact details in an online form on the website https://www.healthybrainstudy.nl and receive the study brochure. Participants can watch short videos on the website that explain the various tests and assessments or learn about the experiences of an HBS participant. Participants are contacted via phone and invited to a face-to-face information meeting on the Radboud campus. Participants provide written informed consent at this meeting before participation.

Ethics

The Institutional Review Board of Radboud University Medical Center approved the HBS on the 23rd of May, 2019 (reference number: 2018–4894) in accordance with the latest revision of the Declaration of Helsinki [31]. Incidental findings could occur both while conducting the study (e.g., observed during assessments) and while using the data and biosamples in the future to answer research questions. If a researcher or research assistant notices a potential

finding incidentally, he/she will contact the principal investigator, who approaches an incidental findings committee. At the Radboud Campus, such committees exist for neuroimaging and genetics. For other findings, the principal investigator contacts a medical doctor with relevant expertise. If, according to the committee or medical expert, no clinically relevant finding is identified, the participant remains uninformed. In all other cases, the participant's general practitioner is sent a letter describing the findings. At the same time, the participant receives a request to contact their general practitioner. Participants must consent to this procedure and provide the contact details of their general practitioner, otherwise, they are not allowed to participate.

Participant panel, feedback of participants, incentives, and citizen science

A participant panel consisting of twelve people (age 30–39, 6 women and 6 men) was set up to aid in the design of the study. The panel advises on communication materials and incentives. For example, the panel gives feedback on the website, study information, posters, and flyers. Moreover, the first 243 participants filled out a questionnaire on their experience of the first lab visit, which provided us with feedback on the study procedures and on keeping participants involved. For example, we developed an online dashboard, because participants indicated that they would prefer more individual feedback on results. Participants receive gadgets after each assessment, and we organize (online) participant events. After completion of the study protocol, participants receive €150 with a maximal addition of €50 for assessment specific incentives.

Besides, a citizen science platform is used to involve participants as well as other citizens in generating research topics and questions that can be investigated with the HBS resource [32]. We 'crowdsource' lists of research topics and/or research questions that participants and citizens think are useful for examining with the HBS resource. At the same time, they also rate the importance of the crowd-generated suggestions by other participants and citizens resulting in an overview that reflects the relevance and prioritization of their overall input.

Quality management and safety

Research assistants and nurses received extensive training for the assessments undertaken as part of the study protocol. We adapted existing standardized operating procedures (SOPs) if available and developed a new SOP otherwise. An independent study monitor annually performs checks to ensure that the study protocol is followed.

Data management and data availability

We use Ldot [33], which is a web application that only stores personal and logistical data, for communication with our participants. For data acquisition, we use Castor EDC [34] to provide electronic case report forms and online questionnaires. In addition, a smartphone application for ecological momentary assessments was developed. After participants have performed the real-world assessments, our data managers extract the raw data that is stored locally on the device. Bio-samples are stored at the Radboud Biobank with their sample tracking system, sample processing SOPs, and standardized sample storage conditions being employed [35]. Furthermore, a Polymorphic Encryption and Pseudonymization (PEP) infrastructure was developed for the HBS to protect all data streams and the privacy of participants [36, 37] (Fig 1). Ldot, Castor EDC, the smartphone application, and PEP meet the requirements of the European General Data Protection Regulation.

For each participant, the PEP-system generates unique pseudonyms for the different assessments to avoid the coupling of data to an individual participant during the data collection

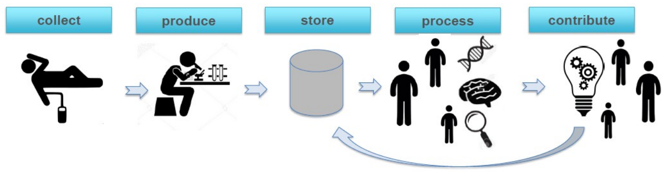

Who	Assessor	Producer	Repository	Researcher
**Identity information**	Identifiable	Pseudonymised	Encrypted/ Pseudonymised	Pseudonymised
**Data**	Partly Clear text	Clear Text	Encrypted	Clear text
**Linkability**	Linkable to person	Unlinkable	Unlinkable	Linkable to pseudonym

Fig 1. The Polymorphic Encryption and Pseudonymization (PEP) infrastructure.

https://doi.org/10.1371/journal.pone.0260952.g001

phase (step 1: collect). A backup of the data is stored locally (step 2: produce) and a copy is encrypted and transferred to the data repository (step 3: store). In the same step, the data are cryptographically pseudonymized. The data can only be decrypted in the processing environment where scientific analyses are performed (step 4: process). The PEP method ensures that different datasets obtained from the repository cannot be linked easily by different research projects because pseudonyms identifying a single participant are personalized at the project level, and data transfer can be minimized based on researchers' requirements. Derived data, produced by researchers, can be stored in the data repository (step 5: contribute) for future use by other researchers using their researcher-specific pseudonyms.

The PEP-system was created to deal with the rigidity of the traditional encryption/decryption process by using polymorphic encryption. PEP ensures that there is no need to a priori fix the encryption key for the data. The PEP system enables different research teams to have access to the entire dataset or only a subset (of participants and variables) of the data source with a specific, personalized decryption key. Due to its additional security, the PEP system is an ideal approach to store, manage, and share sensitive personal data in a research data repository that reduces the risk of a participant's privacy being violated.

Measures

The following paragraphs describe the measures briefly, while the supplementary information provides detailed descriptions (S1 File). Each assessment starts with pre-visit online questionnaires, followed by a burst week of real-world assessments, followed by a whole day lab visit, which in turn is followed by post-visit online questionnaires and assessments (Fig 2). Only those constructs that may be sensitive to change during one year (states) are repeated during the second and third assessments. The stable (trait) measures are equally distributed over the three assessments. The majority of measures are validated in prior research.

Pre-visit online questionnaires

Participants fill out questionnaires before the start of the burst week to assess baseline characteristics. The questionnaires cover general demographic questions and questions about lifestyle and well-being (Table 1).

Burst week with real-world assessments

The burst week consists of a real-world assessment of physical activity, stress, and sleep using validated wearable devices (Table 2) and ecological momentary assessments (EMA) using a

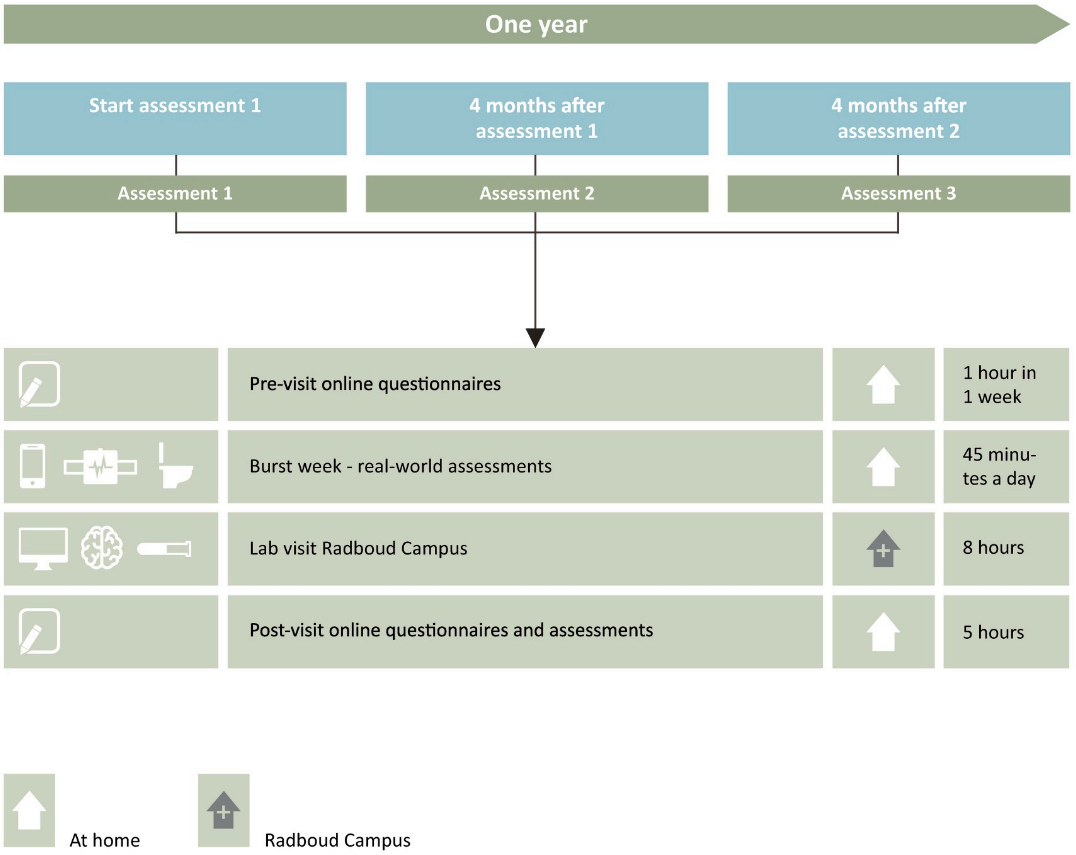

Fig 2. Design of data collection in the healthy brain study.

https://doi.org/10.1371/journal.pone.0260952.g002

smartphone application. The questionnaire for EMA covers mood, social company, online social interactions, context, control items, retrospection, anticipation, and substance use. In addition, participants perform the home collection of stool, urine, saliva, and diffusive sampling of chemicals using silicone wristbands during the burst week (Table 3).

Lab visit Radboud campus

Each eight-hour lab visit includes bio-sampling (Table 3), neuroimaging (Table 4), physiological (Table 2), cognitive (Table 5), affective (Table 5), behavioral (Table 5), and sensory assessments (Table 6). To avoid systematic carry-over and fatigue effects, the order of assessments varies between and within participants except for fasting blood sampling and blood pressure at the start of the day.

Post-visit online questionnaires and assessments

Participants fill out an online questionnaire assessing (mental) health, life events, social/relationships, work, politics, personality, and literacy after each lab visit (Table 7). Also, participants perform several online assessments about decision-making, narrative reading, and solidarity (Table 8). After their third and final lab visit, participants are invited to complete the 'Individual Differences in Language Skills' test battery (Table 9) assessing participants' linguistic knowledge, as well as linguistic processing and general cognitive skills.

Table 1. Pre-visit online questionnaires.

Domain	Name of the questionnaire	What does it measure?	Duration (minutes)	Assessment 1	Assessment 2	Assessment 3	Ref
**General information**	Demographic and socio-economic background	Demographic data, the highest level of education, income, household composition	10	x	x	x	[38]
	Pregnancy	Number of pregnancies, time to pregnancy, pregnancy outcome, hormones (anticonception), current child wish	3	x			
	Menstrual cycle	Menstrual cycle	1	x	x	x	
**Lifestyle**	Smoking history	Past behavior, age of onset	1	x			
	Smoking	Current behavior, frequency, and quantity	1	x	x	x	
	Fagerstrom Test of Nicotine Dependence (FTND)	Nicotine dependence (for current or ever smokers)	2	x	x	x	[39]
	Alcohol	Frequency and quantity in the last month, age of onset of alcohol use, binge drinking	2	x	x	x	
	Alcohol Use Disorder Identification Test (AUDIT)	Heavy alcohol use and associated problems	3	x	x	x	[40]
	Substance matrix Mate-q	Amount and frequency of substance use	5	x	x	x	[41]
	Food Frequency Questionnaire (FFQ)	Quantitative food intake	45	x			[42–45]
	Sedentary Behavior Questionnaire (SBQ)	Sedentary behavior in various domains (e.g. home, work, transportation)	5	x	x	x	[46]
	Pittsburgh Sleep Quality Index (PSQI)	Sleep quality	5	x	x	x	[47]
	Dream Recall Frequency Scale (DRFS)	Dream recall	1	x	x	x	[48]
	The Internet Gaming Disorder Scale	Problematic gaming	2	x	x	x	[49]
	The Social Media Disorder Scale	Problematic social media use	2	x	x	x	[50]
	Short Media Multitasking Measure (S-MMM)	Use of different media simultaneously	1	x	x	x	[51]
**Well-being**	Satisfaction with life scale	Well-being	2	x	x	x	[52]
	Cantril ladder	Well-being	1	x	x	x	[53]
	Five Facet Mindfulness Questionnaire–Short Form (FFMQ)	Mindfulness	10	x	x	x	[54]

https://doi.org/10.1371/journal.pone.0260952.t001

COVID-19 questionnaire

From March until July 2020, when the assessment of participants was not allowed due to the COVID-19 measures, the included participants at that point (N = 158) received a monthly questionnaire addressing behavior and worries regarding COVID-19, currently experienced anxiety [94], stress [95], and well-being [53]. Moreover, loneliness [102], sedentary behavior [46], and sleep quality [47] were assessed. We used the same questionnaires as we use in the three repeated assessments (Tables 1 and 7).

Results—Progress so far

Fig 3 presents the progress and milestones of the Healthy Brain Study. The first participant was included on the 9th of September, 2019.

At the end of June 2021, the HBS included 418 participants. Seventeen-one participants (17%) withdrew from the study so far, mostly because they experienced too much burden

Table 2. Physiological assessments.

Domain	Measure	Location	Assessment 1	Assessment 2	Assessment 3	Ref
**Physical activity**	Fitness	Campus	x	x	x	[55]
	Sedentary behavior	Home[1]	x	x	x	[56, 57]
**Stress**	Heart rate	Campus	x	x	x	
		Home[1]	x	x	x	[58]
	Heart rate variability	Home[1]	x	x	x	[58]
	Skin conductance	Home[1]	x	x	x	[58]
	Skin temperature	Home[1]	x	x	x	[58]
	Startle eye-blink	Campus	x	x	x	[59]
	Subjective stress levels	Campus	x	x	x	[60]
		Home[2]	x	x	x	
**Sleep**	Sleep duration	Home[1]	x	x	x	
	Sleep stages	Home[1]	x	x	x	
**Body composition**	Weight	Campus	x	x	x	
	Height	Campus	x	x	x	
	Waist-hip circumference	Campus	x	x	x	
	Body fat	Campus	x	x	x	[61]
	Fat weight	Campus	x	x	x	
	Total body water	Campus	x	x	x	
	Skeletal muscle mass	Campus	x	x	x	
	Body fat mass index	Campus	x	x	x	
	Fat-free mass index	Campus	x	x	x	
**Pain**	Subjective pain levels	Campus	x	x	x	[60, 62]
		Home[1]	x	x	x	
	Electrical pain thresholds	Campus	x	x	x	[63, 64]
**Cardiovascular**	Blood pressure	Campus	x	x	x	[65]
	Carotid artery reactivity	Campus	x	x	x	[66]

[1] By wearable device,

[2] By ecological momentary assessment (EMA).

https://doi.org/10.1371/journal.pone.0260952.t002

(n = 41; 58%), got pregnant (n = 11; 15%), or had been given a diagnosis or medication treatment (n = 8; 11%). Most participants withdrew after the first assessment. To date, participants performed 755 lab visits: 380 participants carried out the first assessment, 237 participants the second assessment, and 138 participants the third assessment completing the entire study protocol.

The COVID-19 pandemic interrupted the data acquisition phase. At that point, the HBS included 158 participants. Due to the lockdown, we canceled all assessments involving physical interaction as of the 16th of March, 2020. The HBS resumed participant assessments on the 15th of July, 2020 in compliance with the directives in force in the Netherlands. As a result, some participants (48%) have more than four months between repeated assessments. Besides, some participants (10%) have a delay between the burst week with real-world assessments and the lab visit at the Radboud campus.

Discussion

This paper presents the design of the currently ongoing HBS, which will result in a unique and accessible resource for the scientific community and its public and private partners. Data are

Table 3. Bio-samples and silicone wristband.

Bio-sample	Measure	Location	Assessment 1	Assessment 2	Assessment 3	Ref
**Stool**	Gut microbiome	Home	x	x	x	[12, 35, 67]
**Urine** (first morning)	Ions, such as calcium, potassium, sodium, magnesium	Home	x	x	x	[35, 68]
**Saliva**	Cortisol levels (short term; two baseline samples)	Home	x	x	x	[69]
	Cortisol levels (short term; before, immediately after, and 20 minutes after acute challenge)	Campus	x	x	x	
**Blood—EDTA plasma**	DNA	Campus	6 ml*			[35]
**Blood—PAX gene**	RNA	Campus	3x 2,5 ml*	3x 2,5 ml*	3x 2,5 ml*	
**Blood—EDTA plasma**	Future analyses	Campus	4x 10 ml*	4x 10 ml*	4x 10 ml*	
			1x 3 ml*	1x 3 ml*	1x 3 ml*	
**Blood—serum**	Future analyses (e.g., antibodies, proteomics)	Campus	10 ml*	10 ml*	10 ml*	
**Blood—heparin plasma**	Future analyses (e.g., hormones, metabolomics)	Campus	2x 10 ml*	2x 10 ml*	2x 10 ml*	
**Hair**	Cortisol levels (long term)	Campus	x	x	x	[70]
**Silicone wristband**	Exposure to chemicals in the surrounding environment	Home	x	x	x	[71, 72]

*The indicated volumes refer to whole blood volumes.

https://doi.org/10.1371/journal.pone.0260952.t003

collected through cognitive, affective, behavioral, and physiological testing, neuroimaging, bio-sampling, questionnaires, ecological momentary assessment, and real-world assessment using wearable devices. We believe that the HBS complements other studies–small and large–, which together enable the scientific community to take the next step in understanding the human brain and how it dynamically and individually operates in its bio-social context. Here, we present examples of research opportunities including citizen science, reflect on the HBS design choices and study population, and discuss our data security system which enables future data sharing.

Table 4. Neuroimaging at the campus.

Scan	Description	Duration (minutes)	Assessment 1	Assessment 2	Assessment 3	Ref
**Dummy scanner**		10	x			
**T1w 3D MPRAGE**	Anatomical scan	5	x	x	x	
**rfMRI**	Resting-state functional scan followed by resting-state questionnaire	10	x	x	x	[73, 74]
**mfMRI**	Movie functional scan	4,5	x	x	x	
**Scout, fieldmap, single-band reference EPIs**	Auxiliary scans	2	x	x	x	
**Diffusion-weighted imaging scan**	Structural connectivity characterizations and white matter tissue microstructural modelling	10	x			
**High-resolution T1w 3D MP2RAGE anatomical scan**	Quantitative T1 and cortical myelin mapping	10		x		[75]
**High-resolution T2*w scan**	Quantitative T2* and magnetic susceptibility mapping for identification and quantification of iron deposition across the brain	10			x	[76]

https://doi.org/10.1371/journal.pone.0260952.t004

Table 5. Overview of cognitive, affective, and behavioral assessments at the campus.

Domain	Name of task	Measure	Description	Duration (minutes)	Assessment 1	Assessment 2	Assessment 3	Ref
**Cognition**	Foraging task	The tendency to explore alternatives vs. to exploit a chosen alternative	Participants are presented with a tree and have to decide whether to harvest it for apples and incur a short harvest delay or move to a new tree and incur a longer travel delay	30	x	x	x	[77]
**Cognition**	Serial random-dot motion discrimination task	How predictions from the past are weighted with uncertain sensory information in the present	Participants judge the motion direction of moving dots (up vs. down) and receive auditory feedback about the correctness of their response	25	x	x	x	[78]
**Cognition**	Reward-driven reach-adaptation task	How willing people are to search for more rewarding outcomes in a motor task	Participants make shooting movements toward a target while holding a handle that records pulling and hand rotation movements	20	x	x	x	[79]
**Cognition**	Paired associate memory task	Associative Memory	Participants memorize the associations between pictures of people and names in a study phase and the memory for these associations is tested in a test phase using a cued-recall-test	7	x	x	x	[80]
**Cognition**	Tower of London	Executive function (planning)	Participants are presented with a startling array of different colored, same-sized balls and are requested to move the balls one-by-one, with as little moves as possible to a predefined goal array.	5	x	x	x	[81]
**Affect**	Contextual fear generalization task	Fear generalization	Participants are instructed to attend to the presented stimuli and learn to predict the shock in multiple contexts while assessing eye-blink startle electromyography, subjective report, and avoidance tendencies.	40	x	x	x	[82]
**Affect**	Emotion regulation task	Emotion regulation	Participants are asked to actively regulate their emotions while either neutral or aversive pictures are presented on the computer screen	15	x	x	x	[83]
**Affect**	Self-referent encoding Task	Positive and negative memory bias	Participants endorse and memorize positive and negative words	8	x	x	x	[84]
**Affect**	Stimulus-response compatibility task	Automatic approach or avoidance tendency	Participants are presented with pictures (alcohol vs. soda) and are instructed to approach or avoid a certain condition	10	x	x	x	[85]
**Behavior**	Columbia card task	Risk preference	A card game that gives participants the repeated choice between risky options and safe options	22	x	x	x	[86]
**Behavior**	Food auction task	Reliable index of people's preference for hedonic (short-term reward) vs. healthy food (long-term reward)	Participants bid on different food items (e.g., package of M&Ms, apple)	15	x	x	x	[87]

https://doi.org/10.1371/journal.pone.0260952.t005

Table 6. Sensory assessments.

Domain	Measure	Duration (minutes)	Assessment 1	Assessment 2	Assessment 3
**Vision**	Contrast sensitivity	5	x		
	Visual acuity	5		x	
	Color vision	5			x
**Hearing**	Hearing ability	1	x	x	x

https://doi.org/10.1371/journal.pone.0260952.t006

Table 7. Post-visit online questionnaires.

Domain	Name of the questionnaire	What does it measure?	Duration (minutes)	Assessment 1	Assessment 2	Assessment 3	Ref
**Exposure**	Exposure	Exposure from environment	5	x	x	x	
**Health**	Over-the-counter medication	Use of nonprescription medication like pain relievers, cough suppressants, etc.	1	x	x	x	[88]
	Health complaints	Complaints like tiredness, nausea, back pain, headache, etc.	5	x	x	x	[89]
**Mental Health**	Adult ADHD Self-Report Scale (ASRS)	Symptom scale for ADHD	10	x			[90]
	Autistic Trait Questionnaire (ATQ)	Autistic traits	5	x			[91]
	Self-Report Inventory of Depressive Symptomatology (IDS-SR)	Presence and severity of depressive symptoms	5	x	x	x	[92]
	Anxiety Sensitivity Index (ASI)	Anxiety (trait)	5			x	[93]
	State and Trait Anxiety Inventory (STAI-S)	Anxiety (state)	5	x	x	x	[94]
	Perceived Stress Scale (PSS)	Stress	5	x	x	x	[95]
	Utrecht Burnout Scale (UBOS)	Burnout	3	x	x	x	[96]
	Reactive Proactive Aggression Questionnaire (RPQ)	Aggression	5	x	x	x	[97]
	Daily hassles	Daily hassles	5	x	x	x	[98]
	Cognitive emotion regulation questionnaire (CERQ)	Cognitive regulation of emotion	5	x	x	x	[99]
**Life events**	Childhood Trauma Questionnaire (CTQ)	Adverse childhood experiences	5		x		[100]
	Life events	Threatening life experiences	10	x	x	x	[101]
**Social/ Relationship**	UCLA loneliness scale	Loneliness	5	x	x	x	[102]
	Need to belong scale	Belongingness	3	x	x	x	[103]
	Multidimensional scale of Perceived Social Support (PSS)	Perceived social support	5	x	x	x	[104]
**Work**	Exposure to work	Working hours, working schedules, type of employment	4	x	x	x	
	Survey Work-home Interaction–NijmeGen (SWING)	Work-life balance	4	x	x	x	[105]
	Workplace commitment		5	x	x	x	[106]
	Employability		5	x	x	x	[107, 108]
	Questionnaire on the Experience and Evaluation of Work (QEEW)	Job characteristics	7	x*			[109]
**Politics**	Populism index	Attitude toward populism	2	x	x	x	
	Political efficacy	Attitude towards national government and politics	2	x	x	x	[110]
	Political participation	Political activities	1	x	x	x	
	EU membership	Attitude towards EU membership	1	x	x	x	[111]

(*Continued*)

Table 7. (Continued)

Domain	Name of the questionnaire	What does it measure?	Duration (minutes)	Assessment 1	Assessment 2	Assessment 3	Ref
**Personality**	BIG-5 NEO-FFI-3	Openness to experience, conscientiousness, neuroticism, extraversion, and agreeableness	10	x			[112]
	Sensory Processing Sensitivity (SPS)	High sensitivity	5			x	[113]
	Barratt Impulsiveness Scale (BIS-11)	Impulsiveness	10		x		[114]
	Self-control		10	x			[115]
	New general self-efficacy scale	Self-efficacy	5		x		[116]
	Dispositional greed	Greediness	3			x	[117]
	Dark triad	Narcissism, Machiavellianism, psychopathy	5	x			[118]
	Social investment attitudes	Attitudes toward corporate social responsibility	5		x		[119]
**Literacy**	Numeracy test	Mathematical abilities	12		x		[120]
	Financial literacy	Financial attitudes, skills	20			x	[121]
	Graph literacy	Ability to understand the meaning of graphs	10	x			[122]
	Cultural intelligence	Ability to relate and work effectively across cultures	2			x	[123]

*Participants fill out their job characteristics at the first assessment. In the second and third assessments, they fill out their job characteristics only in case of a new job.

https://doi.org/10.1371/journal.pone.0260952.t007

Examples of research opportunities

The HBS resource will be used to address expert and citizen-driven research questions that usually pertain to complex interactions between multiple factors. The first example of an expert-driven research question pertains to the association between income and positive affect. It was found among US residents that higher income was associated with more happiness and enjoyment, and less sadness and worry, but only up to a point ($75.000 per year), above that,

Table 8. Post-visit online assessments.

Domain	Online task	What does it measure?	Duration (minutes)	Assessment 1	Assessment 2	Assessment 3	Ref
**Decision-making**	Higher-order risk preferences	Risk attitudes, prudence, and temperance in financial decision-making	15	x	x	x	[124]
	Equality equivalence test	Social preferences	10	x	x	x	[125]
	Ambiguity	Ambiguous risk attitudes	10	x	x	x	[126]
	Trust game	Trust and trustworthiness	10	x	x	x	[127]
	Public good game	Altruism, conditional reciprocity	15	x	x	x	[128]
	Time preferences	Temporal discounting	8	x	x	x	[129]
**Language**	Narrative reading	Comprehension of and immersion into a narrative	15	x	x	x	[130]
**Solidarity**	Vignettes	Culpability, in/out group	15	x	x	x	[131, 132]

https://doi.org/10.1371/journal.pone.0260952.t008

Table 9. Individual differences in language skills test battery.

Domain	Online task	What does it measure?	Duration (minutes)	Ref
**Cognition**	Auditory simple and choice reaction time task	Processing speed	7	[133]
	Letter comparison	Processing speed	5	[134, 135]
	Visual simple and choice reaction time task	Processing speed	7	[133, 136]
	Digit span (forward & backward)	Auditory working memory	7	[137]
	Corsi block tapping (forward & backward)	Visual working memory	7	[138, 139]
	Raven's advanced progressive matrices	Non-verbal intelligence	25	[140]
**Linguistic knowledge**	Stairs4Words (2 Runs)	Linguistic experience: Vocabulary	7	
	Peabody Picture Vocabulary Test	Linguistic experience: Vocabulary	10	[141, 142]
	Idiom recognition test	Linguistic experience: Knowledge of idiomatic expressions	3	
	Spelling test	Linguistic experience: Spelling	5	
	Author recognition test	Linguistic experience: Print exposure	5	[143]
	Prescriptive grammar	Linguistic experience: Prescriptive grammar knowledge	10	[144]
**Linguistic processing**	Picture naming	Word production	7	[133]
	Rapid automatized naming	Word production	7	
	Verbal fluency	Word production	5	[145]
	Antonym production	Word production	5	[146]
	Maximal speech rate	Word production	3	
	Phrase generation	Sentence production	10	
	Sentence generation (active/passive sentence formulation)	Sentence production	12	
	Sentence generation (event apprehension)	Sentence production	10	
	Spontaneous speech	Sentence production	4	[147]
	Non-Word monitoring in non-word lists in noise	Word comprehension	10	
	Rhyme judgment	Word comprehension	5	
	Lexical decision	Word comprehension	7	[133]
	Semantic categorization	Word comprehension	5	
	Word monitoring in sentences in noise	Sentence comprehension	10	
	Grammatical gender cues	Sentence comprehension	10	[148]
	Verb-specific selective restrictions	Sentence comprehension	7	[149, 150]
	Self-paced reading	Sentence comprehension	5	

https://doi.org/10.1371/journal.pone.0260952.t009

there was no relationship between income and emotional well-being [151]. The HBS resource can help explain the interplay between affect, social and biological data, and income. A second example of a complex interaction is that sedentary behavior is associated with poor health and higher mortality [152, 153]. Merely standing up from time to time, e.g., to walk around a bit protects against part of this health risk [154]. Existing research on this topic has mainly focused on the consequences of prolonged sitting and has overlooked the key question of why people choose to stand up (when they sit) or sit down (when they stand), in the first place. In other words, what psychological processes (e.g., related to effort, reward, affect, and fatigue) are associated with healthy and unhealthy sedentary behavior? Answering this question will pave the

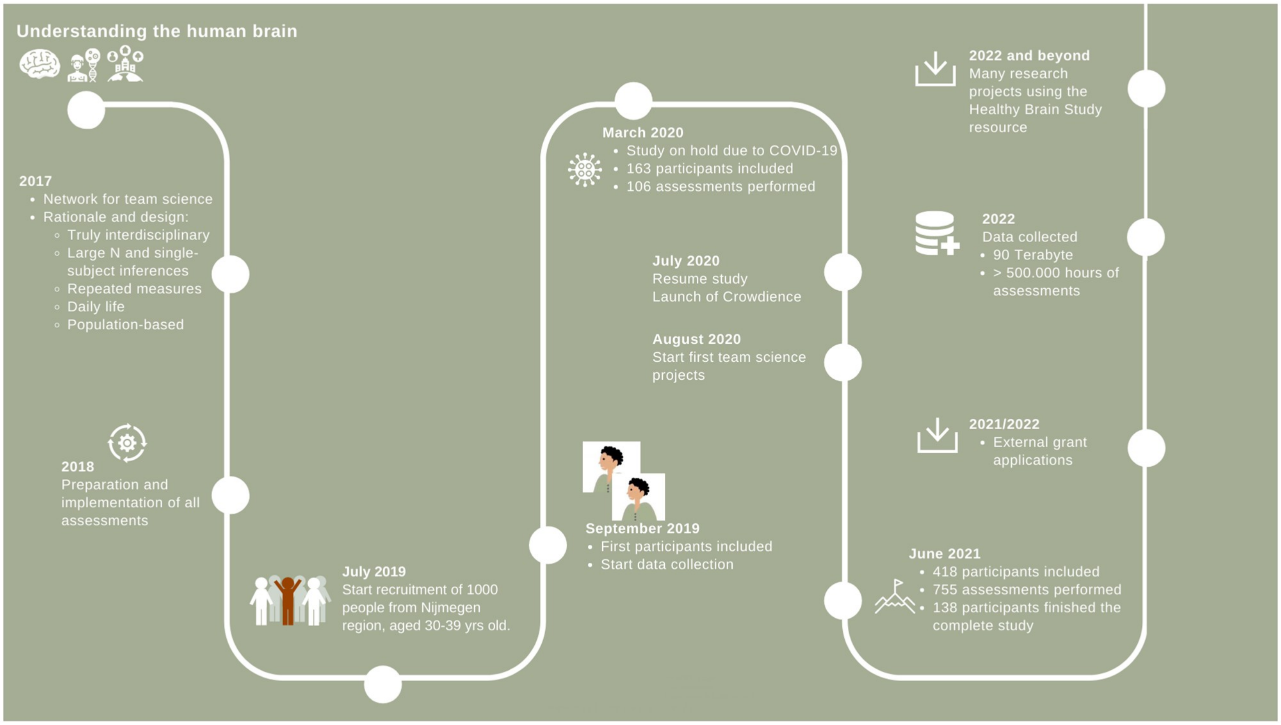

Fig 3. Progress and milestones of the healthy brain study.

https://doi.org/10.1371/journal.pone.0260952.g003

way for the development of novel, targeted interventions that will improve (occupational) health [155].

The HBS resource will also be used for citizen science. Different forms of citizen science exist. Projects can be led by experts, community-led, or co-created with different aims and levels of participation [156]. HBS participants and other citizens generate research questions to be answered with the HBS resource. In traditional designs, scientists test hypotheses that are often based on previous findings within their research domain or their intuitions. However, people living in or with specific conditions (i.e., being in their thirties and going through a key life event) may have additional insight on top of existing expert-knowledge. These insights are uncovered by a citizen science platform. The essence of the platform is to leverage collective intelligence from a large group of participants versus a smaller number of experts. This can reveal topics and research questions that have a significant influence on people's behavior in the real world and their health status, which experts may have left untouched [157–160]. By giving citizens a voice in scientific research, it can contribute considerably to the valorization of research results.

Reflection on design choices and study population

Comprehension of complex interactions as illustrated above requires an interdisciplinary, team science approach [29]. The HBS design is the result of an orchestrated cross-campus process over 22 months in which 250 scientists from all classical faculties were involved and were challenged to look past the horizons of their disciplines in a few plenary meetings and several smaller working groups, all providing input to a multidisciplinary scientific board that made the final design decisions. Here, we reflect on our design choices and the study population selected.

First of all, to capture the complexity of the human brain and its environment, a large set of measures was provided. We sought a balance between comprehensiveness, local expertise, costs, and burden for the participants. This resulted in an extensive number, variety, duration of mostly validated assessments, albeit not perfectly comprehensive. For example, the neuroimaging protocol is largely aligned with the Human Connectome Project [161] and UK Biobank [162] brain imaging, but includes a movie fMRI scan that is not included in the Human Connectome Project and UK Biobank, while the latter include scans that are not included in the HBS. Furthermore, instead of continuous monitoring over one year with validated wearable devices, the HBS covers three times a burst week of real-world assessments. Also, the collection of GPS data, financial transactions, and social media interactions were not allowed due to legal restrictions and privacy concerns.

Secondly, the HBS includes three repeated assessments for about one year. These repetitions aim to capture changes in human brain operations that may be related to relevant life events, seasonality, and/or incidental or dynamic changes in the biological and social environment. Regarding seasonality, the HBS participants start at varying time points within a year, so, although we sample only three instead of four times over one year, across participants we sample seasonal transitions in a fine-grained manner.

Thirdly, the HBS aims to include 1,000 participants. Due to differences in measurement-specific signal-to-noise properties, it is not possible to provide a straightforward power and sample size calculation because the data enables analyses of various cognitive, affective, and behavioral interactions in their bio-social context. To decide on the number of participants, we sought a balance between sensitivity and feasibility. The chosen number of participants is high compared to traditional neuroscientific experiments revealing general principles but is low compared to disease risk-oriented cohorts (which is not the aim of the HBS) like the UK Biobank [26], the Rotterdam Study [24], or the Rhineland Study [25]. However, the number is comparable to other studies designed to capture inter-individual differences like the Human Connectome Project, which included 1,200 young healthy adults [161], or the Personalized Parkinson Project, which included 650 patients [163]. We consider the number large given the comprehensive range of repeated measures both in the laboratory as in the real world.

Fourthly, we believe that assessing real-world events with wearable devices is more objective than scales and questionnaires. When we designed the study in 2017, hardly any longitudinal study included wearable devices. As the field of wearable technology has developed rapidly, in the meantime, several longitudinal studies have added wearable devices to their data collection methods. For example, subsets of UK Biobank participants and Rotterdam Study participants wore an accelerometer [164, 165]. We would like to stress that including real-world assessments is one of the five strengths of the HBS, so it is not unique by itself. In particular, the HBS includes repeated assessments with wearable devices in three different seasons over one year starting at varying time points within a year. When we designed the study, to our knowledge, this was unique for HBS. In the meantime, a subset of UK Biobank participants is performing seasonal repeats with a wearable device [166]. Furthermore, the HBS combines physiological recordings with wearable devices with ecological momentary assessments using a smartphone application. We consider the additional collection of momentary assessments of mood and behavior and context information innovative.

Fifthly, we developed a recruitment strategy targeted at a sample that represents the 30-39-year-old population of Nijmegen and its surroundings in terms of gender and educational attainment. However, a reasonable level of reading, speaking, and understanding Dutch (B1 level) is required to be able to complete the study protocol, e.g., to fill out questionnaires. This implies that the HBS participants do not fully represent the Nijmegen population at large, because in this example the illiterate, people with low literacy, or non-Dutch speaking

individuals are excluded. However, the aim of including 220 participants with a low, 340 with a middle, and 430 with a high level of education enables the study of interacting social factors.

Digital security system and data sharing

The HBS resource will be accessible to the scientific community at large. The resource contains sensitive personal data that needs to be protected from unauthorized access and unintentional disclosure. The sharing of (big) data within the scientific community is necessary for progress and maximizes scientific benefits derived from valuable and costly data. The HBS data is protected by a digital security system, a Polymorphic Encryption and Pseudonymization (PEP) infrastructure [36], which allows the sharing of data with researchers worldwide while safeguarding participants' privacy in line with the European General Data Protection Regulation. The digital security system is based on a multi-point, privacy-by-design strategy: (a) participants provide informed consent, also for the important element of data sharing; (b) signed contractual agreements with researchers are in place to ensure that no attempts towards de-pseudonymization, linking or commercialization of the raw data will be attempted; (c) governance policies limit access to the data to qualified researchers only; (d) an innovative pseudonymization and encryption process is applied [37].

An access procedure is in place and published on https://www.healthybrainstudy.nl/en/data-and-methods/access. We stratify researchers into three tiers with different rights. Tier I consists of researchers from the Radboud campus that contributed to study design or data acquisition. Tier II consists of all other researchers from the Radboud campus. Tier III consists of publicly financed researchers from other academic institutions. Companies can apply in all tiers, but they cannot apply independently. Application for data starts with the submission of a data request for a project that has been preregistered, e.g., in the Open Science Framework. Then, the HBS scientific board reviews the application. After approval, the researcher signs a data/material transfer agreement. Next, the researcher receives data and/or samples. The Radboud Biobank provides the samples [35]. All processed data and samples with relevant documentation (including scripts and data and/or samples processing protocols) must be integrated back into the HBS resource so that it can be used by others. Finally, the researcher publishes the results by acknowledging the HBS consortium.

Conclusion

The HBS has been designed using a team science approach to integrate scientific disciplines and is characterized by a broad range of repeated assessments, a large number of participants, both laboratory and real-world assessments, and a population-based sample. Moreover, data is managed to allow data sharing with scientists worldwide while maintaining participants' privacy. With the HBS resource, the scientific community can take the next step in understanding the human brain and how it dynamically and individually operates in its bio-social context.

Supporting information

S1 File. Detailed descriptions of measures included in healthy brain study.
(DOCX)

Acknowledgments

Preprint DOI: 10.31219/osf.io/fqmdu.

Author Contributions

Conceptualization: Esther Aarts, Agnes Akkerman, Mareike Altgassen, Ronald Bartels, Debby Beckers, Kirsten Bevelander, Erik Bijleveld, Esmeralda Blaney Davidson, Annemarie Boleij, Janita Bralten, Toon Cillessen, Jurgen Claassen, Roshan Cools, Martin Dresler, Thijs Eijsvogels, Myrthe Faber, Guillén Fernández, Bernd Figner, Matthias Fritsche, Sascha Füllbrunn, Surya Gayet, Marleen M. H. J. van Gelder, Marcel van Gerven, Sabine Geurts, Corina U. Greven, Martine Groefsema, Koen Haak, Peter Hagoort, Yvonne Hartman, Beatrice van der Heijden, Erno Hermans, Florian Hintz, Anneloes M. Hulsman, Martin Jaeger, Esther Janse, Joost Janzing, Roy P. C. Kessels, Johan C. Karremans, Willemien de Kleijn, Marieke Klein, Floris Klumpers, Nils Kohn, Hubert Korzilius, Floris de Lange, Judith van Leeuwen, Huaiyu Liu, Maartje Luijten, Peggy Manders, Katerina Manevska, José P. Marques, James M. McQueen, Pieter Medendorp, René Melis, Antje Meyer, Joukje Oosterman, Lucy Overbeek, Marius Peelen, Geert Postma, Karin Roelofs, Yvonne G. T. van Rossenberg, Gabi Schaap, Paul Scheepers, Luc Selen, Marianne Starren, Dorine W. Swinkels, Indira Tendolkar, Dick Thijssen, Hans Timmerman, Rayyan Tutunji, Anil Tuladhar, Harm Veling, Maaike Verhagen, Jacqueline Vink, Janna Vrijsen, Jana Vyrastekova, Selina van der Wal, Roel Willems, Arthur Willemsen.

Data curation: Willemien de Kleijn, Jasper Verkroost.

Funding acquisition: Guillén Fernández, Peter Hagoort, Arthur Willemsen.

Investigation: Ineke Cornelissen, Vivian Heuvelmans, Jon Matthews.

Methodology: Esther Aarts, Agnes Akkerman, Mareike Altgassen, Debby Beckers, Kirsten Bevelander, Erik Bijleveld, Esmeralda Blaney Davidson, Annemarie Boleij, Janita Bralten, Toon Cillessen, Jurgen Claassen, Roshan Cools, Ineke Cornelissen, Martin Dresler, Thijs Eijsvogels, Myrthe Faber, Bernd Figner, Matthias Fritsche, Sascha Füllbrunn, Surya Gayet, Marleen M. H. J. van Gelder, Marcel van Gerven, Sabine Geurts, Corina U. Greven, Martine Groefsema, Koen Haak, Yvonne Hartman, Beatrice van der Heijden, Erno Hermans, Florian Hintz, Anneloes M. Hulsman, Sebastian Idesis, Martin Jaeger, Esther Janse, Joost Janzing, Roy P. C. Kessels, Johan C. Karremans, Willemien de Kleijn, Marieke Klein, Floris Klumpers, Nils Kohn, Hubert Korzilius, Bas Krahmer, Floris de Lange, Judith van Leeuwen, Huaiyu Liu, Maartje Luijten, Peggy Manders, Katerina Manevska, José P. Marques, James M. McQueen, Pieter Medendorp, René Melis, Antje Meyer, Joukje Oosterman, Lucy Overbeek, Marius Peelen, Geert Postma, Karin Roelofs, Yvonne G. T. van Rossenberg, Gabi Schaap, Paul Scheepers, Luc Selen, Marianne Starren, Dorine W. Swinkels, Indira Tendolkar, Dick Thijssen, Hans Timmerman, Rayyan Tutunji, Anil Tuladhar, Harm Veling, Maaike Verhagen, Jacqueline Vink, Vivian Vriezekolk, Janna Vrijsen, Jana Vyrastekova, Selina van der Wal, Roel Willems.

Project administration: Guillén Fernández, Janet den Hollander, Lucy Overbeek.

Resources: Janet den Hollander, Peggy Manders, Jean Popma, Dorine W. Swinkels.

Supervision: Ineke Cornelissen, Guillén Fernández, Lucy Overbeek.

Writing – original draft: Lucy Overbeek.

Writing – review & editing: Esther Aarts, Agnes Akkerman, Mareike Altgassen, Ronald Bartels, Debby Beckers, Kirsten Bevelander, Erik Bijleveld, Esmeralda Blaney Davidson, Annemarie Boleij, Janita Bralten, Toon Cillessen, Jurgen Claassen, Roshan Cools, Ineke Cornelissen, Martin Dresler, Thijs Eijsvogels, Myrthe Faber, Guillén Fernández, Bernd Figner, Matthias Fritsche, Sascha Füllbrunn, Surya Gayet, Marleen M. H. J. van Gelder, Marcel

van Gerven, Sabine Geurts, Corina U. Greven, Martine Groefsema, Koen Haak, Peter Hagoort, Yvonne Hartman, Beatrice van der Heijden, Erno Hermans, Vivian Heuvelmans, Florian Hintz, Janet den Hollander, Anneloes M. Hulsman, Sebastian Idesis, Martin Jaeger, Esther Janse, Joost Janzing, Roy P. C. Kessels, Johan C. Karremans, Willemien de Kleijn, Marieke Klein, Floris Klumpers, Nils Kohn, Hubert Korzilius, Bas Krahmer, Floris de Lange, Judith van Leeuwen, Huaiyu Liu, Maartje Luijten, Peggy Manders, Katerina Manevska, José P. Marques, Jon Matthews, James M. McQueen, Pieter Medendorp, René Melis, Antje Meyer, Joukje Oosterman, Lucy Overbeek, Marius Peelen, Jean Popma, Geert Postma, Karin Roelofs, Yvonne G. T. van Rossenberg, Gabi Schaap, Paul Scheepers, Luc Selen, Marianne Starren, Dorine W. Swinkels, Indira Tendolkar, Dick Thijssen, Hans Timmerman, Rayyan Tutunji, Anil Tuladhar, Harm Veling, Maaike Verhagen, Jasper Verkroost, Jacqueline Vink, Vivian Vriezekolk, Janna Vrijsen, Jana Vyrastekova, Selina van der Wal, Roel Willems, Arthur Willemsen.

References

1. G-Science Academies Statement 2016. Understanding, protecting, and developing global brain resources. https://royalsociety.org/about-us/international/international-work/g-science-academies-meetings/.
2. Bruineberg J, Kiverstein J, Rietveld E. The anticipating brain is not a scientist: the free-energy principle from an ecological-enactive perspective. Synthese. 2018; 195(6):2417–44. https://doi.org/10.1007/s11229-016-1239-1 PMID: 30996493
3. Kiverstein J, Miller M. The embodied brain: towards a radical embodied cognitive neuroscience. Frontiers in Human Neuroscience. 2015; 9(237). https://doi.org/10.3389/fnhum.2015.00237 PMID: 25999836
4. Clark A. Being there: Putting brain, body, and world together again: MIT Press; 1996.
5. Chiel HJ, Beer RD. The brain has a body: adaptive behavior emerges from interactions of nervous system, body and environment. Trends Neurosci. 1997; 20(12):553–7. https://doi.org/10.1016/s0166-2236(97)01149-1 PMID: 9416664
6. Hermans EJ, van Marle HJ, Ossewaarde L, Henckens MJ, Qin S, van Kesteren MT, et al. Stress-related noradrenergic activity prompts large-scale neural network reconfiguration. Science. 2011; 334 (6059):1151–3. https://doi.org/10.1126/science.1209603 PMID: 22116887
7. Haeger A, Costa AS, Schulz JB, Reetz K. Cerebral changes improved by physical activity during cognitive decline: A systematic review on MRI studies. NeuroImage: Clinical. 2019; 23:101933. https://doi.org/10.1016/j.nicl.2019.101933 PMID: 31491837
8. Lyall LM, Wyse CA, Celis-Morales CA, Lyall DM, Cullen B, Mackay D, et al. Seasonality of depressive symptoms in women but not in men: A cross-sectional study in the UK Biobank cohort. J Affect Disord. 2018; 229:296–305. https://doi.org/10.1016/j.jad.2017.12.106 PMID: 29329063
9. Winthorst WH, Roest AM, Bos EH, Meesters Y, Penninx BW, Nolen WA, et al. Self-attributed seasonality of mood and behavior: a report from the Netherlands study of depression and anxiety. Depress Anxiety. 2014; 31(6):517–23. https://doi.org/10.1002/da.22130 PMID: 23695951
10. Cepeda M, Koolhaas CM, van Rooij FJA, Tiemeier H, Guxens M, Franco OH, et al. Seasonality of physical activity, sedentary behavior, and sleep in a middle-aged and elderly population: The Rotterdam study. Maturitas. 2018; 110:41–50. https://doi.org/10.1016/j.maturitas.2018.01.016 PMID: 29563034
11. Miller R, Stalder T, Jarczok M, Almeida DM, Badrick E, Bartels M, et al. The CIRCORT database: Reference ranges and seasonal changes in diurnal salivary cortisol derived from a meta-dataset comprised of 15 field studies. Psychoneuroendocrinology. 2016; 73:16–23. https://doi.org/10.1016/j.psyneuen.2016.07.201 PMID: 27448524
12. Davenport ER, Mizrahi-Man O, Michelini K, Barreiro LB, Ober C, Gilad Y. Seasonal variation in human gut microbiome composition. PLoS One. 2014; 9(3):e90731. https://doi.org/10.1371/journal.pone.0090731 PMID: 24618913
13. Ockene IS, Chiriboga DE, Stanek EJ 3rd, Harmatz MG, Nicolosi R, Saperia G, et al. Seasonal variation in serum cholesterol levels: treatment implications and possible mechanisms. Arch Intern Med. 2004; 164(8):863–70. https://doi.org/10.1001/archinte.164.8.863 PMID: 15111372

14. Marti-Soler H, Gubelmann C, Aeschbacher S, Alves L, Bobak M, Bongard V, et al. Seasonality of cardiovascular risk factors: an analysis including over 230 000 participants in 15 countries. Heart. 2014; 100(19):1517–23. https://doi.org/10.1136/heartjnl-2014-305623 PMID: 24879630

15. Llera A, Wolfers T, Mulders P, Beckmann CF. Inter-individual differences in human brain structure and morphology link to variation in demographics and behavior. eLife. 2019; 8:e44443. https://doi.org/10.7554/eLife.44443 PMID: 31268418

16. Smith SM, Nichols TE, Vidaurre D, Winkler AM, Behrens TE, Glasser MF, et al. A positive-negative mode of population covariation links brain connectivity, demographics and behavior. Nat Neurosci. 2015; 18(11):1565–7. https://doi.org/10.1038/nn.4125 PMID: 26414616

17. Myin-Germeys I, Kasanova Z, Vaessen T, Vachon H, Kirtley O, Viechtbauer W, et al. Experience sampling methodology in mental health research: new insights and technical developments. World Psychiatry. 2018; 17(2):123–32. https://doi.org/10.1002/wps.20513 PMID: 29856567

18. Hogenelst K, Schoevers RA, aan het Rot M. Studying the neurobiology of human social interaction: Making the case for ecological validity. Social Neuroscience. 2015; 10(3):219–29. https://doi.org/10.1080/17470919.2014.994786 PMID: 25566795

19. Fry A, Littlejohns TJ, Sudlow C, Doherty N, Adamska L, Sprosen T, et al. Comparison of sociodemographic and health-related characteristics of UK Biobank participants with those of the general population. Am J Epidemiol. 2017; 186(9):1026–34. https://doi.org/10.1093/aje/kwx246 PMID: 28641372

20. Henrich J, Heine SJ, Norenzayan A. The weirdest people in the world? The Behavioral and brain sciences. 2010; 33(2–3):61–83; discussion -135. https://doi.org/10.1017/S0140525X0999152X PMID: 20550733

21. Jaddoe VWV, Mackenbach JP, Moll HA, Steegers EAP, Tiemeier H, Verhulst FC, et al. The Generation R Study: Design and cohort profile. European Journal of Epidemiology. 2006; 21(6):475. https://doi.org/10.1007/s10654-006-9022-0 PMID: 16826450

22. Volkow ND, Koob GF, Croyle RT, Bianchi DW, Gordon JA, Koroshetz WJ, et al. The conception of the ABCD study: From substance use to a broad NIH collaboration. Developmental Cognitive Neuroscience. 2018; 32:4–7. https://doi.org/10.1016/j.dcn.2017.10.002 PMID: 29051027

23. Niermann HCM, Tyborowska A, Cillessen AHN, van Donkelaar MM, Lammertink F, Gunnar MR, et al. The relation between infant freezing and the development of internalizing symptoms in adolescence: A prospective longitudinal study. Dev Sci. 2019; 22(3):e12763. https://doi.org/10.1111/desc.12763 PMID: 30318656

24. Hofman A, Breteler MM, van Duijn CM, Krestin GP, Pols HA, Stricker BH, et al. The Rotterdam Study: objectives and design update. Eur J Epidemiol. 2007; 22(11):819–29. https://doi.org/10.1007/s10654-007-9199-x PMID: 17955331

25. Breteler M, Wolf H. The Rhineland study: a novel platform for epidemiologic research into Alzeimer disease and related disorders. Alzheimer's & Dementia. 2014; 10.

26. Sudlow C, Gallacher J, Allen N, Beral V, Burton P, Danesh J, et al. UK biobank: an open access resource for identifying the causes of a wide range of complex diseases of middle and old age. PLoS Med. 2015; 12(3):e1001779. https://doi.org/10.1371/journal.pmed.1001779 PMID: 25826379

27. Nyberg L, Boraxbekk C-J, Sörman DE, Hansson P, Herlitz A, Kauppi K, et al. Biological and environmental predictors of heterogeneity in neurocognitive ageing: Evidence from Betula and other longitudinal studies. Ageing Research Reviews. 2020; 64:101184. https://doi.org/10.1016/j.arr.2020.101184 PMID: 32992046

28. Sowell ER, Peterson BS, Thompson PM, Welcome SE, Henkenius AL, Toga AW. Mapping cortical change across the human life span. Nat Neurosci. 2003; 6(3):309–15. https://doi.org/10.1038/nn1008 PMID: 12548289

29. Utzerath C, Fernández G. Shaping science for increasing interdependence and specialization. Trends in Neurosciences. 2017; 40(3):121–4. https://doi.org/10.1016/j.tins.2016.12.005 PMID: 28126248

30. Statistics Netherlands: https://www.cbs.nl

31. Association WM. World Medical Association Declaration of Helsinki: ethical principles for medical research involving human subjects. JAMA. 2013; 310(20):2191–4. https://doi.org/10.1001/jama.2013.281053 PMID: 24141714

32. https://stichtingcrowdience.nl/.

33. https://www.health-ri.nl/services/ldot-workflow-tool-clinical-research.

34. https://data.castoredc.com.

35. Manders P, Lutomski J, Smit C, Swinkels D, Zielhuis G. The Radboud Biobank: A central facility for disease-based biobanks to optimise use and distribution of biomaterial for scientific research in the Radboud University Medical Center, Nijmegen. Open Journal of Bioresources. 2018; 5.

36. Verheul ER, Jacobs B, Meijer C, Hildebrandt M, de Ruiter J. Polymorphic encryption and pseudonymisation for personalised healthcare. IACR Cryptology ePrint Archive. 2016: 411.

37. Jacobs B, Popma J. Medical research, big data and the need for privacy by design. Big Data & Society. 2019; 6(1):2053951718824352.

38. https://www.lissdata.nl/.

39. Vink JM, Willemsen G, Beem AL, Boomsma DI. The Fagerstrom test for nicotine dependence in a Dutch sample of daily smokers and ex-smokers. Addict Behav. 2005; 30(3):575–9. https://doi.org/10.1016/j.addbeh.2004.05.023 PMID: 15718074

40. Saunders JB, Aasland OG, Babor TF, de la Fuente JR, Grant M. Development of the Alcohol Use Disorders Identification Test (AUDIT): WHO collaborative project on early detection of persons with harmful alcohol consumption. Addiction. 1993; 88(6):791–804. https://doi.org/10.1111/j.1360-0443.1993.tb02093.x PMID: 8329970

41. Schippers GM, Broekman TG. MATE-Q 2.1. Handleiding. Nijmegen: Bêta Boeken. 2014.

42. Feunekes GI, Van Staveren WA, De Vries JH, Burema J, Hautvast JG. Relative and biomarker-based validity of a food-frequency questionnaire estimating intake of fats and cholesterol. Am J Clin Nutr. 1993; 58(4):489–96. https://doi.org/10.1093/ajcn/58.4.489 PMID: 8379504

43. Molag ML, de Vries JH, Duif N, Ocke MC, Dagnelie PC, Goldbohm RA, et al. Selecting informative food items for compiling food-frequency questionnaires: comparison of procedures. Br J Nutr. 2010; 104(3):446–56. https://doi.org/10.1017/S0007114510000401 PMID: 20374682

44. Siebelink E, Geelen A, de Vries JH. Self-reported energy intake by FFQ compared with actual energy intake to maintain body weight in 516 adults. Br J Nutr. 2011; 106(2):274–81. https://doi.org/10.1017/S0007114511000067 PMID: 21338536

45. Verkleij-Hagoort AC, de Vries JH, Stegers MP, Lindemans J, Ursem NT, Steegers-Theunissen RP. Validation of the assessment of folate and vitamin B12 intake in women of reproductive age: the method of triads. Eur J Clin Nutr. 2007; 61(5):610–5. https://doi.org/10.1038/sj.ejcn.1602581 PMID: 17164826

46. Rosenberg DE, Norman GJ, Wagner N, Patrick K, Calfas KJ, Sallis JF. Reliability and validity of the Sedentary Behavior Questionnaire (SBQ) for adults. J Phys Act Health. 2010; 7(6):697–705. https://doi.org/10.1123/jpah.7.6.697 PMID: 21088299

47. Buysse DJ, Reynolds CF, Monk TH, Berman SR, Kupfer DJ. The Pittsburgh Sleep Quality Index: a new instrument for psychiatric practice and research. Psychiatry Res. 1989; 28(2):193–213. https://doi.org/10.1016/0165-1781(89)90047-4 PMID: 2748771

48. Schredl M. Reliability and stability of a dream recall frequency scale. Percept Mot Skills. 2004; 98(3 Pt 2):1422–6. https://doi.org/10.2466/pms.98.3c.1422-1426 PMID: 15291233

49. Lemmens JS, Valkenburg PM, Gentile DA. The internet gaming disorder scale. Psychol Assess. 2015; 27(2):567–82. https://doi.org/10.1037/pas0000062 PMID: 25558970

50. van den Eijnden RJJM, Lemmens JS, Valkenburg PM. The social media disorder scale. Comput Hum Behav. 2016; 61:478–87.

51. Baumgartner SE, Lemmens JS, Weeda WD, Huizinga M. Measuring media multitasking: development of a short measure of media multitasking for adolescents. Journal of Media Psychology: Theories, Methods, and Applications. 2017; 92(2):92–101.

52. Diener E, Emmons RA, Larsen RJ, Griffin S. The satisfaction with life scale. J Pers Assess. 1985; 49(1):71–5. https://doi.org/10.1207/s15327752jpa4901_13 PMID: 16367493

53. Bjørnskov C. How comparable are the Gallup world poll life satisfaction data? Journal of Happiness Studies. 2010; 11(1):41–60.

54. Bohlmeijer E, ten Klooster PM, Fledderus M, Veehof M, Baer R. Psychometric properties of the five facet mindfulness questionnaire in depressed adults and development of a short form. Assessment. 2011; 18(3):308–20. https://doi.org/10.1177/1073191111408231 PMID: 21586480

55. Åstrand PO, Ryhming I. A nomogram for calculation of aerobic capacity (physical fitness) from pulse rate during sub-maximal work. J Appl Physiol. 1954; 7(2):218–21. https://doi.org/10.1152/jappl.1954.7.2.218 PMID: 13211501

56. Kozey-Keadle S, Libertine A, Lyden K, Staudenmayer J, Freedson PS. Validation of wearable monitors for assessing sedentary behavior. Med Sci Sports Exerc. 2011; 43(8):1561–7. https://doi.org/10.1249/MSS.0b013e31820ce174 PMID: 21233777

57. Ryan CG, Grant PM, Tigbe WW, Granat MH. The validity and reliability of a novel activity monitor as a measure of walking. British journal of sports medicine. 2006; 40(9):779–84. https://doi.org/10.1136/bjsm.2006.027276 PMID: 16825270

58. Garbarino M, Lai M, Bender D, Picard R, Tognetti S. Empatica E3—A wearable wireless multi-sensor device for real-time computerized biofeedback and data acquisition. 2015:39–42.

59. Grillon C, Ameli R, Woods SW, Merikangas K, Davis M. Fear-potentiated startle in humans: effects of anticipatory anxiety on the acoustic blink reflex. Psychophysiology. 1991; 28(5):588–95. https://doi.org/10.1111/j.1469-8986.1991.tb01999.x PMID: 1758934

60. Aitken RC. Measurement of feelings using visual analogue scales. Proc R Soc Med. 1969; 62 (10):989–93. PMID: 4899510

61. Shanholtzer BA, Patterson SM. Use of bioelectrical impedance in hydration status assessment: reliability of a new tool in psychophysiology research. Int J Psychophysiol. 2003; 49(3):217–26. https://doi.org/10.1016/s0167-8760(03)00143-0 PMID: 14507440

62. Collins SL, Moore RA, McQuay HJ. The visual analogue pain intensity scale: what is moderate pain in millimetres? Pain. 1997; 72(1–2):95–7. https://doi.org/10.1016/s0304-3959(97)00005-5 PMID: 9272792

63. Timmerman H, Wilder-Smith O, van Weel C, Wolff A, Vissers K. Detecting the neuropathic pain component in the clinical setting: a study protocol for validation of screening instruments for the presence of a neuropathic pain component. BMC Neurol. 2014; 14:94. https://doi.org/10.1186/1471-2377-14-94 PMID: 24885108

64. Timmerman H, Wilder-Smith OH, Steegers MA, Vissers KC, Wolff AP. The added value of bedside examination and screening QST to improve neuropathic pain identification in patients with chronic pain. J Pain Res. 2018; 11:1307–18. https://doi.org/10.2147/JPR.S154698 PMID: 30022849

65. Lanier GM, Orlanes K, Hayashi Y, Murphy J, Flannery M, Te-Frey R, et al. Validity and reliability of a novel slow cuff-deflation system for noninvasive blood pressure monitoring in patients with continuous-flow left ventricular assist device. Circ Heart Fail. 2013; 6(5):1005–12. https://doi.org/10.1161/CIRCHEARTFAILURE.112.000186 PMID: 23811966

66. van Mil AC, Hartman Y, van Oorschot F, Heemels A, Bax N, Dawson EA, et al. Correlation of carotid artery reactivity with cardiovascular risk factors and coronary artery vasodilator responses in asymptomatic, healthy volunteers. J Hypertens. 2017; 35(5):1026–34. https://doi.org/10.1097/HJH.0000000000001274 PMID: 28129249

67. Faith JJ, Guruge JL, Charbonneau M, Subramanian S, Seedorf H, Goodman AL, et al. The long-term stability of the human gut microbiota. Science. 2013; 341(6141):1237439. https://doi.org/10.1126/science.1237439 PMID: 23828941

68. Cherbuin N, Kumar R, Sachdev PS, Anstey KJ. Dietary mineral intake and risk of mild cognitive impairment: the PATH through life project. Front Aging Neurosci. 2014; 6:4. https://doi.org/10.3389/fnagi.2014.00004 PMID: 24550825

69. Schwabe L, Haddad L, Schachinger H. HPA axis activation by a socially evaluated cold-pressor test. Psychoneuroendocrinology. 2008; 33(6):890–5. https://doi.org/10.1016/j.psyneuen.2008.03.001 PMID: 18403130

70. Wright KD, Hickman R, Laudenslager ML. Hair cortisol analysis: A promising biomarker of HPA activation in older adults. Gerontologist. 2015; 55 Suppl 1:S140–5. https://doi.org/10.1093/geront/gnu174 PMID: 26055775

71. Aerts R, Joly L, Szternfeld P, Tsilikas K, De Cremer K, Castelain P, et al. Silicone wristband passive samplers yield highly individualized pesticide residue exposure profiles. Environmental Science & Technology. 2018; 52(1):298–307. https://doi.org/10.1021/acs.est.7b05039 PMID: 29185731

72. O'Connell SG, Kincl LD, Anderson KA. Silicone wristbands as personal passive samplers. Environmental Science & Technology. 2014; 48(6):3327–35.

73. Glasser MF, Sotiropoulos SN, Wilson JA, Coalson TS, Fischl B, Andersson JL, et al. The minimal preprocessing pipelines for the Human Connectome Project. Neuroimage. 2013; 80:105–24. https://doi.org/10.1016/j.neuroimage.2013.04.127 PMID: 23668970

74. Diaz BA, Van Der Sluis S, Benjamins JS, Stoffers D, Hardstone R, Mansvelder HD, et al. The ARSQ 2.0 reveals age and personality effects on mind-wandering experiences. Front Psychol. 2014; 5:271. https://doi.org/10.3389/fpsyg.2014.00271 PMID: 24772097

75. Marques JP, Kober T, Krueger G, van der Zwaag W, Van de Moortele PF, Gruetter R. MP2RAGE, a self bias-field corrected sequence for improved segmentation and T1-mapping at high field. Neuroimage. 2010; 49(2):1271–81. https://doi.org/10.1016/j.neuroimage.2009.10.002 PMID: 19819338

76. Langkammer C, Schweser F, Krebs N, Deistung A, Goessler W, Scheurer E, et al. Quantitative susceptibility mapping (QSM) as a means to measure brain iron? A post mortem validation study. Neuroimage. 2012; 62(3):1593–9. https://doi.org/10.1016/j.neuroimage.2012.05.049 PMID: 22634862

77. Constantino SM, Daw ND. Learning the opportunity cost of time in a patch-foraging task. Cogn Affect Behav Neurosci. 2015; 15(4):837–53. https://doi.org/10.3758/s13415-015-0350-y PMID: 25917000

78. Braun A, Urai AE, Donner TH. Adaptive history biases result from confidence-weighted accumulation of past choices. J Neurosci. 2018. https://doi.org/10.1523/JNEUROSCI.2189-17.2017 PMID: 29371318

79. Therrien AS, Wolpert DM, Bastian AJ. Effective reinforcement learning following cerebellar damage requires a balance between exploration and motor noise. Brain. 2016; 139(Pt 1):101–14. https://doi.org/10.1093/brain/awv329 PMID: 26626368

80. Persson J, Kalpouzos G, Nilsson LG, Ryberg M, Nyberg L. Preserved hippocampus activation in normal aging as revealed by fMRI. Hippocampus. 2011; 21(7):753–66. https://doi.org/10.1002/hipo.20794 PMID: 20865729

81. Oosterman JM, Wijers M, Kessels RP. Planning or something else? Examining neuropsychological predictors of Zoo Map performance. Appl Neuropsychol Adult. 2013; 20(2):103–9. https://doi.org/10.1080/09084282.2012.670150 PMID: 23397996

82. Andreatta M, Glotzbach-Schoon E, Muhlberger A, Schulz SM, Wiemer J, Pauli P. Initial and sustained brain responses to contextual conditioned anxiety in humans. Cortex. 2015; 63:352–63. https://doi.org/10.1016/j.cortex.2014.09.014 PMID: 25460498

83. Webb TL, Miles E, Sheeran P. Dealing with feeling: a meta-analysis of the effectiveness of strategies derived from the process model of emotion regulation. Psychol Bull. 2012; 138(4):775–808. https://doi.org/10.1037/a0027600 PMID: 22582737

84. Derry PA, Kuiper NA. Schematic processing and self-reference in clinical depression. J Abnorm Psychol. 1981; 90(4):286–97. https://doi.org/10.1037//0021-843x.90.4.286 PMID: 7264058

85. Groefsema M, Engels R, Kuntsche E, Smit K, Luijten M. Cognitive biases for social alcohol-related pictures and alcohol use in specific social settings: an event-level study. Alcohol Clin Exp Res. 2016; 40 (9):2001–10. https://doi.org/10.1111/acer.13165 PMID: 27511292

86. Figner B, Mackinlay RJ, Wilkening F, Weber EU. Affective and deliberative processes in risky choice: age differences in risk taking in the Columbia Card Task. J Exp Psychol Learn Mem Cogn. 2009; 35 (3):709–30. https://doi.org/10.1037/a0014983 PMID: 19379045

87. Veling H, Chen Z, Tombrock MC, Verpaalen IAM, Schmitz LI, Dijksterhuis A, et al. Training impulsive choices for healthy and sustainable food. J Exp Psychol Appl. 2017; 23(2):204–15. https://doi.org/10.1037/xap0000112 PMID: 28150960

88. van Dijk L, van der Maat M, Salimans R, Bouvy M. De balans tussen verkrijgbaarheid en veiligheid. Evaluatie van de indeling van zelfzorggeneesmiddelen en de rol van drogist en apotheek bij de verstrekking. Nivel. 2010.

89. Yzermans J, Baliatsas C, van Dulmen S, Van Kamp I. Assessing non-specific symptoms in epidemiological studies: Development and validation of the Symptoms and Perceptions (SaP) questionnaire. International Journal of Hygiene and Environmental Health. 2016; 219(1):53–65. https://doi.org/10.1016/j.ijheh.2015.08.006 PMID: 26358929

90. Kessler RC, Adler L, Ames M, Demler O, Faraone S, Hiripi E, et al. The World Health Organization adult ADHD Self-Report Scale (ASRS): a short screening scale for use in the general population. Psychol Med. 2005; 35(2):245–56. https://doi.org/10.1017/s0033291704002892 PMID: 15841682

91. Bralten J, van Hulzen KJ, Martens MB, Galesloot TE, Arias Vasquez A, Kiemeney LA, et al. Autism spectrum disorders and autistic traits share genetics and biology. Mol Psychiatry. 2018; 23(5):1205–12. https://doi.org/10.1038/mp.2017.98 PMID: 28507316

92. Rush AJ, Gullion CM, Basco MR, Jarrett RB, Trivedi MH. The Inventory of Depressive Symptomatology (IDS): psychometric properties. Psychol Med. 1996; 26(3):477–86. https://doi.org/10.1017/s0033291700035558 PMID: 8733206

93. Rodriguez BF, Bruce SE, Pagano ME, Spencer MA, Keller MB. Factor structure and stability of the Anxiety Sensitivity Index in a longitudinal study of anxiety disorder patients. Behav Res Ther. 2004; 42 (1):79–91. https://doi.org/10.1016/s0005-7967(03)00074-3 PMID: 14744525

94. Spielberger CD, Gorsuch RL, Lushene R, Vagg PR, Jacobs GA. State-Trait Anxiety Inventory for adults -manual. 1983.

95. Cohen S, Kamarck T, Mermelstein R. A global measure of perceived stress. J Health Soc Behav. 1983; 24(4):385–96. PMID: 6668417

96. Schaufeli W, Dierendonck Dv. Utrechtse burn-out schaal: handleiding. Amsterdam: Pearson Assessment and Information BV 1981, 2001.

97. Cima M, Raine A, Meesters C, Popma A. Validation of the Dutch Reactive Proactive Questionnaire (RPQ): differential correlates of reactive and proactive aggression from childhood to adulthood. Aggress Behav. 2013; 39(2):99–113. https://doi.org/10.1002/ab.21458 PMID: 23386470

98. Kanner AD, Coyne JC, Schaefer C, Lazarus RS. Comparison of two modes of stress measurement: daily hassles and uplifts versus major life events. J Behav Med. 1981; 4(1):1–39. https://doi.org/10.1007/BF00844845 PMID: 7288876

99. Garnefski N, Kraaij V, Spinhoven P. CERQ. Handleiding voor het gebruik van de Cognitive Emotion Regulation Questionnaire. Leiderdorp: DATEC. 2002.

100. Bernstein DP, Stein JA, Newcomb MD, Walker E, Pogge D, Ahluvalia T, et al. Development and validation of a brief screening version of the Childhood Trauma Questionnaire. Child Abuse Negl. 2003; 27(2):169–90. https://doi.org/10.1016/s0145-2134(02)00541-0 PMID: 12615092

101. Brugha TS, Cragg D. The list of threatening experiences: the reliability and validity of a brief life events questionnaire. Acta Psychiatr Scand. 1990; 82(1):77–81. https://doi.org/10.1111/j.1600-0447.1990.tb01360.x PMID: 2399824

102. Russell D, Peplau LA, Cutrona CE. The revised UCLA Loneliness Scale: concurrent and discriminant validity evidence. J Pers Soc Psychol. 1980; 39(3):472–80. https://doi.org/10.1037//0022-3514.39.3.472 PMID: 7431205

103. Leary MR. Need to belong scale. Measurement instrument database for the social science. www.midss.ie 2013.

104. Zimet GD, Powell SS, Farley GK, Werkman S, Berkoff KA. Psychometric characteristics of the Multidimensional Scale of Perceived Social Support. J Pers Assess. 1990; 55(3–4):610–7. https://doi.org/10.1080/00223891.1990.9674095 PMID: 2280326

105. Geurts S, Taris T, Kompier M, Dikkers J, van Hooff M, Kinnunen U. Work-home interaction from a work psychological perspective: Development and validation of a new questionnaire, the SWING. Work & Stress. 2005; 19.

106. Klein HJ, Cooper JT, Molloy JC, Swanson JA. The assessment of commitment: Advantages of a unidimensional, target-free approach. Journal of Applied Psychology. 2014; 99(2):222–38. https://doi.org/10.1037/a0034751 PMID: 24188389

107. van der Heijden BIJM, Notelaers G, Peters P, Stoffers J, de Lange AH, Froehlich D, et al. Development and validation of the short-form employability five-factor instrument. Journal of Vocational Behavior. 2018; 106:236–48.

108. van der Heijden BI. Professional expertise of higher level employees; age stereotyping in self-assessments and supervisor ratings. Tijdschr Gerontol Geriatr. 2000; 31(2):62–9. PMID: 10816893

109. van Veldhoven MJPM, Prins J, van der Laken PA, Dijkstra L. BBA2.0: Update van de standaard voor vragenlijstonderzoek naar werk, welbevinden en prestaties. Amsterdam: SKB. 2014.

110. Craig SC. Efficacy, trust, and political behavior: an attempt to resolve a lingering conceptual dilemma. American Politics Quarterly. 1979; 7(2):225–39.

111. Kuhn T, van Elsas E, Hakhverdian A, van der Brug W. An ever wider gap in an ever closer union: Rising inequalities and euroscepticism in 12 West European democracies, 1975–2009. Socio-Economic Review. 2016; 14(1):27–45.

112. Costa P, R. McCrae R. The revised NEO personality inventory (NEO-PI-R)2008. 179–98 p.

113. Aron EN, Aron A, Jagiellowicz J. Sensory processing sensitivity: a review in the light of the evolution of biological responsivity. Pers Soc Psychol Rev. 2012; 16(3):262–82. https://doi.org/10.1177/1088868311434213 PMID: 22291044

114. Patton JH, Stanford MS, Barratt ES. Factor structure of the Barratt impulsiveness scale. J Clin Psychol. 1995; 51(6):768–74. https://doi.org/10.1002/1097-4679(199511)51:6<768::aid-jclp2270510607>3.0.co;2-1 PMID: 8778124

115. Tangney JP, Baumeister RF, Boone AL. High self-control predicts good adjustment, less pathology, better grades, and interpersonal success. J Pers. 2004; 72(2):271–324. https://doi.org/10.1111/j.0022-3506.2004.00263.x PMID: 15016066

116. Chen G, Gully SM, Eden D. Validation of a new general self-efficacy scale. Organizational Research Methods. 2001; 4(1):62–83.

117. Seuntjens TG, Zeelenberg M, van de Ven N, Breugelmans SM. Dispositional greed. J Pers Soc Psychol. 2015; 108(6):917–33. https://doi.org/10.1037/pspp0000031 PMID: 25664899

118. Jonason PK, Webster GD. The dirty dozen: a concise measure of the dark triad. Psychol Assess. 2010; 22(2):420–32. https://doi.org/10.1037/a0019265 PMID: 20528068

119. Williams G. Some determinants of the socially responsible investment decision: A cross-country study. Journal of Behavioral Finance. 2007; 8(1):43–57.

120. Weller JA, Dieckmann NF, Tusler M, Mertz CK, Burns WJ, Peters E. Development and testing of an abbreviated numeracy scale: A rasch analysis approach. Journal of Behavioral Decision Making. 2013; 26(2):198–212. https://doi.org/10.1002/bdm.1751 PMID: 32313367

121. Atkinson A, Messy F-A. Assessing financial literacy in 12 countries: an OECD/INFE international pilot exercise. Journal of Pension Economics and Finance. 2011; 10(04):657–65.

122. Galesic M, Garcia-Retamero R. Graph literacy: A cross-cultural comparison. Medical decision making. 2011; 31(3):444–57. https://doi.org/10.1177/0272989X10373805 PMID: 20671213

123. Thomas D, Liao Y, Aycan Z, Cerdin J-L, Pekerti A, Ravlin E, et al. Cultural intelligence: A theory-based, short form measure2015.

124. Noussair CN, Trautmann ST, van de Kuilen G. Higher order risk attitudes, demographics, and financial decisions. The Review of Economic Studies. 2014; 81(1):325–55.

125. Kerschbamer R. The geometry of distributional preferences and a non-parametric identification approach: The Equality Equivalence Test. European Economic Review. 2015; 76:85–103. https://doi.org/10.1016/j.euroecorev.2015.01.008 PMID: 26089571

126. Dimmock SG, Kouwenberg R, Wakker PP. Ambiguity attitudes in a large representative sample. Management Science. 2016; 62(5):1363–80.

127. Hergueux J, Jacquemet N. Social preferences in the online laboratory: a randomized experiment. Experimental Economics. 2015; 18(2):251–83.

128. Fischbacher U, Gächter S, Quercia S. The behavioral validity of the strategy method in public good experiments. Journal of Economic Psychology. 2012; 33(4):897–913.

129. Falk A, Becker A, Dohmen T, Enke B, Huffman D, Sunde U. Global evidence on economic preferences. The Quarterly Journal of Economics. 2018; 133(4):1645–92.

130. Kuijpers M, Hakemulder F, Tan E, Doicaru M. Exploring absorbing reading experiences: Developing and validating a self-report scale to measure story world absorption. Scientific Study of Literature. 2014; 4.

131. Jasso G, Opp KD. Probing the character of norms: A factorial survey analysis of the norms of political action. American Sociological Review. 1997; 62:947.

132. Rooks G, Raub W, Selten R, Tazelaar F. How inter-firm co-operation depends on social embeddedness: A vignette study. Acta Sociologica. 2000; 43(2):123–37.

133. Hintz F, Jongman SR, Dijkhuis M, van 't Hoff V, McQueen JM, Meyer AS. Shared lexical access processes in speaking and listening? An individual differences study. Journal of Experimental Psychology: Learning, Memory, and Cognition. 2020; 46(6):1048–63. https://doi.org/10.1037/xlm0000768 PMID: 31599623

134. Salthouse TA. The processing-speed theory of adult age differences in cognition. Psychol Rev. 1996; 103(3):403–28. https://doi.org/10.1037/0033-295x.103.3.403 PMID: 8759042

135. Earles JL, Salthouse TA. Interrelations of age, health, and speed. J Gerontol B Psychol Sci Soc Sci. 1995; 50(1):P33–p41. https://doi.org/10.1093/geronb/50b.1.p33 PMID: 7757821

136. Deary IJ, Liewald D, Nissan J. A free, easy-to-use, computer-based simple and four-choice reaction time programme: the Deary-Liewald reaction time task. Behav Res Methods. 2011; 43(1):258–68. https://doi.org/10.3758/s13428-010-0024-1 PMID: 21287123

137. Wechsler D. WAIS-III (3rd edition). Amsterdam: Harcourt Test Publishers. 2004.

138. Chu M, Meyer A, Foulkes L, Kita S. Individual differences in frequency and saliency of speech-accompanying gestures: the role of cognitive abilities and empathy. J Exp Psychol Gen. 2014; 143(2):694–709. https://doi.org/10.1037/a0033861 PMID: 23915128

139. Berch DB, Krikorian R, Huha EM. The Corsi block-tapping task: methodological and theoretical considerations. Brain Cogn. 1998; 38(3):317–38. https://doi.org/10.1006/brcg.1998.1039 PMID: 9841789

140. Raven J, Raven JC, Court JH. Raven manual section 4: advanced progressive matrices. Oxford, UK: Oxford Psychologists Press. 1998.

141. Dunn LM, Dunn D. Peabody Picture Vocabulary Test (3rd Edition). Circle Pines: American Guidance Service. 1997.

142. Schlichting L. Peabody Picture Vocabulary Test Dutch-III-NL. Amsterdam, NL: Harcourt Assessment BV. 2005.

143. Brysbaert M, Sui L, Dirix N, Hintz F. Dutch Author Recognition Test. J Cogn. 2020; 3(1):6-. https://doi.org/10.5334/joc.95 PMID: 32259014

144. Hubers F, Snijders T, Hoop H. How the brain processes violations of the grammatical norm: An fMRI study. Brain and Language. 2016; 163:22–31. https://doi.org/10.1016/j.bandl.2016.08.006 PMID: 27639117

145. Shao Z, Janse E, Visser K, Meyer AS. What do verbal fluency tasks measure? Predictors of verbal fluency performance in older adults. Frontiers in psychology. 2014; 5:772-. https://doi.org/10.3389/fpsyg.2014.00772 PMID: 25101034

146. Mainz N, Shao Z, Brysbaert M, Meyer AS. Vocabulary knowledge predicts lexical processing: Evidence from a group of participants with diverse educational backgrounds. Front Psychol. 2017; 8:1164. https://doi.org/10.3389/fpsyg.2017.01164 PMID: 28751871

147. Jongman S, Khoe Y, Hintz F. Vocabulary size influences spontaneous speech in native language users: validating the use of automatic speech recognition in individual differences research. Language and Speech. 2020:002383092091107. https://doi.org/10.1177/0023830920911079 PMID: 32223517

148. Huettig F, Janse E. Individual differences in working memory and processing speed predict anticipatory spoken language processing in the visual world. Language, Cognition and Neuroscience. 2016; 31(1):80–93.

149. Altmann GTM, Kamide Y. Incremental interpretation at verbs: restricting the domain of subsequent reference. Cognition. 1999; 73(3):247–64. https://doi.org/10.1016/s0010-0277(99)00059-1 PMID: 10585516

150. Hintz F, Meyer AS, Huettig F. Predictors of verb-mediated anticipatory eye movements in the visual world. J Exp Psychol Learn Mem Cogn. 2017; 43(9):1352–74. https://doi.org/10.1037/xlm0000388 PMID: 28287762

151. Kahneman D, Deaton A. High income improves evaluation of life but not emotional well-being. Proc Natl Acad Sci U S A. 2010; 107(38):16489–93. https://doi.org/10.1073/pnas.1011492107 PMID: 20823223

152. Biswas A, Oh PI, Faulkner GE, Bajaj RR, Silver MA, Mitchell MS, et al. Sedentary time and its association with risk for disease incidence, mortality, and hospitalization in adults: a systematic review and meta-analysis. Ann Intern Med. 2015; 162(2):123–32. https://doi.org/10.7326/M14-1651 PMID: 25599350

153. Ekelund U, Steene-Johannessen J, Brown WJ, Fagerland MW, Owen N, Powell KE, et al. Does physical activity attenuate, or even eliminate, the detrimental association of sitting time with mortality? A harmonised meta-analysis of data from more than 1 million men and women. The Lancet. 2016; 388 (10051):1302–10. https://doi.org/10.1016/S0140-6736(16)30370-1 PMID: 27475271

154. Diaz KM, Howard VJ, Hutto B, Colabianchi N, Vena JE, Safford MM, et al. Patterns of sedentary behavior and mortality in U.S. middle-aged and older adults: a national cohort study. Ann Intern Med. 2017; 167(7):465–75. https://doi.org/10.7326/M17-0212 PMID: 28892811

155. ten Broeke P, Olthof M, Beckers DGJ, Hopkins ND, Graves LEF, Carter SE, et al. Temporal dynamics of sitting behavior at work. Proceedings of the National Academy of Sciences. 2020; 117(26):14883–9. https://doi.org/10.1073/pnas.2001284117 PMID: 32541057

156. den Broeder L, Devilee J, van Oers H, Schuit AJ, Wagemakers A. Citizen science for public health. Health Promotion International. 2016; 33(3):505–14.

157. Bevelander KE, Kaipainen K, Swain R, Dohle S, Bongard JC, Hines PDH, et al. Crowdsourcing novel childhood predictors of adult obesity. PLOS ONE. 2014; 9(2):e87756. https://doi.org/10.1371/journal.pone.0087756 PMID: 24505310

158. Bongard J, Hines P, Conger D, Hurd P, Lu Z. Crowdsourcing predictors of behavioral outcomes. IEEE Transactions on Systems, Man, and Cybernetics Part A:Systems and Humans. 2012; 43.

159. Wang C, Han L, Stein G, Day S, Bien-Gund C, Mathews A, et al. Crowdsourcing in health and medical research: a systematic review. Infect Dis Poverty. 2020; 9(1):8. https://doi.org/10.1186/s40249-020-0622-9 PMID: 31959234

160. Mount M, Round H, Pitsis TS. Design thinking inspired crowdsourcing: Toward a generative model of complex problem solving. California Management Review. 2020; 62(3):103–20.

161. Van Essen DC, Ugurbil K, Auerbach E, Barch D, Behrens TE, Bucholz R, et al. The Human Connectome Project: a data acquisition perspective. Neuroimage. 2012; 62(4):2222–31. https://doi.org/10.1016/j.neuroimage.2012.02.018 PMID: 22366334

162. Miller KL, Alfaro-Almagro F, Bangerter NK, Thomas DL, Yacoub E, Xu J, et al. Multimodal population brain imaging in the UK Biobank prospective epidemiological study. Nat Neurosci. 2016; 19(11):1523–36. https://doi.org/10.1038/nn.4393 PMID: 27643430

163. Bloem BR, Marks WJ Jr., Silva de Lima AL, Kuijf ML, van Laar T, Jacobs BPF, et al. The Personalized Parkinson Project: examining disease progression through broad biomarkers in early Parkinson's disease. BMC Neurol. 2019; 19(1):160. https://doi.org/10.1186/s12883-019-1394-3 PMID: 31315608

164. Doherty A, Jackson D, Hammerla N, Plötz T, Olivier P, Granat MH, et al. Large scale population assessment of physical activity using wrist worn accelerometers: The UK Biobank Study. PLOS ONE. 2017; 12(2):e0169649. https://doi.org/10.1371/journal.pone.0169649 PMID: 28146576

165. Koolhaas CM, van Rooij FJA, Schoufour JD, Cepeda M, Tiemeier H, Brage S, et al. Objective measures of activity in the elderly: Distribution and associations with demographic and health factors. J Am Med Dir Assoc. 2017; 18(10):838–47. https://doi.org/10.1016/j.jamda.2017.04.017 PMID: 28602617

166. https://biobank.ctsu.ox.ac.uk/crystal/label.cgi?id=1008.
